# Supplementary material for: Understanding current practice, identifying barriers and exploring priorities for adverse event analysis in randomised controlled trials: an online, cross-sectional survey of statisticians from academia and industry
Source: BMJ Open. 2020 Jun 11;10(6):e036875. doi: 10.1136/bmjopen-2020-036875 (PMC7295403; doi:10.1136/bmjopen-2020-036875)
Supplement: Supplementary data [file bmjopen-2020-036875supp001.pdf]

Appendix

Item 1: Survey questions

Study Title: Statisticians survey on statistical methods for adverse event data analysis in randomised controlled trials

This survey pertains to the final analysis of AEs reported or screened for in clinical trials. Not predefined specific single safety outcomes of interest or interim analyses.

Number	Question	Response options				
1	How long have you worked as a clinical trial statistician? (Please specify the number of years)					
2	Do you work for:	Academic institution	NHS trust	Pharmaceutical company	Clinical Research Organisation	Other (please specify)
3	Is there a clinical area you predominantly work on? If yes, please specify	No	Yes			
4	What is the typical size of the trials you work on?	1-10	11-50	51-100	101-500	>500
5	What is the typical phase of the trials you work on?	Phase I/Dose-finding	Phase II/III	Phase IV		

Before you proceed we thought it would be helpful for you to know about our recent findings.

We undertook a systematic review of RCT journal reports and found that trials typically report AE data using frequencies (94%) and percentages (87%). They often ignore repeated events (84%) and 47% undertake hypothesis tests despite a lack of power. There is also a common practice to categorise continuous clinical and laboratory outcomes and present as frequencies and percentages (59%). A small proportion (12%) incorporated graphics into the AE analysis.

	Thinking about analysis methods for AEs:					
6	How often would you say the following influences the analysis performed?					
i	Statistician prefers simple approaches e.g. tables of frequencies and percentages	Always	Often	Not very often	Never	Don't know

Phillips R, Cornelius V. *BMJ Open* 2020; 10:e036875. doi: 10.1136/bmjopen-2020-036875

Appendix

	ii	Chief investigator prefers simple approaches e.g. tables of frequencies and percentages	Always	Often	Not very often	Never	Don't know
	iii	Journal prefers simple approaches e.g. tables of frequencies and percentages	Always	Often	Not very often	Never	Don't know
	iv	Regulator prefers simple approaches e.g. tables of frequencies and percentages	Always	Often	Not very often	Never	Don't know
	v	Trial sample size	Always	Often	Not very often	Never	Don't know
	vi	The number of different AEs experienced across the trial	Always	Often	Not very often	Never	Don't know
	vii	AE rates	Always	Often	Not very often	Never	Don't know
		Thinking about AE analysis you typically perform.					
7		In your experience the following is a barrier when analysing AEs:					
	i	Lack of awareness of appropriate methods	Strongly agree	Agree	Disagree	Strongly disagree	Don't know
	ii	Lack of knowledge to implement appropriate methods	Strongly agree	Agree	Disagree	Strongly disagree	Don't know
	iii	Lack of training opportunities to learn what methods are appropriate	Strongly agree	Agree	Disagree	Strongly disagree	Don't know
	iv	Lack of statistical software/code to implement appropriate methods	Strongly agree	Agree	Disagree	Strongly disagree	Don't know
	iv	Trial sample size	Strongly agree	Agree	Disagree	Strongly disagree	Don't know
	v	The number of different AEs experienced across the trial	Strongly agree	Agree	Disagree	Strongly disagree	Don't know
	vi	AE rates	Strongly agree	Agree	Disagree	Strongly disagree	Don't know
		Thinking about AE analysis.					
8		In your opinion:					
	i	Statisticians don't give AE data the same priority as the primary efficacy outcome	Strongly agree	Agree	Disagree	Strongly disagree	Don't know

Phillips R, Cornelius V. *BMJ Open* 2020; 10:e036875. doi: 10.1136/bmjopen-2020-036875

Appendix

	ii	Chief investigators don't give AE data the same priority as the primary efficacy outcome	Strongly agree	Agree	Disagree	Strongly disagree	Don't know
	iii	Journals don't give AE data the same priority as the primary efficacy outcome	Strongly agree	Agree	Disagree	Strongly disagree	Don't know
	iv	Regulators don't give AE data the same priority as the primary efficacy outcome	Strongly agree	Agree	Disagree	Strongly disagree	Don't know
	v	There are a lack of appropriate analysis methods	Strongly agree	Agree	Disagree	Strongly disagree	Don't know
	vi	There are a lack of examples of the use of appropriate analysis methods in the applied literature	Strongly agree	Agree	Disagree	Strongly disagree	Don't know
9		Are you aware of any published methods specifically to analyse AEs? If yes, please specify	Yes	No	Don't know		
10		If answer is 'yes' to question 9 In your opinion why are those methods not being more widely used:					
	i	Available methods are technically too complex	Strongly agree	Agree	Disagree	Strongly disagree	Don't know
	ii	Available methods are too resource intensive	Strongly agree	Agree	Disagree	Strongly disagree	Don't know
	iii	Available methods are not suitable for typical trial sample sizes	Strongly agree	Agree	Disagree	Strongly disagree	Don't know
	iv	Available methods are not suitable for the number of different AEs typically experienced across a trial	Strongly agree	Agree	Disagree	Strongly disagree	Don't know
	v	Available methods are not suitable for typical AE rates observed	Strongly agree	Agree	Disagree	Strongly disagree	Don't know
11		Are there any reasons other than those mention above why those methods are not being more widely used? If yes, please specify	Yes	No			

3

Appendix

		Thinking about available methods for AE analysis					
12		How concerned are you about the following:					
	i	Difficulties in interpreting the results/output	Not at all	Slightly concerned	Somewhat concerned	Moderately concerned	Extremely concerned
	ii	Robustness of methods	Not at all	Slightly concerned	Somewhat concerned	Moderately concerned	Extremely concerned
	iii	Acceptability of methods to chief investigator	Not at all	Slightly concerned	Somewhat concerned	Moderately concerned	Extremely concerned
	iv	Acceptability of methods to journal	Not at all	Slightly concerned	Somewhat concerned	Moderately concerned	Extremely concerned
	v	Acceptability of methods to regulator	Not at all	Slightly concerned	Somewhat concerned	Moderately concerned	Extremely concerned
13		Do you have any other thoughts about current practice for AE analysis? If yes, please specify	Yes	No			
14		To what extent do you agree that the following would support a change in AE analysis practice					
	i	Software/code development is needed	Strongly agree	Agree	Disagree	Strongly disagree	Don't know
	ii	Training specifically for AE analysis is needed	Strongly agree	Agree	Disagree	Strongly disagree	Don't know
	iii	Guidance on appropriate AE analysis is needed e.g. case studies, tutorials within open access journals	Strongly agree	Agree	Disagree	Strongly disagree	Don't know
15		Are there any other solutions in addition to those above that would support a change in AE analysis practice? If yes, please specify	Yes	No			
16		When analysing AEs do you present (please select all that apply):					

Phillips R, Cornelius V. *BMJ Open* 2020; 10:e036875. doi: 10.1136/bmjopen-2020-036875

Appendix

	i	Number of participants with at least one event	Yes	No
	ii	Number of events	Yes	No
	iii	Other	Yes	No
		If yes, please specify		
17		When analysing AEs which summary statistic would you typically use (please select all that apply)		
	i	Frequency	Yes	No
	ii	Percentage	Yes	No
	iii	Risk difference	Yes	No
	iv	Odds ratio	Yes	No
	v	Risk ratio	Yes	No
	vi	Incidence rate ratio	Yes	No
	vii	Other	Yes	No
		If yes, please specify		
18		In your experience how are AE rates typically compared between treatment groups (please select all that apply)		
	i	Subjective comparison	Yes	No
	ii	Exclusion of null through 95% confidence interval	Yes	No
	iii	Hypothesis test/p-value	Yes	No
	iv	Other	Yes	No
		If yes, please specify		
19		Have you undertaken any specialist AE analysis not mentioned in your previous responses? Please explain your answer. If 'yes', please include details of the method(s) used for the analysis performed	Yes	No

Phillips R, Cornelius V. *BMJ Open* 2020; 10:e036875. doi: 10.1136/bmjopen-2020-036875

Appendix

Item 2: Text from participant information sheet for CTU participants

Study Title: Statisticians survey on statistical methods for adverse event data analysis in randomised controlled trials

What is the purpose of this study?

This survey will allow an exploration of awareness of statistical methods available to flag AEs as potential adverse drug reactions (ADRs) and identify any potential barriers to their use, as well as gain feedback on ideas for new statistical methods.

Why have I been chosen?

You are eligible to participate in the survey if you satisfy the following inclusion criteria:

i) Your current role is as a senior statistician or equivalent at a UKCRC CTU;
ii) You have experience of planning and preparing final analysis reports for pharmacological RCTs.

We ask you to provide your personal views.

Do I have to take part?

Participation in the study is voluntary. It is up to you to decide whether to take part. If you decide to take part, you are still free to withdraw at any time without having to give a reason. However, retraction or removal of your survey answers is not possible once the 'Submit' button has been selected.

What are the possible disadvantages and risks of taking part?

There are no disadvantages that we are aware of from taking part in this study.

What if something goes wrong?

We are not aware of any risks involved in taking part in this study.

Will my taking part in this study be kept confidential?

All personal records relating to this study will be kept confidential. We will use SurveyMonkey to capture your responses. No personal data will be collected in the survey, as such your

Phillips R, Cornelius V. *BMJ Open* 2020; 10:e036875. doi: 10.1136/bmjopen-2020-036875

Appendix

responses to this survey will be anonymous. Responses will be kept in a secure password-protected and encrypted file and stored on Box cloud content management platform. Data in Box is stored securely and automatically backed up. The Box platform is fully General Data Protection Regulation (GDPR) compliant. Upon completion of the study the research data will be uploaded to an approved data-sharing repository. This will be maintained for at least ten years from the time the research study is complete.

What will happen to the results of the research study?

The results of this study will be analysed and published in an open access peer reviewed scientific journal. The work will also be submitted for oral presentation at a range of academic conferences targeting statisticians and the wider clinical trial community. If you would like help in locating and viewing the published results please contact us using the details below. Study data will be stored for ten years post end of study in keeping with Imperial College London research policy.

No identifying data will be published.

Will I receive payment for participating in the study?

You will not be paid for taking part in this study but upon successful completion of the survey, you will be entered into a prize draw for a chance to win £50 worth of Amazon vouchers.

Who is organising and funding the research?

This study is being organised and sponsored by Imperial College London. This study is funded by the National Institute for Health Research (NIHR) (grant reference number DRF-2017-10-131). Please note that the views expressed are those of the author(s) and not necessarily those of the NIHR or the Department of Health and Social Care.

Who has reviewed this study?

This study has been reviewed by the Head of Imperial Clinical Trials Unit and granted ethical approval by the Imperial College Joint Research Compliance Office (JRCO).

What action is required?

Appendix

Please follow the link in the invitation email to access the survey. We approximate that the survey will take no longer than 15 minutes to complete. You will have an eight-week window to complete the survey. Reminder emails will be sent at week 4 and week 6.

Please note that completing the survey and clicking 'Submit' automatically implies your consent to participate. Participation is voluntary and you are free to withdraw at any point whilst completing the survey. However please note retraction or removal of individual survey answers is not possible once the 'Submit' button has been selected.

Contact information:

Should you have any questions concerning this study, please contact the research team using the details provided below:

Rachel Phillips
Imperial Clinical Trials Unit, Imperial College London, 1st Floor Stadium House, 68 Wood Lane, London, W12 7RH
Email: r.phillips@imperial.ac.uk
Tel: 020 759 49356

We thank you for your consideration to participate in this project.

Appendix

Figure A1: Flow diagram of participation

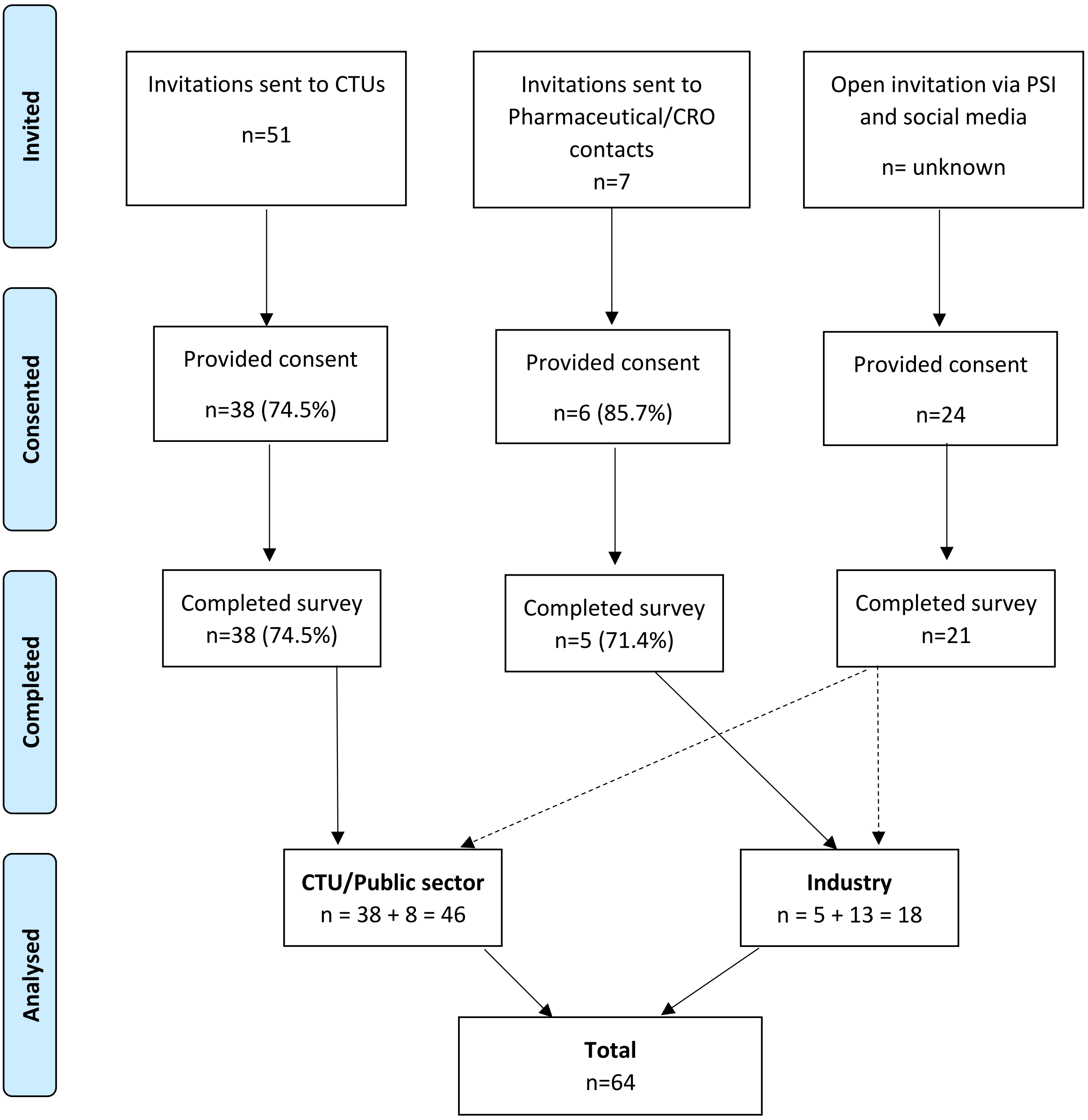

Appendix

Table A1: Free text comments regarding other information presented on adverse events (AE)

**Other information presented**
We present as a proportion as ITT and also as proportion exposed (requirement for EudraCT). We present specific toxicities and the proportions at each grade.
Number of patients with at least one G3+ events Number of patients with at least one treatment emergent Events of special interest
maximum grade over treatment by subject
Number of participants by worst grade of event (CTCAE), time to specified toxicity event
Number of events by highest CTCAE grade
Frequency of worst CTCAE grade of each AE for each patient during the treatment and follow-up periods
More frequently reported Events by severity SAEs
Relatedness
Number of events presented only for overall summary of aes, teaes, related aes and aes leading to treatment discontinuation. No summary of number of aes by soc and pt
Numbers of patients experiencing 0, 1, 2, ... events
Dependant on the trial. Most commonly the "Number of participants with at least one event" (sometimes by different treatment periods if appropriate). For trials with lengthy "maintenance" type treatments we are moving away from this and may present things like number of AEs per patient or time experiencing certain events.
median number of events in both those experiencing at least one event and out of those randomised.
And percentage by group of course.
Dependant on the trial. Most commonly the "Number of participants with at least one event" (sometimes by different treatment periods if appropriate). For trials with lengthy "maintenance" type treatments we are moving away from this and may present things like number of AEs per patient or time experiencing certain events.
Proportions and %s, making clear what the denominators are
Sometimes both, depending on the AE
In a few occasions, the client asked for confidence intervals, or the prevalence of AEs tested across arms via a Fisher exact test. On only 1 trial in 17 years of time, time to onset analyses were required, with estimation of incidence rates abd associated CI, in person-years.
Rate over the periid of exposure.
Usually both of above and incidence rate. For some events we also include rate per 100 PY
exposure time in years + incidence rates (though this varies from study to study)
incidences per group, incidence rate ratios with uncertainty (depending on the situation)
competing risk analysis

Phillips R, Cornelius V. *BMJ Open* 2020; 10:e036875. doi: 10.1136/bmjopen-2020-036875

Appendix

Table A2: Free text comments regarding methods participants are aware of specifically for adverse event (AE) analysis

**Bayesian approaches (n=1):**
"Bayesian methods to analyse low frequency event data."
**Modelling approaches (n=6):**
"I don't think there is anything special about AEs/SAEs that require special methods. Statistical methods for the analysis of events (yes/no) or repeated events accounted for differential follow-up or/and overdispersion already exist in statistical literature (e.g., poisson or negative binomial regression model). of course, it depends on the underlying distribution"
"Classical Poisson/Negative Binomial/ZIP Regression for incidence rates"
"Extreme Value methods"
"…,survival analysis for comparison of treatment and for time to specific event"
"Survival methods"
"GEE"
**Incidence rate (n=5):**
"crude incidence rates, exposure-adjusted incidence rates, mean cumulative function (MCF)"
"Rate analyses,…"
"Cumulative incidence plots, life table plots, and prevalence plots. Many methods not specific to analysis of AEs"
"Incidence rates and confidence intervals (in person-years). Time to onset."
"Rate ratio,…"
**Meta-analysis (n=2):**
"…examples of meta analyses to appropriately analyse AE data"
" Meta analysis of Rare events"
**Graphics (n=2):**
"Cumulative incidence plots, life table plots, and prevalence plots. Many methods not specific to analysis of AEs"
"Graphics for biological parameters (ellipse ci)"
**Theoretical and applied examples (n=6):**
" CLEOPATRA Study Repeated Measures (i.e. not just counting first event)"
"Various methods published by Harry Southworth. These are predominantly useful for pharma trials rather than Phase 4 trials unit trials."
"Volume15, Issue4 Special Issue: Analysis of Adverse Event Data July/August 2016 Pages 297-305"
"http://dx.doi.org/10.1136/bmj.i5078"
"https://onlinelibrary.wiley.com/toc/15391612/2016/15/4"
"possible use of estimands to analyse AEs (for example https://arxiv.org/abs/1805.01834)"
**Other comments:**
"Not meaningfully within an early phase setting, because of sample size. Monitoring based approaches are becoming used and machine learning based methods are available."
"AE tables and summary"
"The statistical literature is awash with methods"
"zz"

Phillips R, Cornelius V. BMJ Open 2020; 10:e036875. doi: 10.1136/bmjopen-2020-036875

Appendix

Table A3: Free text comments regarding participants' use of specialist methods for adverse event (AE) analysis

**Time-to-event analysis (n=2):**
"In characterising safety signals I have used Time to Event, Event rates, prevalence."
"Time-to-event analyses; exposure-adjusted AE rates"
**Data visualisations (n=1):**
"Data visualisation (which is more or less equivalent to frequencies and percentages)"
**Bayesian methods**
"Bayesian methods for sparse adverse events data meta-analysis"
**Incorporating repeated event (n=1):**
"For within-patient repeated events we have produced comparisons with a 2-d frequency table (arm vs # events)"
**Other comments:**
"Not sure I understood what is meant by specialist AE analysis. I used various statistical methods depending on the situation."
"Safety analysis in phase III cancer clinical trial"

Table A4: Reasons specialist adverse event (AE) methods are not used (of participants aware of such methods)

**Reasons for unsuitability. Available methods are:**		**CTU/Public (N=11)**		**Industry (N=12)**		**Overall (N=23)**	
		**n/N**	**%**	**n/N**	**%**	**n/N**	**%**
Technically too complex	Strongly disagree/disagree	8/10	80.0%	6/12	50.0%	14/22	63.6%
	Agree/strongly agree	1/10	10.0%	5/12	41.7%	6/22	27.3%
	Don't know	1/10	10.0%	1/12	8.3%	2/22	9.1%
Too resource intensive	Strongly disagree/disagree	5/10	50.0%	7/12	58.3%	12/22	54.5%
	Agree/strongly agree	5/10	50.0%	5/12	41.7%	10/22	45.5%
Not suitable for typical trial sample sizes	Strongly disagree/disagree	6/10	60.0%	4/12	33.3%	10/22	45.5%
	Agree/strongly agree	3/10	30.0%	5/12	41.7%	8/22	36.4%
	Don't know	1/10	10.0%	3/12	25.0%	4/22	18.2%
Not suitable for the number of different AEs typically experienced across a trial	Strongly disagree/disagree	7/10	70.0%	5/12	41.7%	12/22	54.5%
	Agree/strongly agree	2/10	20.0%	6/12	50.0%	8/22	36.4%
	Don't know	1/10	10.0%	1/12	8.3%	2/22	9.1%
Not suitable for typical AE rates observed	Strongly disagree/disagree	7/10	70.0%	5/12	41.7%	12/22	54.5%
	Agree/strongly agree	3/10	30.0%	7/12	58.3%	10/22	45.5%
Other reasons why those methods are not used	No	0/10	0.0%	3/12	25.0%	3/22	13.6%
	Yes	9/10	90.0%	8/12	66.7%	17/22	77.3%
	Don't know	1/10	10.0%	1/12	8.3%	2/22	9.1%

Acronyms: CTU: Clinical Trial Unit; AE: adverse event

Phillips R, Cornelius V. BMJ Open 2020; 10:e036875. doi: 10.1136/bmjopen-2020-036875

Appendix

Table A5: Classification of participants' comments on the reasons for a lack of use of specialist methods for adverse event (AE) analysis

Classification of reasons given for the lack of use of specialist AE analysis methods	Participant comment
**1.Concern with the suitability of methods in relation to trial design characteristics and nature of AE data**	*"…These analyses methods may also not be appropriate if there are doubts about the robustness of AE data…"* (CTU/public sector)
	"The strongest driver is sample size and multiplicity with multiple endpoints, limiting the power of any such analysis." (CTU/public sector)
	"AEs not the primary objective of trial, Pharmaceutical companies focused not on most powerful analyses, issues around multiplicity, recurrent events, low incidence of events" (Industry)
	"…Most AE signals will not result in a statistically significant difference (due to low rates and trial size) and therefore a fear of testing exists, as statisticians we do not want to give the impression that the signal is not real as p>0.05!! Few trials are designed to specifically look at safety, the above methods are used on safety studies." (Industry)
	"…safety analyses typically lack a scientific hypotheses to direct where to look for signals." (CTU/public sector)
	"…2) Multiple testing issues: The multiplicity of AEs that may arise in a RCT makes it also not really appropriate to use statistical tests because of inflated false positive error rates resulting from multiple testings. …3) Even if 1 or 2 AEs of special interest are selected for statistical testing, detecting a statistically significant difference across treatment arms requires to power the trial and calculate the sample size accordingly." (Industry)
	"Appropriateness of methods depends on many factors including underlying distribution, prevalence of repeated events, whether participants were followed up for the same duration, etc. For example, if repeated events are rare and participants were followed up for the same duration then simple number and percentages of participants who experienced at least one event is sufficient. On the contrary, this will obscure the true picture if repeated events are prevalent and participants were follows up for varying periods. So I would say there is a range of statistical methods that are appropriate depending on the situation." (CTU/public sector)
**2. Opposition and a lack of understanding from clinicians**	*"Lack of emphasis placed by clinicians on the need for appropriate statistical methods to analyse adverse events data."* (CTU/public sector)
	"The standard approach of looking at g3+ AEs only is so accepted, there is little motivation to explore other methods. In addition, persuading clinicians to embrace other methods, can be difficult." (CTU/public sector)

Phillips R, Cornelius V. *BMJ Open* 2020; 10:e036875. doi: 10.1136/bmjopen-2020-036875

Appendix

	"Most medical leads on clinical trials do not understand statistical analyses and only prefer a list of AEs with their percentages to be presented" (Industry)
	"A tendency to oversimplify reporting of safety signals, to make them easier to understand to non-stats people (e.g. % are easier than incidence rates)" (Industry)
	"The template for reporting AEs is too basic. In the pharmaceutical industry the statisticians have little to no input into the trial paper" (CTU/public sector)
**3. Not deemed to be needed by statisticians**	"*Not required/ wanted*." (CTU/public sector)
	"Don't want to report additional information in CTR" (CTU/public sector)
	"They are perhaps not used as they are no required or appropriate for that type of trial. There is no point in applying a complex method when it is not needed (eg when AEs are collected for a well established drug; when the trial is not attempting to define a safety profile)." (CTU/public sector)
**4. A desire to keep analysis consistent with historical analysis**	"*Easiness to present always the same tables"* (CTU/public sector)
	"1) High level of standardization in reporting of results of RCTs. AE tables are pretty standard and there are requirements to meet ICH3 CSR recommendations…" (Industry)
	"Consistency of analysis across trials in a development programme is often paramount. So, if AEs from a previous study have been analysed using a frequency/percentage approach, so would later trials." (Industry)
**5. Training and resources**	"*Training. Availability of code*." (Industry)

Phillips R, Cornelius V. *BMJ Open* 2020; 10:e036875. doi: 10.1136/bmjopen-2020-036875

Appendix

Table A6: Influences the analysis performed

		CTU/Public (N=45)		Industry (N=18)		Overall (N=63)	
**Influence**		**n/N**	**%**	**n/N**	**%**	**n/N**	**%**
Statistician prefers simple approaches e.g. tables of frequencies and percentages	Never/Not very often	13/45	28.9%	7/18	38.9%	20/63	31.7%
	Often/Always	32/45	71.1%	11/18	61.1%	43/63	68.3%
Chief investigator prefers simple approaches e.g. tables of frequencies and percentages	Never/Not very often	9/45	20.0%	2/18	11.1%	11/63	17.5%
	Often/Always	35/45	77.8%	14/18	77.8%	49/63	77.8%
	Don't know	1/45	2.2%	2/18	11.1%	3/63	4.8%
Journal prefers simple approaches e.g. tables of frequencies and percentages	Never/Not very often	12/45	26.7%	7/18	38.9%	19/63	30.2%
	Often/Always	25/45	55.6%	5/18	27.8%	30/63	47.6%
	Don't know	8/45	17.8%	6/18	33.3%	14/63	22.2%
Regulator prefers simple approaches e.g. tables of frequencies and percentages	Never/Not very often	9/45	20.0%	4/18	22.2%	13/63	20.6%
	Often/Always	18/45	40.0%	12/18	66.7%	30/63	47.6%
	Don't know	18/45	40.0%	2/18	11.1%	20/63	31.7%
Trial sample size	Never/Not very often	12/45	26.7%	2/18	11.1%	14/63	22.2%
	Often/Always	31/45	68.9%	15/18	83.3%	46/63	73.0%
	Don't know	2/45	4.4%	1/18	5.6%	3/63	4.8%
The number of different AEs experienced across the trial	Never/Not very often	13/45	28.9%	7/18	38.9%	20/63	31.7%
	Often/Always	31/45	68.9%	10/18	55.6%	41/63	65.1%
	Don't know	1/45	2.2%	1/18	5.6%	2/63	3.2%
AE rates	Never/Not very often	9/45	20.0%	4/18	22.2%	13/63	20.6%
	Often/Always	35/45	77.8%	13/18	72.2%	48/63	76.2%
	Don't know	1/45	2.2%	1/18	5.6%	2/63	3.2%

Acronyms: CTU: Clinical Trial Unit; AE: adverse event

Phillips R, Cornelius V. *BMJ Open* 2020; 10:e036875. doi: 10.1136/bmjopen-2020-036875

Appendix

Table A7: Barriers when analysing adverse events (AEs)

		CTU/Public (N=44)		Industry (N=18)		Overall (N=62)	
**Barriers**		**n/N**	**%**	**n/N**	**%**	**n/N**	**%**
Lack of awareness of appropriate methods	Strongly disagree/disagree	11/44	25.0%	7/18	38.9%	18/62	29.0%
	Agree/ Strongly agree	30/44	68.2%	11/18	61.1%	41/62	66.1%
	Don't know	3/44	6.8%	0/18	0.0%	3/62	4.8%
Lack of knowledge to implement appropriate methods	Strongly disagree/disagree	15/44	34.1%	8/18	44.4%	23/62	37.1%
	Agree/ Strongly agree	27/44	61.4%	9/18	50.0%	36/62	58.1%
	Don't know	2/44	4.5%	1/18	5.6%	3/62	4.8%
Lack of training opportunities to learn what methods are appropriate	Strongly disagree/disagree	7/44	15.9%	3/18	16.7%	10/62	16.1%
	Agree/ Strongly agree	34/44	77.3%	15/18	83.3%	49/62	79.0%
	Don't know	3/44	6.8%	0/18	0.0%	3/62	4.8%
Lack of statistical software/code to implement appropriate methods	Strongly disagree/disagree	21/44	47.7%	11/18	61.1%	32/62	51.6%
	Agree/ Strongly agree	14/44	31.8%	7/18	38.9%	21/62	33.9%
	Don't know	9/44	20.5%	0/18	0.0%	9/62	14.5%
Trial sample size	Strongly disagree/disagree	13/44	29.5%	7/18	38.9%	20/62	32.3%
	Agree/ Strongly agree	27/44	61.4%	11/18	61.1%	38/62	61.3%
	Don't know	4/44	9.1%	0/18	0.0%	4/62	6.5%
The number of different AEs experienced across the trial	Strongly disagree/disagree	15/44	34.1%	7/18	38.9%	22/62	35.5%
	Agree/ Strongly agree	27/44	61.4%	11/18	61.1%	38/62	61.3%
	Don't know	2/44	4.5%	0/18	0.0%	2/62	3.2%
AE rates	Strongly disagree/disagree	14/44	31.8%	7/18	38.9%	21/62	33.9%
	Agree/ Strongly agree	29/44	65.9%	11/18	61.1%	40/62	64.5%
	Don't know	1/44	2.3%	0/18	0.0%	1/62	1.6%

Acronyms: CTU: Clinical Trial Unit; AE: adverse event

Phillips R, Cornelius V. *BMJ Open* 2020; 10:e036875. doi: 10.1136/bmjopen-2020-036875

Appendix

Table A8: Opinions on adverse event (AE) analysis

		CTU/Public (N=44)		Industry (N=18)		Overall (N=62)	
**Opinions**		**n/N**	**%**	**n/N**	**%**	**n/N**	**%**
Statisticians don't give AE data the same priority as the primary efficacy outcome	Strongly disagree/disagree	13/44	29.5%	6/18	33.3%	19/62	30.6%
	Agree/strongly agree	31/44	70.5%	12/18	66.7%	43/62	69.4%
Chief investigators don't give AE data the same priority as the primary efficacy outcome	Strongly disagree/disagree	20/44	45.5%	7/18	38.9%	27/62	43.5%
	Agree/strongly agree	24/44	54.5%	8/18	44.4%	32/62	51.6%
	Don't know	0/44	0.0%	3/18	16.7%	3/62	4.8%
Journals don't give AE data the same priority as the primary efficacy outcome	Strongly disagree/disagree	12/44	27.3%	4/18	22.2%	16/62	25.8%
	Agree/strongly agree	26/44	59.1%	11/18	61.1%	37/62	59.7%
	Don't know	6/44	13.6%	3/18	16.7%	9/62	14.5%
Regulators don't give AE data the same priority as the primary efficacy outcome	Strongly disagree/disagree	25/44	56.8%	14/18	77.8%	39/62	62.9%
	Agree/strongly agree	5/44	11.4%	3/18	16.7%	8/62	12.9%
	Don't know	14/44	31.8%	1/18	5.6%	15/62	24.2%
There are a lack of appropriate analysis methods	Strongly disagree/disagree	15/44	34.1%	8/18	44.4%	23/62	37.1%
	Agree/strongly agree	19/44	43.2%	8/18	44.4%	27/62	43.5%
	Don't know	10/44	22.7%	2/18	11.1%	12/62	19.4%
There are a lack of examples of the use of appropriate analysis methods in the applied literature	Strongly disagree/disagree	5/44	11.4%	1/18	5.6%	6/62	9.7%
	Agree/strongly agree	36/44	81.8%	16/18	88.9%	52/62	83.9%
	Don't know	3/44	6.8%	1/18	5.6%	4/62	6.5%

Acronyms: CTU: Clinical Trial Unit; AE: adverse event

Phillips R, Cornelius V. *BMJ Open* 2020; 10:e036875. doi: 10.1136/bmjopen-2020-036875

Appendix

Table A9: Concerns about available methods for adverse event (AE) analysis

		CTU/Public (N=43)		Industry (N=17)		Overall (N=60)	
**Concerns**		**n/N**	**%**	**n/N**	**%**	**n/N**	**%**
Difficulties in interpreting the results/output	Not at all to somewhat concerned	34/43	79.1%	11/17	64.7%	45/60	75.0%
	Moderately to extremely concerned	9/43	20.9%	6/17	35.3%	15/60	25.0%
Robustness of methods	Not at all to somewhat concerned	29/43	67.4%	12/17	70.6%	41/60	68.3%
	Moderately to extremely concerned	14/43	32.6%	5/17	29.4%	19/60	31.7%
Acceptability of methods to chief investigator	Not at all to somewhat concerned	36/43	83.7%	12/17	70.6%	48/60	80.0%
	Moderately to extremely concerned	7/43	16.3%	5/17	29.4%	12/60	20.0%
Acceptability of methods to journal	Not at all to somewhat concerned	34/43	79.1%	14/17	82.4%	48/60	80.0%
	Moderately to extremely concerned	9/43	20.9%	3/17	17.6%	12/60	20.0%
Acceptability of methods to regulator	Not at all to somewhat concerned	33/43	76.7%	4/17	23.5%	37/60	61.7%
	Moderately to extremely concerned	10/43	23.3%	13/17	76.5%	23/60	38.3%

Acronyms: CTU: Clinical Trial Unit; AE: adverse event

Phillips R, Cornelius V. *BMJ Open* 2020; 10:e036875. doi: 10.1136/bmjopen-2020-036875

Appendix

Table A10: Solutions to support a change in adverse event (AE) analysis practice

		CTU/Public (N=43)		Industry (N=17)		Overall (N=60)	
**Change**		**n/N**	**%**	**n/N**	**%**	**n/N**	**%**
Software/code development is needed	Strongly disagree/disagree	9/43	20.9%	6/17	35.3%	15/60	25.0%
	Agree/strongly agree	28/43	65.1%	10/17	58.8%	38/60	63.3%
	Don't know	6/43	14.0%	1/17	5.9%	7/60	11.7%
Training specifically for AE analysis is needed	Strongly disagree/disagree	1/43	2.3%	1/17	5.9%	2/60	3.3%
	Agree/strongly agree	42/43	97.7%	16/17	94.1%	58/60	96.7%
Guidance on appropriate AE analysis is needed e.g. case studies, tutorials within open access journals	Strongly disagree/disagree	0/43	0.0%	0/17	0.0%	0/60	0.0%
	Agree/strongly agree	43/43	100.0%	17/17	100.0%	60/60	100.0%
Are there any other solutions in addition to those stated above that would support a change in AE analysis practice?	No	34/43	79.1%	7/17	41.2%	41/60	68.3%
	Yes	9/43	20.9%	10/17	58.8%	19/60	31.7%

Acronyms: CTU: Clinical Trial Unit; AE: adverse event

Phillips R, Cornelius V. *BMJ Open* 2020; 10:e036875. doi: 10.1136/bmjopen-2020-036875

Appendix

Table A11: Classification of participants' comments on solutions to support change in adverse event (AE) analysis practices

Classification of solutions to support a change in AE analysis practice	Participant comment
**1. Improved standards or calls for changes from journals, registries and regulators**	*"Influencing journals to pay more attention to this"* (CTU)
	"…we presented incidences because they represented a fairer picture due to differential follow-up and repeated incidences per person. The reviewer and the editor said they prefer proportions and don't understand what we presented. I explained in lay terms and pushed back their request because it was flawed. This shows that Statisticians can defend a certain position and educate others even if they have their own preferences.<br><br>*Regulatory repositories/registries such as EUDRACT has a fixed format of presenting results so you have to go with what is required even though you know it's flawed in certain situation. Flexibility of such registries is very important to allow people to present both proportions and incidences where appropriate."* (CTU)
	"Asked by the authorities" (Industry)
	"Strong regulatory direction is always good for changing practices within the industry!" (Industry)
	"engaging the … regulators" (Industry)
	"*The biggest driver of a change in behaviour is usually a regulator requesting it.*" (Industry)
	"Regulators to be more demanding in analytical approaches, don't require more than summaries. That's far removed from discussions on efficacy" (Industry)
	"Would have to be able to upload the results to EUDRACT for CTIMPS." (CTU)
**2. Development of guidance, education and engaging with the medical community**	*"Best practice guidance although that would depend on trial type and phase, sample size, whether only SAEs/related AEs are being captured/important, ... particularly important to reflect on complex interventions vs CTIMP, etc"* (CTU)
	"There needs to be consensus that a change is needed. What are the issues in current AE reporting? There needs to be better guidance re collection of AE data. Can we collect it in a more robust way? We need to differentiate between examining pre-specified hypotheses and trying to identify issues we don't know about (eg in early phase trials). We need agreement re standards for different phases and types of trials (eg Phase 1 vs Phase 4, explanatory vs

Phillips R, Cornelius V. BMJ Open 2020; 10:e036875. doi: 10.1136/bmjopen-2020-036875

Appendix

	pragmatic, regulatory submissions vs investigator led exploratory trials on marketed products)" (CTU)
	*"Published case studies**"** (Industry)*
	"engaging the medical community …. and Better education on the pros of using proper stats methodology. If the benefits of using effective statistical analysis methods over frequencies and percentages can be demonstrated, there might be more interest" (Industry)
	"demonstration of the benefits of these methods over existing ones, and when they are appropriate" (CTU)
	"Open discussions with clinical community (e.g. open forums, etc) on alternative methods to avoid them being scared off" (Industry)
	More focus on safety analyses in the E9 addendum" (Industry)
	"Application of CONSORT harms" (CTU)
	"Evolution of standard reporting requirements in clinical trials (ICH E3, and maybe CONSORT Statement ?)" (Industry)
**3. Analysis**	*"IPD meta analysis of AEs" (CTU)*
	"In addition to 'methods' there perhaps need to be discussion about populations/datasets on which to base AE analyses." (CTU)
	"Inferential analysis based on small numbers of adverse events, but of great influence on the patient health." (Industry)

Phillips R, Cornelius V. *BMJ Open* 2020; 10:e036875. doi: 10.1136/bmjopen-2020-036875

Appendix

Table A12: Classification of participants' general comments raised regarding adverse event (AE) analysis practices

Classification of suggestions raised for AE analysis	Participant comment
**1. Minimum summary information participants would expect to be reported for AEs**	*"Different analysis approach are useful for interpretation when reporting AEs/SAEs. As a starting point, I would like to know the numbers and proportions experiencing at least one SAE by group, between group differences with uncertainty. In addition, I would like to know the incidences per group and incidence rate ratio with uncertainty. The later is not always necessary depending on the situation.."* (CTU)
	"I think in general reporting numbers and percentages is appropriate. The argument being that, if we were clinicians or patients we would want to know what is the chances of me having this event and how bad will it get, which is essentially what the frequency tables give you." (CTU)
**2. Changes that could or have been made to analysis practice**	*"No best practice guidance although revised CONSORT does help remind of importance of AE reporting" (*CTU*)*
	"There was a great talk at SCT 2017 on using graphical methods to summarise AEs and I have been trying to implement graphical methods to summarise the many dimensions of AE reporting as a way forward" (CTU*)*
	"Use of graphical methods in reporting to compare treatments ought to be standard, as per BMJ article. They are easy enough to apply…<br>*…The format of the source data, typically free text, is a pain to code into MedDRA. Methods to make this easier would be very valuable: some sort of AI machine learning maybe?...*<br>*…Meta-analysis should be very important to apply to safety data, given how under-powered individual trials may be for safety comparisons. Finding tools to automate, maybe using results entered on EudraCT might be an idea."* (CTU)
	"We have increased our use of graphics. I find benefit risk plots a very powerful way of summarising data. Allows key efficacy and safety to be displayed on one page and is a really useful summary of a drug's profile." (Industry*)*
	"Current practice will need to turn to methods of detecting signals as real-time data come from trials." (Industry)
	"Signal detection method" (CTU)

Phillips R, Cornelius V. *BMJ Open* 2020; 10:e036875. doi: 10.1136/bmjopen-2020-036875

Appendix

	"I'm interested in knowing more about risk factors of occurrence of serious or really frequent AEs of chemotherapies, beyond receiving protocol x." (Industry)
	"… not many medical leads understand statistical analysis of AEs or count or rate data and only insist on percentages and frequencies. Better methods exist but are not utilised due to lack of knowledge of PIs or medical advisors" (Industry)
**3. Concerns about the quality and collection of AE data**	*"This definitely gets overlooked. I always worry about how systematically the data have been collected too as well as the validity of lumping very different events together in the same analysis."* (CTU)
	"I think a big factor in what analysis we choose is how the data is collected. If the data is not detailed enough some only simple methods may be appropriate - this has often been my feeling when analysing our data. this may change in current/future trials as we are changing how we collect some AE data" (CTU)
	"My concerns start with the quality of AE data collected. Is it complete? Is it robust? There is recall bias, variability between centres, investigators etc. There may also be variability with respect to coding. We all have experience of stating up front what should NOT be recorded as AE, to see such things recorded multiple times. One of my major concerns is the listing of AEs each with associated p-values (obviously the CI would insist on this and not the statistician). Completely meaningless as it doesn't take into account sample size, rate, number of events within a participants, severity of event etc etc. Also of concern is the use of more complex methodologies on such data as it implies that the data are robust. I think that the simple approach is often acceptable so long as the data are presented in different ways (see Q16). The main issue is about defining what you are trying to detect from the collection of AE data. If we can do this better then perhaps additional required methodology will come." (CTU)
**4. General comments and criticisms about current AE analysis and reporting practices**	*"Somewhat arbitrary grouping of AEs. Not always clear whether numbers are subjects or events are presented in published papers."* (CTU)
	"In my 8.5 years of experience I have not seen many studies where they have spoken much about AE data analysis." (Industry)
	"People do the most powerful test for efficacy - no barrel goes unscraped - and the least powerful for safety" (CTU)
	"It can be improved!" (Industry)

Phillips R, Cornelius V. *BMJ Open* 2020; 10:e036875. doi: 10.1136/bmjopen-2020-036875
