# Supplementary material for: Understanding current practice, identifying barriers and exploring priorities for adverse event analysis in randomised controlled trials: an online, cross-sectional survey of statisticians from academia and industry
Source: BMJ Open. 2020 Jun 11;10(6):e036875. doi: 10.1136/bmjopen-2020-036875 (PMC7295403; doi:10.1136/bmjopen-2020-036875)
Supplement: Author's manuscript [file bmjopen-2020-036875.draft_revisions.pdf]

BMJ Open is committed to open peer review. As part of this commitment we make the peer review history of every article we publish publicly available.

When an article is published we post the peer reviewers' comments and the authors' responses online. We also post the versions of the paper that were used during peer review. These are the versions that the peer review comments apply to.

The versions of the paper that follow are the versions that were submitted during the peer review process. They are not the versions of record or the final published versions. They should not be cited or distributed as the published version of this manuscript.

BMJ Open is an open access journal and the full, final, typeset and author-corrected version of record of the manuscript is available on our site with no access controls, subscription charges or pay-per-view fees (http://bmjopen.bmj.com).

If you have any questions on BMJ Open's open peer review process please email info.bmjopen@bmj.com

BMJ Open

Understanding current practice, identifying barriers and exploring priorities to improve the analysis of AEs in RCTs: a survey of statisticians from academia and industry

Journal:	*BMJ Open*
Manuscript ID	bmjopen-2020-036875
Article Type:	Original research
Date Submitted by the Author:	08-Jan-2020
Complete List of Authors:	Phillips, Rachel; Imperial College London, Faculty of Medicine, School of Public Health<br>Cornelius, Victoria; Imperial College London, Faculty of Medicine, School of Public Health
Keywords:	Clinical trials < THERAPEUTICS, Adverse events < THERAPEUTICS, STATISTICS & RESEARCH METHODS

SCHOLARONE™
Manuscripts

I, the Submitting Author has the right to grant and does grant on behalf of all authors of the Work (as defined in the below author licence), an exclusive licence and/or a non-exclusive licence for contributions from authors who are: i) UK Crown employees; ii) where BMJ has agreed a CC-BY licence shall apply, and/or iii) in accordance with the terms applicable for US Federal Government officers or employees acting as part of their official duties; on a worldwide, perpetual, irrevocable, royalty-free basis to BMJ Publishing Group Ltd ("BMJ") its licensees and where the relevant Journal is co-owned by BMJ to the co-owners of the Journal, to publish the Work in this journal and any other BMJ products and to exploit all rights, as set out in our licence.

The Submitting Author accepts and understands that any supply made under these terms is made by BMJ to the Submitting Author unless you are acting as an employee on behalf of your employer or a postgraduate student of an affiliated institution which is paying any applicable article publishing charge ("APC") for Open Access articles. Where the Submitting Author wishes to make the Work available on an Open Access basis (and intends to pay the relevant APC), the terms of reuse of such Open Access shall be governed by a Creative Commons licence – details of these licences and which Creative Commons licence will apply to this Work are set out in our licence referred to above.

Other than as permitted in any relevant BMJ Author's Self Archiving Policies, I confirm this Work has not been accepted for publication elsewhere, is not being considered for publication elsewhere and does not duplicate material already published. I confirm all authors consent to publication of this Work and authorise the granting of this licence.

For peer review only

Understanding current practice, identifying barriers and exploring priorities to improve the analysis of AEs in RCTs: a survey of statisticians from academia and industry

Authors:

Rachel Phillips* - Imperial College London, London, United Kingdom

Victoria Cornelius - Imperial College London, London, United Kingdom

* Corresponding author

Address: Imperial Clinical Trials Unit, Imperial College London, 1st Floor Stadium House, 68 Wood Lane, London, W12 7RH

Email: r.phillips@imperial.ac.uk

Telephone: 020 759 49356

* Funded by a NIHR doctoral research fellowship to undertake this work (Reference: DRF-2017-10-131)

Word Count: 3822

For peer review only

Abstract

Objectives

To gain a better understanding of current adverse event (AE) analysis practices and the reasons for the lack of use of sophisticated statistical methods for AE data analysis in randomised controlled trials (RCTs), with the aim of identifying priorities and solutions to improve practice.

Design

A cross-sectional, online survey of statisticians working in clinical trials, followed-up with a workshop of senior statisticians working across the United Kingdom.

Participants

We aimed to recruit into the survey a minimum of one statistician from each of the 51 UK Clinical Research Collaboration (CRC) registered clinical trial units (CTUs) and industry statisticians from both pharmaceuticals and clinical research organisations (CROs).

Outcomes

To gain a better understanding of current AE analysis practices, measure awareness of specialist methods for AE analysis and explore priorities, concerns and barriers when analysing AEs.

Results

Thirty-eight (75%) CTUs, five (71%) industry and twenty-one attendees at the 2019 PSI conference consented to participate and proceeded into the survey. Forty-six participants were classified as public sector participants and eighteen as industry participants. Participants indicated that they predominantly (80%) rely on subjective comparisons when comparing AEs between treatment groups. Forty percent were aware of specialist methods for AE analysis but only 13% had undertaken such analyses. All participants believed guidance on appropriate AE analysis and 97% thought

For peer review only

training specifically for AE analysis is needed. These were both endorsed as solutions by workshop participants.

Conclusions

This research supports our earlier work that identified sub-optimal AE analysis practices in RCTs and confirms the under use of more sophisticated AE analysis approaches. Improvements are needed and this research provides a unanimous call for the development of guidance, as well as training on appropriate methods for AE analysis to support change. Further research is needed to identify the most appropriate statistical methods for AE data analysis.

Keywords

Randomised controlled trials; adverse events; harms; adverse drug reactions; survey; statisticians; clinical trials units; industry; analysis.

For peer review only

Article summary: Strengths and limitations of this study

- A high response rate was achieved from UKCRC CTU and industry statisticians invited to participate in this survey.
- There was some level of self-selection to participation and as such, there is a possibility that participants had an increased interest in adverse event (AE) analysis and are not fully representative of the clinical trial community.
- The survey was followed up with a workshop of senior statisticians from across the United Kingdom, which represents more of a general interest group.
- The survey provides insight and essential starting points to identify areas of focus to help support a change to improve AE analysis practices.

For peer review only

INTRODUCTION

Randomised controlled trials (RCTs) are a valuable source of information when establishing the harm profile of medicinal products. They provide a controlled comparison of adverse event (AE) rates, thus allowing causality to be evaluated and potential detection of adverse drug reactions (ADRs). Reviews of journal article reports of RCTs have demonstrated that harms data is not being fully utilised with frequent inappropriate and insufficient analyses.[1-4] In addition, inconsistent information is reported, thus preventing a complete summary of the harm profile to be established.[5-11†]

Building on previous work a comprehensive methods review undertaken by the authors revealed that there are a broad range of published statistical methods proposed specifically to analyse AE data for both the interim and final analysis.[12, 13] Many of the proposed methods could be adopted into current practice with relative ease. Chuang-Stein and Xia have proposed examples of industry strategies adopting such methods.[14] Previous research has demonstrated that these methods are not used for the analysis presented in the primary results publication, and there are minimal citations of these published methods in the RCT setting, which further suggests uptake of these methods is low.[1, 12, 13] Understanding the reasons for this low uptake will help identify solutions to improve the analysis of AEs in RCTs. We undertook a survey of UK statisticians working in clinical trials to investigate their current practice when analysing AEs, to measure their awareness of available methods for AE analysis, and to explore their priorities, concerns and identify any perceived barriers when analysing AEs.

[†] An adverse event is defined as 'any untoward medical occurrence that may present during treatment with a pharmaceutical product but which does not necessarily have a causal relationship with this treatment'. An adverse drug reaction (ADR) is defined as 'a response to a drug which is noxious and unintended …' where a causal relationship is 'at least a reasonable possibility'.

For peer review only

METHODS

Study design

A cross-sectional, online survey of UK Clinical Research Collaboration (CRC) clinical trial unit (CTU) and industry statisticians from both pharmaceuticals and clinical research organisations (CROs) was conducted. We aimed to recruit a minimum of one statistician from each of the 51 UKCRC registered CTUs and from a sample of pharmaceutical companies and CROs in the UK to gain an industry perspective. The survey was followed-up with a workshop at the UKCRC biannual statisticians' operations group meeting where survey results were presented and areas for improvements and priorities were discussed.

Survey development

The survey was developed using information from current guidance and previous research that examined barriers to the uptake of new methodology.[15-18] Topics covered included questions about current practice and factors influencing AE analysis performed; barriers encountered when analysing AEs; concerns regarding AE analysis; awareness and opinions of specialist methods for AE analysis; concerns and barriers of implementing specialist methods; and opinions on potential solutions to support a change in AE analysis practice.

Questions were predominantly closed form but where appropriate open-ended questions were included to allow for detailed responses and comments. Responses were measured using Likert scales.

For peer review only

Survey questions for UKCRC CTU and industry statisticians were identical (appendix item 1). The survey was piloted on clinical trial statisticians (n= 6) at three CTUs prior to launching nationwide to ensure understanding of the questions, whether sufficient response categories had been included, and if certain questions were consistently left unanswered, as well as the usability and functionality of the online platform hosted by SurveyMonkey.[19]

Sampling and Recruitment

We targeted a population that we knew to be predominantly involved in the analysis of AEs in clinical trials. Specifically, the UKCRC CTU Statistics Operation network supported the survey and contacted each of the 51 registered CTUs' senior statisticians on behalf of the study team. Email invitations were also sent directly to a convenience sample of seven senior statistical contacts working in UK based pharmaceuticals (Astra-Zeneca, Boehringe-Ingelheim, Glaxo-Smith-Kline (GSK), Novartis and Roche) and CROs (Cytel and IQVIA). The invitations requested that one statistician within the unit or organisation complete the survey. Reminder emails were sent to non-responders. The survey was open for 8 weeks. We also created an open platform for participants that was promoted at the 2019 Promoting Statistical Insights (PSI) conference, the Effective Statistician podcast, and Twitter and LinkedIn platforms. This platform remained open for 10 weeks.

Participants

Statisticians with experience of planning and preparing the final analysis reports for pharmacological RCTs were invited to participate.

For peer review only

Analysis

Descriptive analysis was undertaken, primarily including frequencies and proportions for each questionnaire item and where appropriate was accompanied with visual summaries. The frequency and proportion of participants that showed support for an item was calculated by combining the 'always' and 'often' or 'strongly agree' and 'agree' categories. Participants were classified according to affiliation into either CTU/public sector or industry sector and analysis was stratified by sector. Response rates were calculated for groups of participants where known.

Patient and public involvement

This survey forms part of a wider research project that was developed with input from a range of patient representatives. There were no patients directly involved in this survey but the original proposal and patient and public involvement (PPI) strategy were reviewed by service user representatives (with experience as clinical trial participants and PPI advisors) who provided advice specifically with regard to communication and dissemination to patient and public groups.

RESULTS

Participant flow

For peer review only

The survey opened in April 2019. Thirty-eight (75%) units and six (86%) industry contacts consented to participate in the study. One industry contact failed to complete the survey after providing consent giving an overall response rate of 74%. Twenty-four people consented to participate via the open platform, of which three failed to complete the survey after providing consent. Of the 21 participants n=8 were included in the CTU/public sector group and n=13 in the industry sector. In total 64 participants took part in the survey with n=46 from the CTU/public sector and n=18 from industry (appendix figure A1).

Participant characteristics

Figure 1 provides descriptive characteristics on participants. Overall, more than 80% of responders worked on studies of more than 100 participants, and 80% worked on phase II/III trials. A greater proportion of industry participants were working on phase I/dose finding trials compared to CTU/public sector participants (22% vs 2%). The mean number of years of experience was 12.8 (SD 8.3) (median 11.5 years, range (1-35 years)). Appendix table A1 provides further summary statistics on participant characteristics by sector and for the overall sample.

Current analysis practice

Seventy-five percent of participants reported that they present both number of participants with at least one event and number of events, 13% reported only presenting the number with at least one event, 2% stated that they only present number of events and 11% reported not presenting either of these. Other ways of presenting AE information included presenting information on overall number of events (n=2); number of patients experiencing 0, 1, 2 etc. events and number of AEs per patient

For peer review only

(n=2); duration (n=1); relatedness (n=1) and severity (n=7). Appendix table A2 provides summary statistics on current AE analysis practice by sector and for the overall sample.

Ninety percent of participants reported that they use frequencies and percentages to summarise AE data, less than 20% reported use of risk differences (16%), odds ratios (OR) (16%) or risk ratios (RR) (17%), just under a quarter reported use of incidence rate ratios (IRR) (23%) and one participant (2%) commented that they present the "*Median number (IQR)*" (appendix table A2). Several participants (n=5) commented that the summary statistic used for analysis depended on the specific study being analysed.

When comparing AE rates between treatment arms 80% of participants reported typically relying on subjective comparisons, 33% compare rates using hypothesis tests, and 22% use 95% confidence intervals (CIs) as a means to examine the null hypothesis of no difference. CTU/public sector participants reported wider use of both hypothesis tests (39% CTUs/public sector versus 17% industry) and 95% CIs (26% CTUs/public sector versus 11% industry). Fourteen percent of participants reported another means of comparison (appendix table A2), two of these related to the calculation of CIs for precision, one indicated use of a graphical summary and four comments cautioned against the use of testing e.g., "*statistical testing is rarely requested and raises multiple testing concerns*".

Just under 40% stated that they were aware of methods published specifically for AE analysis in RCTs (appendix table A3). Methods mentioned were classified into one of five groups:

(i) Bayesian approaches (n=1) e.g. *"Bayesian methods to analyse low frequency event data"* (CTU/public sector participant)

For peer review only

(ii) Modelling approaches appropriate to different data types (n=6) e.g. "*Classical Poisson/Negative Binomial/ZIP Regression for incidence rates"* (Industry participant) and *"Survival methods"* (CTU/public sector participant)

(iii) Meta-analysis (n=2) e.g. *"Meta analysis of Rare events"* (Industry participant)

(iv) Graphics (n=2) e.g. "*Graphics for biological parameters (ellipse ci)*" (CTU/public sector participant)

(v) Incidence rates (n=5) e.g. "*crude incidence rates, exposure-adjusted incidence rates, mean cumulative function (MCF)*" (CTU/public sector participant).

Participants also directed us to theoretical and applied examples in the literature (n=6).[16, 20-24] Full free text comments are reported in appendix table A4.

Only thirteen percent reported undertaking specialist AE analysis (appendix table A3), of which five participants provided details, which can be summarised as (full text comments are reported in appendix table A5):

(i) Time-to-event analysis (n=2) e.g. "*In characterising safety signals I have used Time to Event, Event rates, prevalence"* (Industry participant)

(ii) Data visualisations (n=1) e.g. *"Data visualisation (which is more or less equivalent to frequencies and percentages)"* (Industry participant)

(iii) Bayesian methods (n=1) e.g. *"Bayesian methods for sparse adverse events data meta-analysis"* (CTU/public sector participant)

(iv) Incorporating repeated events (n=1) e.g. *"For within-patient repeated events we have produced comparisons with a 2-d frequency table (arm vs # events)"* (CTU/public sector participant).

For peer review only

Of the participants who reported that they were aware of specialist AE analysis methods, we asked opinions on why such methods were not more widely used. Just over a quarter thought limited use was due to technical complexity (27%); over a third thought it could be due to trial characteristics such as unsuitability of sample sizes (36%) and the number of different AEs experienced in trials (36%); and 46% thought methods were too resource intensive and methods were not suitable for typical AE rates observed (appendix table A6).

For peer review only

Over three-quarters (77%) of participants provided further reasons for lack of use of specialist methods. Reasons were characterised into comments relating to: concerns with the suitability of methods in relation to trial characteristics and nature of AE data (n=7); opposition and a lack of understanding from clinicians (n=5); a lack of need for such methods (n=3); a desire to keep analysis consistent with historical analysis (n=3); and training and resources (n=1). Table 1 displays the participant comments attributed to each group.

Influences, barriers and concerns

The most common influences for the AE analysis performed were cited as the chief investigator's preference for simple approaches (78%), the observed AE rates (76%) and the size of the trial (73%). Over 60% of participants felt that the statistician preferred simple approaches for AE analysis (68%), and the number of different AEs experienced in a trial were influential (65%). Less than 50% of participants thought that journals (48%) or regulators (48%) preferred simple approaches but there was a notable difference by sector. A greater proportion of industry participants thought regulators

preferred simple approaches (67% versus 40%); and a greater proportion of CTU/public sector participants thought journals preferred simple approaches (56% versus 28%).

Classification of reasons given for the lack of use of specialist AE analysis methods	Participant comment
**1.Concern with the suitability of methods in relation to trial design characteristics and nature of AE data**	*"…These analyses methods may also not be appropriate if there are doubts about the robustness of AE data…"* (CTU/public sector)
	"The strongest driver is sample size and multiplicity with multiple endpoints, limiting the power of any such analysis." (CTU/public sector)
	"AEs not the primary objective of trial, Pharmaceutical companies focused not on most powerful analyses, issues around multiplicity, recurrent events, low incidence of events" (Industry)
	"…Most AE signals will not result in a statistically significant difference (due to low rates and trial size) and therefore a fear of testing exists, as statisticians we do not want to give the impression that the signal is not real as p>0.05!! Few trials are designed to specifically look at safety, the above methods are used on safety studies." (Industry)
	"…safety analyses typically lack a scientific hypotheses to direct where to look for signals." (CTU/public sector)
	"…2) Multiple testing issues: The multiplicity of AEs that may arise in a RCT makes it also not really appropriate to use statistical tests because of inflated false positive error rates resulting from multiple testings. …3) Even if 1 or 2 AEs of special interest are selected for statistical testing, detecting a statistically significant difference across treatment arms requires to power the trial and calculate the sample size accordingly." (Industry)
	"Appropriateness of methods depends on many factors including underlying distribution, prevalence of repeated events, whether participants were followed up for the same duration, etc. For example, if repeated events are rare and participants were followed up for the same duration then simple number and percentages of participants who experienced at least one event is sufficient. On the contrary, this will obscure the true picture if repeated events are prevalent and participants were follows up for varying periods. So I would say there is a range of statistical methods that are appropriate depending on the situation." (CTU/public sector)
**2. Opposition and a lack of understanding from clinicians**	*"Lack of emphasis placed by clinicians on the need for appropriate statistical methods to analyse adverse events data."* (CTU/public sector)

	"The standard approach of looking at g3+ AEs only is so accepted, there is little motivation to explore other methods. In addition, persuading clinicians to embrace other methods, can be difficult." (CTU/public sector)
	"Most medical leads on clinical trials do not understand statistical analyses and only prefer a list of AEs with their percentages to be presented" (Industry)
	"A tendency to oversimplify reporting of safety signals, to make them easier to understand to non-stats people (e.g. % are easier than incidence rates)" (Industry)
	"The template for reporting AEs is too basic. In the pharmaceutical industry the statisticians have little to no input into the trial paper" (CTU/public sector)
**3. Not deemed to be needed by statisticians**	"*Not required/ wanted.*" (CTU/public sector)
	"Don't want to report additional information in CTR" (CTU/public sector)
	"They are perhaps not used as they are no required or appropriate for that type of trial. There is no point in applying a complex method when it is not needed (eg when AEs are collected for a well established drug; when the trial is not attempting to define a safety profile)." (CTU/public sector)
**4. A desire to keep analysis consistent with historical analysis**	"*Easiness to present always the same tables"* (CTU/public sector)
	"*1) High level of standardization in reporting of results of RCTs. AE tables are pretty standard and there are requirements to meet ICH3 CSR recommendations…"* (Industry)
	"Consistency of analysis across trials in a development programme is often paramount. So, if AEs from a previous study have been analysed using a frequency/percentage approach, so would later trials." (Industry)
**5. Training and resources**	"*Training. Availability of code*." (Industry)

Table 1: Classification of participants' comments on the reasons for a lack of use of specialist methods for AE analysis

Seventy-nine percent of participants indicated that there are a lack of training opportunities to learn what methods are appropriate for AE analysis, two-thirds (66%) believed that there is a lack of awareness of appropriate methods and 58% believed there is a lack of knowledge to implement

appropriate methods. Approximately 60% of participants thought that trial characteristics including trial sample size (61%), number of different AEs experienced (61%) and AE rates (65%) were barriers when analysing AEs. Only a third (34%) of participants agreed that a lack of statistical software/code to implement appropriate methods was a barrier.

The majority of participants (84%) held the opinion that there are a lack of examples for appropriate analysis methods in the applied literature and 44% of participants thought that there are a lack of appropriate analysis methods. Over half of participants indicated that statisticians (69%), journals (60%) and chief-investigators (52%) do not give AE data the same priority as the primary efficacy outcome. Only 13% of participants believe that regulators do not prioritise AE data but nearly a quarter (24%) felt unable to comment on regulators priorities. Figure 2 provides visual summaries of influences, barriers and opinions by setting. Summary statistics by sector and for the overall sample are provided in tables A7-A9 of the appendix.

Concerns and solutions

When participants were asked to think about available methods for AE analysis the most common concern, which was held by 38% of participants was acceptability of methods to regulators. This differed substantially by sector with only 23% of CTU/public sector participants holding this belief compared to 77% of industry participants. Twenty percent of participants were concerned about the acceptability of methods to the chief investigator and journals and 32% were concerned about the robustness of methods.

For peer review only

All participants believed that guidance on appropriate AE analysis is needed, 97% thought training specifically for AE analysis is needed, and 63% thought new software or code is needed. Figure 2 provides a visual summary of concerns and solutions by setting. Summary statistics by sector and for the overall sample are provided in tables A10-A11 of the appendix. Just under a third (32%) of participants offered solutions to support change in AE analysis practices. These included suggestions regarding improved standards or calls for changes from journals, registries and regulators (n=8); development of guidance, education and engaging with the medical community (n=9); and analysis (n=3). Table 2 provides the participant comments attributed to each group.

**Classification of solutions to support a change in AE analysis practice**	**Participant comment**
**1. Improved standards or calls for changes from journals, registries and regulators**	*"Influencing journals to pay more attention to this"* (CTU)
	"…we presented incidences because they represented a fairer picture due to differential follow-up and repeated incidences per person. The reviewer and the editor said they prefer proportions and don't understand what we presented. I explained in lay terms and pushed back their request because it was flawed. This shows that Statisticians can defend a certain position and educate others even if they have their own preferences.<br><br>*Regulatory repositories/registries such as EUDRACT has a fixed format of presenting results so you have to go with what is required even though you know it's flawed in certain situation. Flexibility of such registries is very important to allow people to present both proportions and incidences where appropriate."* (CTU)
	"Asked by the authorities" (Industry)
	"Strong regulatory direction is always good for changing practices within the industry!" (Industry)
	"engaging the … regulators" (Industry)
	"*The biggest driver of a change in behaviour is usually a regulator requesting it.*" (Industry)
	"Regulators to be more demanding in analytical approaches, don't require more than summaries. That's far removed from discussions on efficacy" (Industry)

	"Would have to be able to upload the results to EUDRACT for CTIMPS." (CTU)
**2. Development of guidance, education and engaging with the medical community**	*"Best practice guidance although that would depend on trial type and phase, sample size, whether only SAEs/related AEs are being captured/important, ... particularly important to reflect on complex interventions vs CTIMP, etc"* (CTU)
	"There needs to be consensus that a change is needed. What are the issues in current AE reporting? There needs to be better guidance re collection of AE data. Can we collect it in a more robust way? We need to differentiate between examining pre-specified hypotheses and trying to identify issues we don't know about (eg in early phase trials). We need agreement re standards for different phases and types of trials (eg Phase 1 vs Phase 4, explanatory vs pragmatic, regulatory submissions vs investigator led exploratory trials on marketed products)" (CTU)
	"Published case studies" (Industry)
	"engaging the medical community …. and Better education on the pros of using proper stats methodology. If the benefits of using effective statistical analysis methods over frequencies and percentages can be demonstrated, there might be more interest" (Industry)
	"demonstration of the benefits of these methods over existing ones, and when they are appropriate" (CTU)
	"Open discussions with clinical community (e.g. open forums, etc) on alternative methods to avoid them being scared off" (Industry)
	More focus on safety analyses in the E9 addendum" (Industry)
	"Application of CONSORT harms" (CTU)
	"Evolution of standard reporting requirements in clinical trials (ICH E3, and maybe CONSORT Statement ?)" (Industry)
**3. Analysis**	*"IPD meta analysis of AEs" (CTU)*
	"In addition to 'methods' there perhaps need to be discussion about populations/datasets on which to base AE analyses." (CTU)
	"Inferential analysis based on small numbers of adverse events, but of great influence on the patient health." (Industry)

Table 2: Classification of participants' comments on solutions to support change in AE analysis practices

Thirty percent of participants raised other items not listed in the survey regarding current AE analysis practices, these covered the following themes: minimum summary information that participants would expect to be reported for AE data such as "numbers and percentages" (n=2); changes to analysis practice that could or have been made such as "use of graphical methods" (n=8); concerns about the quality and collection of AE data (n=3); and general comments and criticisms about current AE analysis and reporting practices (n=4). Table 3 provides the participant comments attributed to each theme.

In the follow-up workshop of senior statisticians (n=52 from 43 UKCRC registered CTUs) attending the UKCRC biannual statisticians' operations meeting in November 2019, participants were asked to rate the need to improve analysis practices for AE data on a scale of 0-100 (indicating low to high priority). The mean score was 66 (SD 16.2) (median 71 (range 9, 88)) (n=44).

DISCUSSION

Despite RCTs being a valuable source of data to compare rates of AEs between treatment groups and provide an opportunity to assess causality, analysis and reporting practices are often inadequate.[1-11] This survey of statisticians from the UK public and private sectors has established a more detailed picture of clinical trial statisticians' AE analysis practices and builds on our previous research which evaluated AE analysis practices reported in journal articles.[1] It has identified priorities and concerns including influences, barriers and opinions to be addressed in future work to improve AE analysis.

For peer review only

Classification of suggestions raised for AE analysis	Participant comment
**1. Minimum summary information participants would expect to be reported for AEs**	*"Different analysis approach are useful for interpretation when reporting AEs/SAEs. As a starting point, I would like to know the numbers and proportions experiencing at least one SAE by group, between group differences with uncertainty. In addition, I would like to know the incidences per group and incidence rate ratio with uncertainty. The later is not always necessary depending on the situation.."* (CTU)
	"I think in general reporting numbers and percentages is appropriate. The argument being that, if we were clinicians or patients we would want to know what is the chances of me having this event and how bad will it get, which is essentially what the frequency tables give you." (CTU)
**2. Changes that could or have been made to analysis practice**	*"No best practice guidance although revised CONSORT does help remind of importance of AE reporting"* (CTU)
	"There was a great talk at SCT 2017 on using graphical methods to summarise AEs and I have been trying to implement graphical methods to summarise the many dimensions of AE reporting as a way forward" (CTU)
	"Use of graphical methods in reporting to compare treatments ought to be standard, as per BMJ article. They are easy enough to apply…<br>*…The format of the source data, typically free text, is a pain to code into MedDRA. Methods to make this easier would be very valuable: some sort of AI machine learning maybe?...*<br>*…Meta-analysis should be very important to apply to safety data, given how under-powered individual trials may be for safety comparisons. Finding tools to automate, maybe using results entered on EudraCT might be an idea."* (CTU)
	"We have increased our use of graphics. I find benefit risk plots a very powerful way of summarising data. Allows key efficacy and safety to be displayed on one page and is a really useful summary of a drug's profile." (Industry)
	"Current practice will need to turn to methods of detecting signals as real-time data come from trials." (Industry)
	"Signal detection method" (CTU)
	"I'm interested in knowing more about risk factors of occurrence of serious or really frequent AEs of chemotherapies, beyond receiving protocol x." (Industry)

	"… not many medical leads understand statistical analysis of AEs or count or rate data and only insist on percentages and frequencies. Better methods exist but are not utilised due to lack of knowledge of PIs or medical advisors" (Industry)
**3. Concerns about the quality and collection of AE data**	*"This definitely gets overlooked. I always worry about how systematically the data have been collected too as well as the validity of lumping very different events together in the same analysis."* (CTU)
	"I think a big factor in what analysis we choose is how the data is collected. If the data is not detailed enough some only simple methods may be appropriate - this has often been my feeling when analysing our data. this may change in current/future trials as we are changing how we collect some AE data" (CTU)
	"My concerns start with the quality of AE data collected. Is it complete? Is it robust? There is recall bias, variability between centres, investigators etc. There may also be variability with respect to coding. We all have experience of stating up front what should NOT be recorded as AE, to see such things recorded multiple times. One of my major concerns is the listing of AEs each with associated p-values (obviously the CI would insist on this and not the statistician). Completely meaningless as it doesn't take into account sample size, rate, number of events within a participants, severity of event etc etc. Also of concern is the use of more complex methodologies on such data as it implies that the data are robust. I think that the simple approach is often acceptable so long as the data are presented in different ways (see Q16). The main issue is about defining what you are trying to detect from the collection of AE data. If we can do this better then perhaps additional required methodology will come." (CTU)
**4. General comments and criticisms about current AE analysis and reporting practices**	*"Somewhat arbitrary grouping of AEs. Not always clear whether numbers are subjects or events are presented in published papers."* (CTU)
	"In my 8.5 years of experience I have not seen many studies where they have spoken much about AE data analysis." (Industry)
	"People do the most powerful test for efficacy - no barrel goes unscraped - and the least powerful for safety" (CTU)
	"It can be improved!" (Industry)

Table 3: Classification of participants' general comments raised regarding AE analysis practices

Results were broadly similar across public and industry sectors with the only notable differences being the greater use of hypothesis testing and 95% CIs as a means to compare AE rates between treatment groups by CTU participants, a more predominant belief by industry participants that regulators preferred simple approaches to AE analysis, and a greater concern about acceptability of methods to regulators by industry participants. Across sectors, there was unanimous support that guidance and training on appropriate AE analysis is needed.

Survey responses indicated that 75% of statisticians produce tables with both the number of participants with at least one event and the total number of events. This is substantially higher than reported in reviews of published articles, which found between 1% and 9% reported both.[1-3] The number of total events experienced can give a better summary of impact to patients' quality-of-life but it seems this is often omitted from journal articles with reviews identifying only 6% to 7% of published articles reporting this information.[1, 4] Reported use of 95% CIs were similar to that reported in journal articles (22% compared to 20%) but reported use of hypothesis testing was lower than what was found in journal articles (32% compared to a range of 38% to 47%).[1-3] Reasons for these disparities are not known but could include journals editors requesting such analyses is undertaken to compare groups, or at the request of the chief investigator, which is supported by survey responses indicating a preference for simple approaches from both groups.

Many methods have been specifically proposed for AE analysis in RCTs and there was a moderate level of awareness of these methods (40%) but in line with our review of journal articles we found uptake to be minimal (13%).[12, 13] Whilst not directly comparable, our results are also closely aligned with the results of a survey of industry statisticians and clinical safety scientists, undertaken by Colopy and colleagues that indicated a reliance on traditional methods for descriptive statistics and frequentist approaches.[25]

For peer review only

This survey did not specifically ask participants about their use of graphics to display AE data but a similar proportion of participants indicated use of such summaries in free text comments as identified in our review of journal articles (9% vs 12%).[1] However, these figures were both substantially lower than the 37% that indicated use of static visual displays for study level AE analysis in the survey of industry statisticians.[25] This could reflect the use of more advanced graphical approaches for internal reports.

Education via training and guidance for statisticians and trialists about appropriate AE analysis could lead to improved practice and were both strongly endorsed as solutions by participants of both the survey and workshop. Guidelines such as the harms extension to CONSORT; the pharmaceutical industry standard from the Safety Planning, Evaluation and Reporting Team (SPERT); and the joint pharmaceutical/journal editor collaboration guidance on reporting of harm data in journal articles already exist and make several recommendations for analysing AEs.[15, 16, 26] However, adherence to the CONSORT Harms checklist has been shown to be poor; and whilst the impact of the Lineberry et al. guidance and the Crowe et al. guidance has not been formally evaluated, our review of AE analysis practices indicate uptake of suggestions within these guidelines such as "reporting CIs around absolute risk differences" and to "include both the number of events (per person time) and the number of patients experiencing the event" to be minimal.[1, 2, 4, 10, 11] Tutorial papers or case studies detailing examples of appropriate analysis could lead to wider adoption of such recommendations and to improvements in analysis practices, and development of such resources was highlighted as a priority by workshop participants. Whilst the acquirement of the necessary knowledge and skills to implement new methods is essential, so too is increasing awareness of good practices and alternative methods. Guidance or tutorial papers can be useful to increase knowledge, but wide dissemination and promotion to encourage use is vital if we are to improve practice.

For peer review only

A change in attitude from both statisticians and the wider research community away from doing what they have always done is also needed. Journals and regulators play a leading role in influencing good practice and could influence statisticians and trialists practice through policy change. The New England Journal of Medicine has already updated their policy to demand that evidence about both benefits and harms of treatments include point estimates and margins of error; and require no adjustment for multiplicity where significance tests are performed for harm outcomes "*Because information contained in the safety endpoints may signal problems within specific organ classes, the editors believe that the type I error rates larger than 0.05 are acceptable*."[27] A journal wide initiative to adopt existing guidelines, for example, through the mandatory submission of the CONSORT harms checklist would be one simple, initial step towards change.

Trial design and the nature of AE outcomes can also hinder the analyses performed. Unlike efficacy outcomes, which are well defined and limited in number from the outset, harm outcomes are numerous, undefined and contain additional information on severity, timing and duration, and number of occurrences, which all need to be considered. More careful consideration of harm outcomes when designing, analysing and reporting trials will help produce a more balanced view of benefits and risks.

Improved analysis could be achieved through adoption of existing or development of more appropriate methods for AE data. Several participants mentioned AE analysis approaches we believe warrant exploring including time-to-event analyses, data-visualisations and Bayesian methods. Ultimately, with the aim of helping to identify signals for ADRs enabling a clearer harm profile to be presented. This is supported by feedback obtained at the workshop and the earlier findings of

For peer review only

Colopy et al. who concluded that statisticians should help “minimize the submission of uninformative and uninterpretable reports” and thus present more informative information regarding likely drug-event relationships.[25]

Participants of both the survey and workshop raised concerns about the quality and reporting of AE data from RCTs. We agree that if AE data is not robust the analysis approach is redundant as the results will not be accurate. Therefore, procedures should be put in place at the trial design stage to mitigate problems with AE collection, including for example, development of validated methods for AE data collection and clear, standardised instructions for those involved in the detection and collection of AE data.[3, 28]

Strengths and limitations

Through support of the UKCRC CTU network and utilisation of personal contacts, we were able to achieve a high response rate for the survey. There was some level of self-selection for those recruited via the open platform and as such, there is a possibility that these participants had an increased interest in AE analysis and are not fully representative of the clinical trial community. We also did not have any information on non-responders and as such cannot characterise any potentially relevant differences that could affect the generalisability of our results. This survey provides insight and essential starting points to identify areas of focus to help support a change to improve AE analysis practice. Many of the opinions raised in the survey were echoed by the workshop attendees who represented more of a general interest group.

Conclusions

For peer review only

This research supports our earlier work identifying AE analysis practices in RCTs as sub-optimal and confirms the under use of more sophisticated AE analysis approaches. Improvements are needed and this research highlighted a unanimous call for guidance on appropriate methods for AE analysis with training to support change. In addition, further research is needed to identify the most appropriate statistical methods for AE data analysis from all those available, specifically for emerging, non-pre-defined events.

For peer review only

Acknowledgments

The authors would like to thank:

Dr Odile Sauzet for discussing the survey design in the initial stages of development.

Louise Williams (UKCRC) for her support, in particular circulating the survey to UKCRC registered CTUs and helping us achieve such a high response rate, and Professor Carrol Gamble and Professor Catherine Hewitt (UKCRC statistical operational group chairs) for supporting this project.

Alexander Schacht for inviting us on to his podcast to promote the survey and circulating to his contacts through LinkedIn.

Dr Suzie Cro, Nicholas Johnson, Anca Chis Ster, Emily Day, Fiona Reid and Professor Carrol Gamble for providing feedback on survey content and platform. Also to Dr Suzie Cro for her help in facilitating the workshop at the UKCRC biannual statisticians' operations group meeting.

COMPETING INTERESTS

None declared.

ETHICS

This study was granted ethical approval by the Imperial College Joint Research Compliance Office (ICREC reference: 19IC5067).

FUNDING

This research was supported by the NIHR grant number DRF-2017-10-131.

For peer review only

DATA SHARING STATEMENT

Survey data are available from the Zenodo data repository.

AUTHOR CONTRIBUTIONS

RP and VC conceived the idea, designed and ran the survey. RP performed the data analysis, interpreted the results and wrote the manuscript. VC interpreted the results, provided critical revision of the manuscript and supervised the project.

Figure legends

Figure 1: Participant characteristics by sector and overall

(Acronyms: CRO: Clinical Research Organisation; Pharma: Pharmaceuticals; CTUs: Clinical Trials Units)

Figure 2: Survey results by sector (a) Influences on the analysis of AEs (b) Barriers to improve AE analysis (c) Opinions on current AE analysis (d) Reasons for concern with existing methods for AE analysis (e) Potential solutions for change (improving AE analysis)

(Acronyms: CTU: Clinical Trials Unit; CI: Chief Investigator; AE: adverse event)

For peer review only

REFERENCES

1. Phillips R, Hazell L, Sauzet O, et al. Analysis and reporting of adverse events in randomised controlled trials: a review. *BMJ Open* 2019; 9: e024537. 2019/03/04. DOI: 10.1136/bmjopen-2018-024537.

2. Pitrou I, Boutron I, Ahmad N, et al. Reporting of safety results in published reports of randomized controlled trials. *Archives of Internal Medicine* 2009; 169: 1756-1761. DOI: 10.1001/archinternmed.2009.306.

3. Cornelius VR, Sauzet O, Williams JE, et al. Adverse event reporting in randomised controlled trials of neuropathic pain: Considerations for future practice. *PAIN* 2013; 154: 213-220. DOI: 10.1016/j.pain.2012.08.012.

4. Hum SW, Golder S and Shaikh N. Inadequate harms reporting in randomized control trials of antibiotics for pediatric acute otitis media: a systematic review. *Drug Safety* 2018 May 08. DOI: 10.1007/s40264-018-0680-0.

5. Ioannidis JPA and Contopoulos-Ioannidis DG. Reporting of safety data from randomised trials. *The Lancet* 1998; 352: 1752-1753. DOI: 10.1016/S0140-6736(05)79825-1.

6. Edwards JE, McQuay HJ, Moore RA, et al. Reporting of Adverse Effects in Clinical Trials Should Be Improved. *Journal of Pain and Symptom Management* 1999; 18: 427-437. DOI: 10.1016/S0885-3924(99)00093-7.

7. Ioannidis JA and Lau J. Completeness of safety reporting in randomized trials: An evaluation of 7 medical areas. *JAMA* 2001; 285: 437-443. DOI: 10.1001/jama.285.4.437.

8. Maggi CB, Griebeler IH and Dal Pizzol Tda S. Information on adverse events in randomised clinical trials assessing drug interventions published in four medical journals with high impact factors. *Int J Risk Saf Med* 2014; 26: 9-22. 2014/05/07. DOI: 10.3233/JRS-140609.

9. Smith SM, Wang AT, Katz NP, et al. Adverse event assessment, analysis, and reporting in recent published analgesic clinical trials: ACTTION systematic review and recommendations. *PAIN* 2013; 154: 997-1008. DOI: 10.1016/j.pain.2013.03.003.

For peer review only

10. Peron J, Maillet D, Gan HK, et al. Adherence to CONSORT adverse event reporting guidelines in randomized clinical trials evaluating systemic cancer therapy: a systematic review. *J Clin Oncol* 2013; 31: 3957-3963. 2013/09/26. DOI: 10.1200/JCO.2013.49.3981.

11. Favier R and Crépin S. The reporting of harms in publications on randomized controlled trials funded by the "Programme Hospitalier de Recherche Clinique," a French academic funding scheme. *Clinical Trials* 2018; 0: 1740774518760565. DOI: 10.1177/1740774518760565.

12. Phillips R, Sauzet O and Cornelius V. Statistical methods for the analysis of adverse event data in randomised controlled trials: a review of available methods. *(Unpublished)*.

13. Phillips R, Cornelius V and Sauzet O. An evaluation and application of statistical methods designed to analyse adverse event data in RCTs. *Trials Conference: 5th International Clinical Trials Methodology Conference, ICTMC 2019 United Kingdom* 2019; 20.

14. Chuang-Stein C and Xia HA. The practice of pre-marketing safety assessment in drug development. *Journal of Biopharmaceutical Statistics* 2013; 23: 3-25. Review. DOI: 10.1080/10543406.2013.736805.

15. Ioannidis JA, Evans SW, Gøtzsche PC, et al. Better reporting of harms in randomized trials: An extension of the consort statement. *Annals of Internal Medicine* 2004; 141: 781-788. DOI: 10.7326/0003-4819-141-10-200411160-00009.

16. Lineberry N, Berlin JA, Mansi B, et al. Recommendations to improve adverse event reporting in clinical trial publications: A joint pharmaceutical industry/journal editor perspective. *BMJ (Online)* 2016; 355: i5078.

17. Love SB, Brown S, Weir CJ, et al. Embracing model-based designs for dose-finding trials. *British journal of cancer* 2017; 117: 332-339. 06/29. DOI: 10.1038/bjc.2017.186.

18. Dimairo M, Julious SA, Todd S, et al. Cross-sector surveys assessing perceptions of key stakeholders towards barriers, concerns and facilitators to the appropriate use of adaptive designs in confirmatory trials. *Trials* 2015; 16: 585-585. DOI: 10.1186/s13063-015-1119-x.

For peer review only

19. Kelley K, Clark B, Brown V, et al. Good practice in the conduct and reporting of survey research. *International Journal for Quality in Health Care* 2003; 15: 261-266. DOI: 10.1093/intqhc/mzg031.

20. Proctor T and Schumacher M. Analysing adverse events by time-to-event models: the CLEOPATRA study. *Pharmaceutical Statistics* 2016; 15: 306-314. DOI: 10.1002/pst.1758.

21. Southworth H. Detecting outliers in multivariate laboratory data. *Journal of Biopharmaceutical Statistics* 2008; 18: 1178-1183.

22. Allignol A, Beyersmann J and Schmoor C. Statistical issues in the analysis of adverse events in time-to-event data. *Pharmaceutical Statistics* 2016; 15: 297-305.

23. Special Issue:Analysis of Adverse Event Data. *Pharmaceutical Statistics* 2016; 15: 287-379.

24. Unkel S, Amiri M, Benda N, et al. On estimands and the analysis of adverse events in the presence of varying follow-up times within the benefit assessment of therapies. *Pharmaceutical Statistics* 2019; 18: 166-183. DOI: 10.1002/pst.1915.

25. Colopy MW, Gordon R, Ahmad F, et al. Statistical Practices of Safety Monitoring: An Industry Survey. *Therapeutic Innovation & Regulatory Science* 2019; 53: 293-300. DOI: 10.1177/2168479018779973.

26. Crowe BJ, Xia HA, Berlin JA, et al. Recommendations for safety planning, data collection, evaluation and reporting during drug, biologic and vaccine development: a report of the safety planning, evaluation, and reporting team. *Clinical Trials* 2009; 6: 430-440. DOI: 10.1177/1740774509344101.

27. Harrington D, D'Agostino RB, Gatsonis C, et al. New Guidelines for Statistical Reporting in the Journal. *New England Journal of Medicine* 2019; 381: 285-286. DOI: 10.1056/NEJMe1906559.

28. Stephens MD, Talbot JC and Routledge PA. *The Detection of New Adverse Reactions*. 4 ed. London: Macmillan Reference 1998.

For peer review only

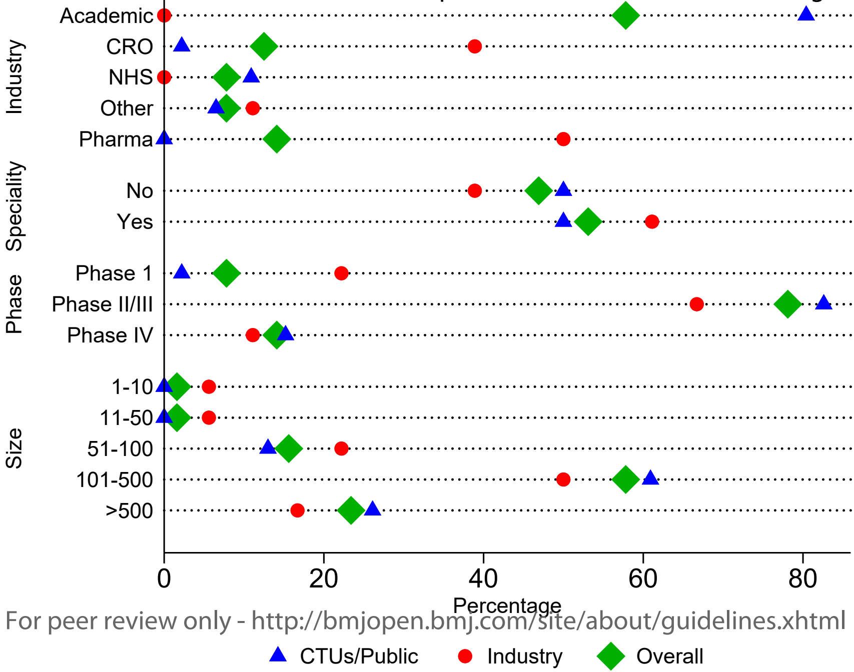

For peer review only - http://bmjopen.bmj.com/site/about/guidelines.xhtml

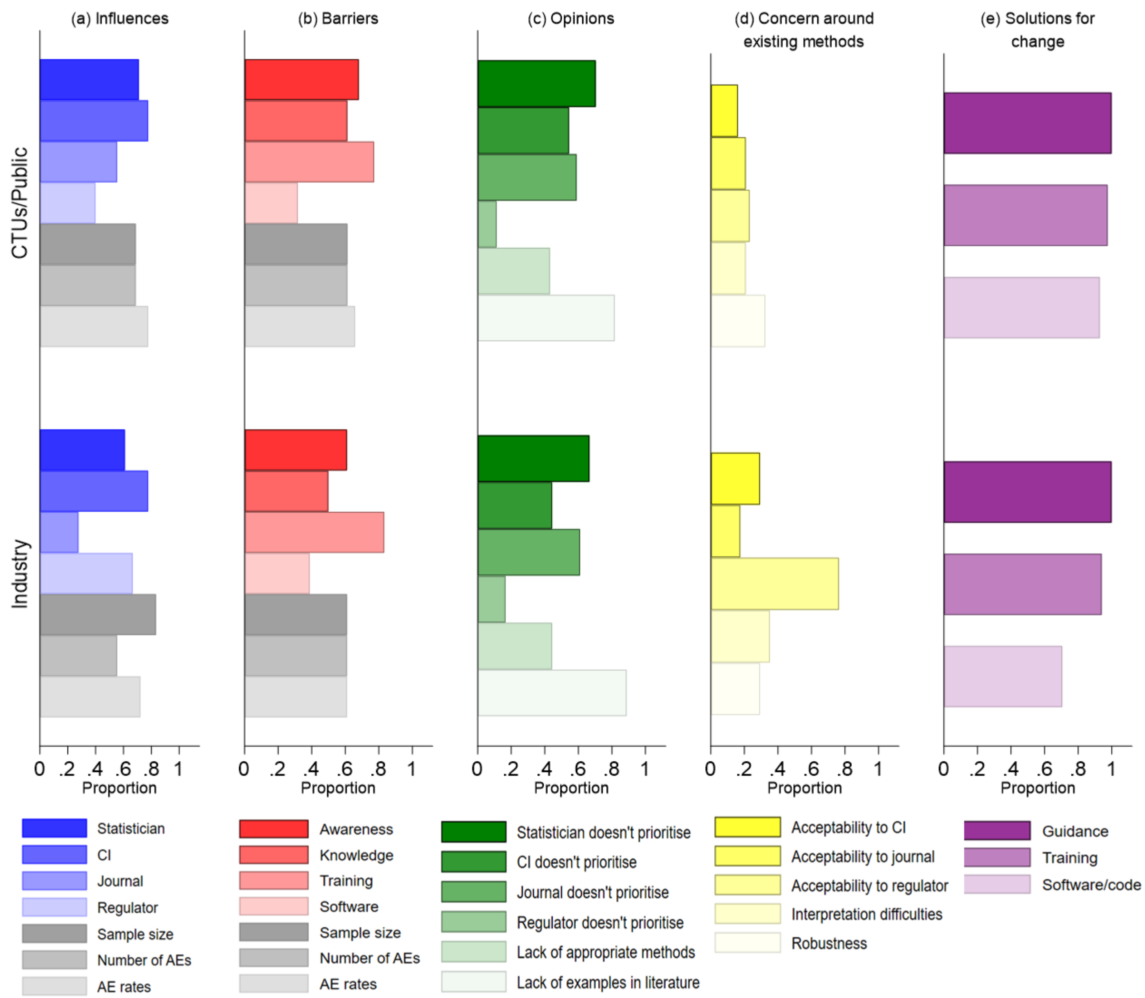
(a) Influences
(b) Barriers
(c) Opinions
(d) Concern around existing methods
(e) Solutions for change
CTUs/Public
Industry
0 .2 .4 .6 .8 1
Proportion
Statistician
CI
Journal
Regulator
Sample size
Number of AEs
AE rates
Awareness
Knowledge
Training
Software
Sample size
Number of AEs
AE rates
Statistician doesn't prioritise
CI doesn't prioritise
Journal doesn't prioritise
Regulator doesn't prioritise
Lack of appropriate methods
Lack of examples in literature
Acceptability to CI
Acceptability to journal
Acceptability to regulator
Interpretation difficulties
Robustness
Guidance
Training
Software/code

Appendix

Item 1: Survey questions

Study Title: Statisticians survey on statistical methods for adverse event data analysis in randomised controlled trials

This survey pertains to the final analysis of AEs reported or screened for in clinical trials. Not predefined specific single safety outcomes of interest or interim analyses.

Number	Question	Response options				
1	How long have you worked as a clinical trial statistician? (Please specify the number of years)					
2	Do you work for:	Academic institution	NHS trust	Pharmaceutical company	Clinical Research Organisation	Other (please specify)
3	Is there a clinical area you predominantly work on? If yes, please specify	No	Yes			
4	What is the typical size of the trials you work on?	1-10	11-50	51-100	101-500	>500
5	What is the typical phase of the trials you work on?	Phase I/Dose-finding	Phase II/III	Phase IV		

Before you proceed we thought it would be helpful for you to know about our recent findings.

We undertook a systematic review of RCT journal reports and found that trials typically report AE data using frequencies (94%) and percentages (87%). They often ignore repeated events (84%) and 47% undertake hypothesis tests despite a lack of power. There is also a common practice to categorise continuous clinical and laboratory outcomes and present as frequencies and percentages (59%). A small proportion (12%) incorporated graphics into the AE analysis.

	Thinking about analysis methods for AEs:					
6	How often would you say the following influences the analysis performed?					
i	Statistician prefers simple approaches e.g. tables of frequencies and percentages	Always	Often	Not very often	Never	Don't know

For peer review only - http://bmjopen.bmj.com/site/about/guidelines.xhtml

Appendix

	ii	Chief investigator prefers simple approaches e.g. tables of frequencies and percentages	Always	Often	Not very often	Never	Don't know
	iii	Journal prefers simple approaches e.g. tables of frequencies and percentages	Always	Often	Not very often	Never	Don't know
	iv	Regulator prefers simple approaches e.g. tables of frequencies and percentages	Always	Often	Not very often	Never	Don't know
	v	Trial sample size	Always	Often	Not very often	Never	Don't know
	vi	The number of different AEs experienced across the trial	Always	Often	Not very often	Never	Don't know
	vii	AE rates	Always	Often	Not very often	Never	Don't know
		Thinking about AE analysis you typically perform.					
7		In your experience the following is a barrier when analysing AEs:					
	i	Lack of awareness of appropriate methods	Strongly agree	Agree	Disagree	Strongly disagree	Don't know
	ii	Lack of knowledge to implement appropriate methods	Strongly agree	Agree	Disagree	Strongly disagree	Don't know
	iii	Lack of training opportunities to learn what methods are appropriate	Strongly agree	Agree	Disagree	Strongly disagree	Don't know
	iv	Lack of statistical software/code to implement appropriate methods	Strongly agree	Agree	Disagree	Strongly disagree	Don't know
	iv	Trial sample size	Strongly agree	Agree	Disagree	Strongly disagree	Don't know
	v	The number of different AEs experienced across the trial	Strongly agree	Agree	Disagree	Strongly disagree	Don't know
	vi	AE rates	Strongly agree	Agree	Disagree	Strongly disagree	Don't know
		Thinking about AE analysis.					
8		In your opinion:					
	i	Statisticians don't give AE data the same priority as the primary efficacy outcome	Strongly agree	Agree	Disagree	Strongly disagree	Don't know

For peer review only

For peer review only - http://bmjopen.bmj.com/site/about/guidelines.xhtml

Appendix

	ii	Chief investigators don't give AE data the same priority as the primary efficacy outcome	Strongly agree	Agree	Disagree	Strongly disagree	Don't know
	iii	Journals don't give AE data the same priority as the primary efficacy outcome	Strongly agree	Agree	Disagree	Strongly disagree	Don't know
	iv	Regulators don't give AE data the same priority as the primary efficacy outcome	Strongly agree	Agree	Disagree	Strongly disagree	Don't know
	v	There are a lack of appropriate analysis methods	Strongly agree	Agree	Disagree	Strongly disagree	Don't know
	vi	There are a lack of examples of the use of appropriate analysis methods in the applied literature	Strongly agree	Agree	Disagree	Strongly disagree	Don't know
9		Are you aware of any published methods specifically to analyse AEs? If yes, please specify	Yes	No	Don't know		
10		If answer is 'yes' to question 9 In your opinion why are those methods not being more widely used:					
	i	Available methods are technically too complex	Strongly agree	Agree	Disagree	Strongly disagree	Don't know
	ii	Available methods are too resource intensive	Strongly agree	Agree	Disagree	Strongly disagree	Don't know
	iii	Available methods are not suitable for typical trial sample sizes	Strongly agree	Agree	Disagree	Strongly disagree	Don't know
	iv	Available methods are not suitable for the number of different AEs typically experienced across a trial	Strongly agree	Agree	Disagree	Strongly disagree	Don't know
	v	Available methods are not suitable for typical AE rates observed	Strongly agree	Agree	Disagree	Strongly disagree	Don't know
11		Are there any reasons other than those mention above why those methods are not being more widely used? If yes, please specify	Yes	No			

For peer review only - http://bmjopen.bmj.com/site/about/guidelines.xhtml

Appendix

		Thinking about available methods for AE analysis					
12		How concerned are you about the following:					
	i	Difficulties in interpreting the results/output	Not at all	Slightly concerned	Somewhat concerned	Moderately concerned	Extremely concerned
	ii	Robustness of methods	Not at all	Slightly concerned	Somewhat concerned	Moderately concerned	Extremely concerned
	iii	Acceptability of methods to chief investigator	Not at all	Slightly concerned	Somewhat concerned	Moderately concerned	Extremely concerned
	iv	Acceptability of methods to journal	Not at all	Slightly concerned	Somewhat concerned	Moderately concerned	Extremely concerned
	v	Acceptability of methods to regulator	Not at all	Slightly concerned	Somewhat concerned	Moderately concerned	Extremely concerned
13		Do you have any other thoughts about current practice for AE analysis?	Yes	No			
		If yes, please specify					
14		To what extent do you agree that the following would support a change in AE analysis practice					
	i	Software/code development is needed	Strongly agree	Agree	Disagree	Strongly disagree	Don't know
	ii	Training specifically for AE analysis is needed	Strongly agree	Agree	Disagree	Strongly disagree	Don't know
	iii	Guidance on appropriate AE analysis is needed e.g. case studies, tutorials within open access journals	Strongly agree	Agree	Disagree	Strongly disagree	Don't know
15		Are there any other solutions in addition to those above that would support a change in AE analysis practice?	Yes	No			
		If yes, please specify					
16		When analysing AEs do you present (please select all that apply):					

For peer review only - http://bmjopen.bmj.com/site/about/guidelines.xhtml

Appendix

	i	Number of participants with at least one event	Yes	No
	ii	Number of events	Yes	No
	iii	Other	Yes	No
		If yes, please specify		
17		When analysing AEs which summary statistic would you typically use (please select all that apply)		
	i	Frequency	Yes	No
	ii	Percentage	Yes	No
	iii	Risk difference	Yes	No
	iv	Odds ratio	Yes	No
	v	Risk ratio	Yes	No
	vi	Incidence rate ratio	Yes	No
	vii	Other	Yes	No
		If yes, please specify		
18		In your experience how are AE rates typically compared between treatment groups (please select all that apply)		
	i	Subjective comparison	Yes	No
	ii	Exclusion of null through 95% confidence interval	Yes	No
	iii	Hypothesis test/p-value	Yes	No
	iv	Other	Yes	No
		If yes, please specify		
19		Have you undertaken any specialist AE analysis not mentioned in your previous responses? Please explain your answer. If ‘yes’, please include details of the method(s) used for the analysis performed	Yes	No

For peer review only - http://bmjopen.bmj.com/site/about/guidelines.xhtml

Appendix

Figure A1: Flow diagram of participation

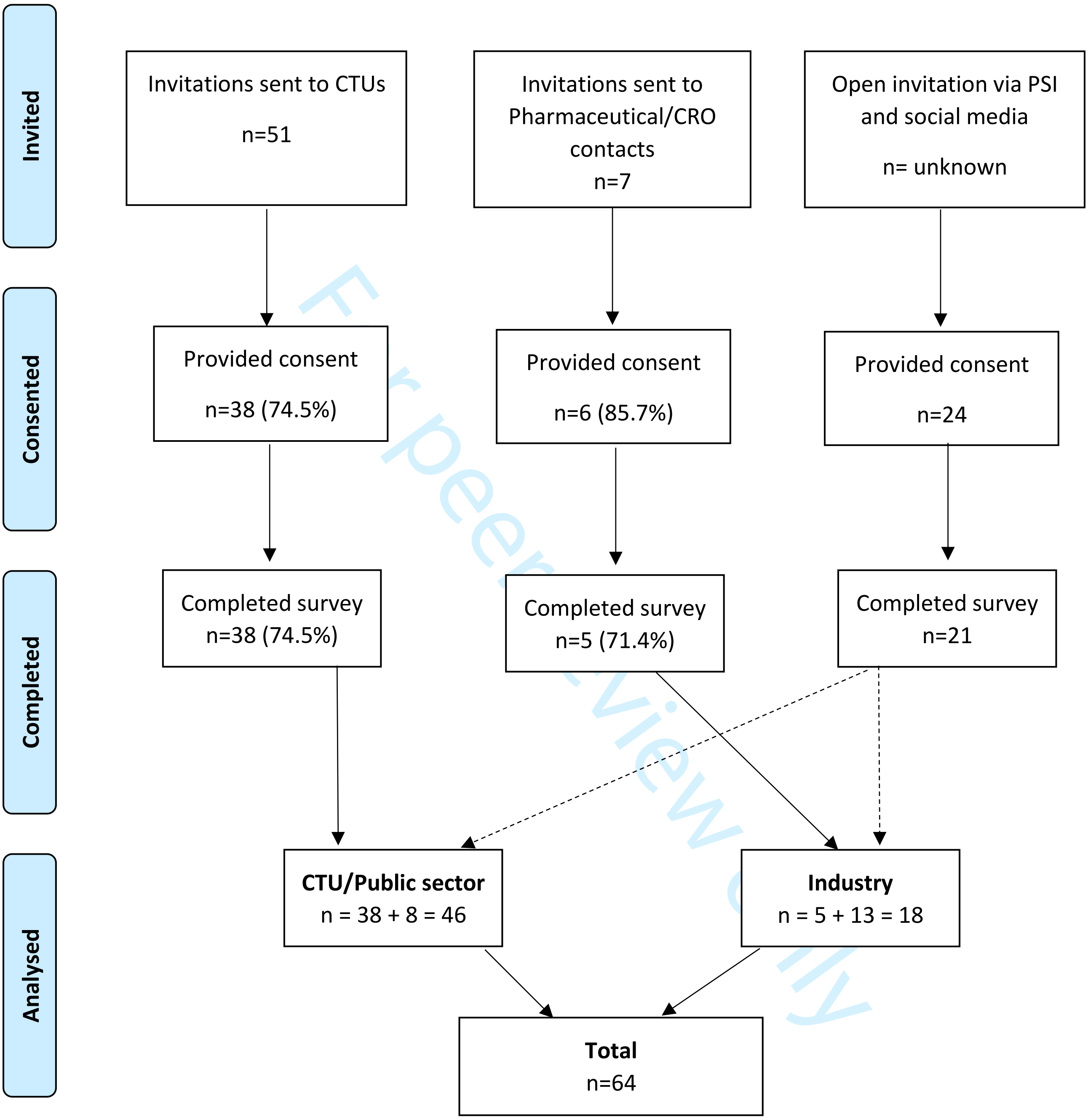

For peer review only - http://bmjopen.bmj.com/site/about/guidelines.xhtml

Appendix

Table A1: Participant characteristics by sector and overall

		CTU/Public (N=46)		Industry (N=18)		Overall (N=64)	
**Characteristics**		**n/N**	**%**	**n/N**	**%**	**n/N**	**%**
**Typical trial size**	1-10	0/46	0.0%	1/18	5.6%	1/64	1.6%
	11-50	0/46	0.0%	1/18	5.6%	1/64	1.6%
	51-100	6/46	13.0%	4/18	22.2%	10/64	15.6%
	101-500	28/46	60.9%	9/18	50.0%	37/64	57.8%
	>500	12/46	26.1%	3/18	16.7%	15/64	23.4%
**Work setting**	Academic institution	38/46	82.6%	0/18	0.0%	38/64	59.4%
	CRO	1/46	2.2%	7/18	38.9%	8/64	12.5%
	NHS trust	5/46	10.9%	0/18	0.0%	5/64	7.8%
	Pharmaceutical	0/46	0.0%	9/18	50.0%	9/64	14.1%
	Other	2/46	4.3%	2/18	11.1%	4/64	6.3%
**Speciality**	No	23/46	50.0%	7/18	38.9%	30/64	46.9%
	Yes	23/46	50.0%	11/18	61.1%	34/64	53.1%
**Typical trial phase**	Phase I/Dose-finding	1/46	2.2%	4/18	22.2%	5/64	7.8%
	Phase II/III	38/46	82.6%	12/18	66.7%	50/64	78.1%
	Phase IV	7/46	15.2%	2/18	11.1%	9/64	14.1%
**Years of experience**	Mean (SD)	12.0	(7.2)	14.7	(10.7)	12.8	(8.3)
	Median (min, max)	12.0	(1, 30)	15.5	(1, 35)	11.5	(1, 35)

Acronyms: CTU: Clinical Trial Unit; CRO: Clinical Research Organisation; SD: standard deviation; min: minimum; max: maximum

For peer review only - http://bmjopen.bmj.com/site/about/guidelines.xhtml

Appendix

Table A2: Adverse event (AE) information typically presented by sector and overall

	CTU/Public (N=46)		Industry (N=18)		Overall (N=64)	
**Information presented**	n/N	%	n/N	%	n/N	%
Number of participants with at least one event	4/46	8.7%	4/18	22.2%	8/64	12.5%
Number of events	1/46	2.1%	0/18	0.0%	1/64	1.6%
Both of the above	36/46	78.3%	12/18	66.7%	48/64	75.0%
None of the above	5/46	10.9%	2/18	11.1%	7/64	10.9%
**Summary statistic**						
Frequencies	42/46	91.3%	16/18	88.9%	58/64	90.6%
Percentages	43/46	93.5%	14/18	77.8%	57/64	89.1%
Risk difference	5/46	10.9%	5/18	27.8%	10/64	15.6%
Odds ratio	7/46	15.2%	3/18	16.7%	10/64	15.6%
Risk ratio	6/46	13.0%	5/18	27.8%	11/64	17.2%
Incidence rate ratio*	8/46	17.4%	7/18	38.9%	15/64	23.4%
Other	6/46	13.0%	4/18	22.2%	10/64	15.6%
**AE comparison**						
Subjective comparison	36/46	78.3%	15/18	83.3%	51/64	79.7%
Exclusion of null through 95% confidence interval	12/46	26.1%	2/18	11.1%	14/64	21.9%
Hypothesis test/p-value	18/46	39.1%	3/18	16.7%	21/64	32.8%
Other	4/46	8.7%	5/18	27.8%	9/64	14.1%

Acronyms: CTU: Clinical Trial Unit; AE: adverse event

*Incorporates free text comments that described summaries synonymous with incidence rate ratios

For peer review only

Appendix

Table A3: Specialist adverse event (AE) analysis

		CTU/Public (N=43)		Industry (N=17)		Overall (N=60)	
		n/N	%	n/N	%	n/N	%
Awareness of any published methods specifically to analyse AEs	Don't know	8/44	18.2%	1/17	5.9%	9/61	14.8%
	No	25/44	56.8%	4/17	23.5%	29/61	47.5%
	Yes	11/44	25.0%	12/17	70.6%	23/61	37.7%
Undertaken any specialist AE analysis not mentioned in your previous response	No	38/43	88.4%	14/17	82.4%	52/60	86.7%
	Yes	5/43	11.6%	3/17	17.6%	8/60	13.3%

Acronyms: CTU: Clinical Trial Unit; AE: adverse event

For peer review only

Appendix

Table A4: Free text comments regarding methods participants are aware of specifically for adverse event (AE) analysis

**Bayesian approaches:**
"Bayesian methods to analyse low frequency event data."
**Modelling approaches:**
"I don't think there is anything special about AEs/SAEs that require special methods. Statistical methods for the analysis of events (yes/no) or repeated events accounted for differential follow-up or/and overdispersion already exist in statistical literature (e.g., poisson or negative binomial regression model). of course, it depends on the underlying distribution"
"Classical Poisson/Negative Binomial/ZIP Regression for incidence rates"
"Extreme Value methods"
"…,survival analysis for comparison of treatment and for time to specific event"
"Survival methods"
"GEE"
**Meta-analysis:**
"…examples of meta analyses to appropriately analyse AE data"
" Meta analysis of Rare events"
**Graphics:**
"Cumulative incidence plots, life table plots, and prevalence plots. Many methods not specific to analysis of AEs"
"Graphics for biological parameters (ellipse ci)"
**Incidence rate:**
"crude incidence rates, exposure-adjusted incidence rates, mean cumulative function (MCF)"
"Rate analyses,…"
"Cumulative incidence plots, life table plots, and prevalence plots. Many methods not specific to analysis of AEs"
"Incidence rates and confidence intervals (in person-years). Time to onset."
"Rate ratio,…"
**Theoretical and applied examples:**
" CLEOPATRA Study Repeated Measures (i.e. not just counting first event)"
"Various methods published by Harry Southworth. These are predominantly useful for pharma trials rather than Phase 4 trials unit trials."
"Volume15, Issue4 Special Issue: Analysis of Adverse Event Data July/August 2016 Pages 297-305"
"http://dx.doi.org/10.1136/bmj.i5078"
"https://onlinelibrary.wiley.com/toc/15391612/2016/15/4"
"possible use of estimands to analyse AEs (for example https://arxiv.org/abs/1805.01834)"
**Other comments:**
"Not meaningfully within an early phase setting, because of sample size. Monitoring based approaches are becoming used and machine learning based methods are available."
"AE tables and summary"
"The statistical literature is awash with methods"
"zz"

For peer review only

For peer review only - http://bmjopen.bmj.com/site/about/guidelines.xhtml

Appendix

Table A5: Free text comments regarding participants' use of specialist methods for adverse event (AE) analysis

"In characterising safety signals I have used Time to Event, Event rates, prevalence."
"Time-to-event analyses; exposure-adjusted AE rates"
"Data visualisation (which is more or less equivalent to frequencies and percentages)"
"Bayesian methods for sparse adverse events data meta-analysis"
"For within-patient repeated events we have produced comparisons with a 2-d frequency table (arm vs # events)"
"Not sure I understood what is meant by specialist AE analysis. I used various statistical methods depending on the situation."
"Safety analysis in phase III cancer clinical trial"

For peer review

Table A6: Reasons specialist adverse event (AE) methods are not used (of participants aware of such methods)

**Reasons for unsuitability. Available methods are:**		**CTU/Public (N=11)**		**Industry (N=12)**		**Overall (N=23)**	
		**n/N**	**%**	**n/N**	**%**	**n/N**	**%**
Technically too complex	Strongly disagree/disagree	8/10	80.0%	6/12	50.0%	14/22	63.6%
	Agree/strongly agree	1/10	10.0%	5/12	41.7%	6/22	27.3%
	Don't know	1/10	10.0%	1/12	8.3%	2/22	9.1%
Too resource intensive	Strongly disagree/disagree	5/10	50.0%	7/12	58.3%	12/22	54.5%
	Agree/strongly agree	5/10	50.0%	5/12	41.7%	10/22	45.5%
Not suitable for typical trial sample sizes	Strongly disagree/disagree	6/10	60.0%	4/12	33.3%	10/22	45.5%
	Agree/strongly agree	3/10	30.0%	5/12	41.7%	8/22	36.4%
	Don't know	1/10	10.0%	3/12	25.0%	4/22	18.2%
Not suitable for the number of different AEs typically experienced across a trial	Strongly disagree/disagree	7/10	70.0%	5/12	41.7%	12/22	54.5%
	Agree/strongly agree	2/10	20.0%	6/12	50.0%	8/22	36.4%
	Don't know	1/10	10.0%	1/12	8.3%	2/22	9.1%
Not suitable for typical AE rates observed	Strongly disagree/disagree	7/10	70.0%	5/12	41.7%	12/22	54.5%
	Agree/strongly agree	3/10	30.0%	7/12	58.3%	10/22	45.5%
Other reasons why those methods are not used	Don't know	1/10	10.0%	1/12	8.3%	2/22	9.1%
	No	0/10	0.0%	3/12	25.0%	3/22	13.6%
	Yes	9/10	90.0%	8/12	66.7%	17/22	77.3%

Acronyms: CTU: Clinical Trial Unit; AE: adverse event

For peer review only - http://bmjopen.bmj.com/site/about/guidelines.xhtml

Appendix

Table A7: Influences the analysis performed

		CTU/Public (N=45)		Industry (N=18)		Overall (N=63)	
**Influence**		**n/N**	**%**	**n/N**	**%**	**n/N**	**%**
**Statistician prefers simple approaches e.g. tables of frequencies and percentages**	Never/Not very often	13/45	28.9%	7/18	38.9%	20/63	31.7%
	Often/Always	32/45	71.1%	11/18	61.1%	43/63	68.3%
**Chief investigator prefers simple approaches e.g. tables of frequencies and percentages**	Never/Not very often	9/45	20.0%	2/18	11.1%	11/63	17.5%
	Often/Always	35/45	77.8%	14/18	77.8%	49/63	77.8%
	Don't know	1/45	2.2%	2/18	11.1%	3/63	4.8%
**Journal prefers simple approaches e.g. tables of frequencies and percentages**	Never/Not very often	12/45	26.7%	7/18	38.9%	19/63	30.2%
	Often/Always	25/45	55.6%	5/18	27.8%	30/63	47.6%
	Don't know	8/45	17.8%	6/18	33.3%	14/63	22.2%
**Regulator prefers simple approaches e.g. tables of frequencies and percentages**	Never/Not very often	9/45	20.0%	4/18	22.2%	13/63	20.6%
	Often/Always	18/45	40.0%	12/18	66.7%	30/63	47.6%
	Don't know	18/45	40.0%	2/18	11.1%	20/63	31.7%
**Trial sample size**	Never/Not very often	12/45	26.7%	2/18	11.1%	14/63	22.2%
	Often/Always	31/45	68.9%	15/18	83.3%	46/63	73.0%
	Don't know	2/45	4.4%	1/18	5.6%	3/63	4.8%
**The number of different AEs experienced across the trial**	Never/Not very often	13/45	28.9%	7/18	38.9%	20/63	31.7%
	Often/Always	31/45	68.9%	10/18	55.6%	41/63	65.1%
	Don't know	1/45	2.2%	1/18	5.6%	2/63	3.2%
**AE rates**	Never/Not very often	9/45	20.0%	4/18	22.2%	13/63	20.6%
	Often/Always	35/45	77.8%	13/18	72.2%	48/63	76.2%
	Don't know	1/45	2.2%	1/18	5.6%	2/63	3.2%

Acronyms: CTU: Clinical Trial Unit; AE: adverse event

For peer review only

Appendix

Table A8: Barriers when analysing adverse events (AEs)

		CTU/Public (N=44)		Industry (N=18)		Overall (N=62)	
**Barriers**		**n/N**	**%**	**n/N**	**%**	**n/N**	**%**
Lack of awareness of appropriate methods	Strongly disagree/disagree	11/44	25.0%	7/18	38.9%	18/62	29.0%
	Agree/ Strongly agree	30/44	68.2%	11/18	61.1%	41/62	66.1%
	Don't know	3/44	6.8%	0/18	0.0%	3/62	4.8%
Lack of knowledge to implement appropriate methods	Strongly disagree/disagree	15/44	34.1%	8/18	44.4%	23/62	37.1%
	Agree/ Strongly agree	27/44	61.4%	9/18	50.0%	36/62	58.1%
	Don't know	2/44	4.5%	1/18	5.6%	3/62	4.8%
Lack of training opportunities to learn what methods are appropriate	Strongly disagree/disagree	7/44	15.9%	3/18	16.7%	10/62	16.1%
	Agree/ Strongly agree	34/44	77.3%	15/18	83.3%	49/62	79.0%
	Don't know	3/44	6.8%	0/18	0.0%	3/62	4.8%
Lack of statistical software/code to implement appropriate methods	Strongly disagree/disagree	21/44	47.7%	11/18	61.1%	32/62	51.6%
	Agree/ Strongly agree	14/44	31.8%	7/18	38.9%	21/62	33.9%
	Don't know	9/44	20.5%	0/18	0.0%	9/62	14.5%
Trial sample size	Strongly disagree/disagree	13/44	29.5%	7/18	38.9%	20/62	32.3%
	Agree/ Strongly agree	27/44	61.4%	11/18	61.1%	38/62	61.3%
	Don't know	4/44	9.1%	0/18	0.0%	4/62	6.5%
The number of different AEs experienced across the trial	Strongly disagree/disagree	15/44	34.1%	7/18	38.9%	22/62	35.5%
	Agree/ Strongly agree	27/44	61.4%	11/18	61.1%	38/62	61.3%
	Don't know	2/44	4.5%	0/18	0.0%	2/62	3.2%
AE rates	Strongly disagree/disagree	14/44	31.8%	7/18	38.9%	21/62	33.9%
	Agree/ Strongly agree	29/44	65.9%	11/18	61.1%	40/62	64.5%
	Don't know	1/44	2.3%	0/18	0.0%	1/62	1.6%

Acronyms: CTU: Clinical Trial Unit; AE: adverse event

For peer review only

For peer review only - http://bmjopen.bmj.com/site/about/guidelines.xhtml

Appendix

Table A9: Opinions on adverse event (AE) analysis

		CTU/Public (N=44)		Industry (N=18)		Overall (N=62)	
**Opinions**		**n/N**	**%**	**n/N**	**%**	**n/N**	**%**
Statisticians don't give AE data the same priority as the primary efficacy outcome	Strongly disagree/disagree	13/44	29.5%	6/18	33.3%	19/62	30.6%
	Agree/strongly agree	31/44	70.5%	12/18	66.7%	43/62	69.4%
Chief investigators don't give AE data the same priority as the primary efficacy outcome	Strongly disagree/disagree	20/44	45.5%	7/18	38.9%	27/62	43.5%
	Agree/strongly agree	24/44	54.5%	8/18	44.4%	32/62	51.6%
	Don't know	0/44	0.0%	3/18	16.7%	3/62	4.8%
Journals don't give AE data the same priority as the primary efficacy outcome	Strongly disagree/disagree	12/44	27.3%	4/18	22.2%	16/62	25.8%
	Agree/strongly agree	26/44	59.1%	11/18	61.1%	37/62	59.7%
	Don't know	6/44	13.6%	3/18	16.7%	9/62	14.5%
Regulators don't give AE data the same priority as the primary efficacy outcome	Strongly disagree/disagree	25/44	56.8%	14/18	77.8%	39/62	62.9%
	Agree/strongly agree	5/44	11.4%	3/18	16.7%	8/62	12.9%
	Don't know	14/44	31.8%	1/18	5.6%	15/62	24.2%
There are a lack of appropriate analysis methods	Strongly disagree/disagree	15/44	34.1%	8/18	44.4%	23/62	37.1%
	Agree/strongly agree	19/44	43.2%	8/18	44.4%	27/62	43.5%
	Don't know	10/44	22.7%	2/18	11.1%	12/62	19.4%
There are a lack of examples of the use of appropriate analysis methods in the applied literature	Strongly disagree/disagree	5/44	11.4%	1/18	5.6%	6/62	9.7%
	Agree/strongly agree	36/44	81.8%	16/18	88.9%	52/62	83.9%
	Don't know	3/44	6.8%	1/18	5.6%	4/62	6.5%

Acronyms: CTU: Clinical Trial Unit; AE: adverse event

For peer review only

Appendix

Table A10: Concerns about available methods for adverse event (AE) analysis

		CTU/Public (N=43)		Industry (N=17)		Overall (N=60)	
**Concerns**		**n/N**	**%**	**n/N**	**%**	**n/N**	**%**
Difficulties in interpreting the results/output	Not at all to somewhat concerned	34/43	79.1%	11/17	64.7%	45/60	75.0%
	Moderately to extremely concerned	9/43	20.9%	6/17	35.3%	15/60	25.0%
Robustness of methods	Not at all to somewhat concerned	29/43	67.4%	12/17	70.6%	41/60	68.3%
	Moderately to extremely concerned	14/43	32.6%	5/17	29.4%	19/60	31.7%
Acceptability of methods to chief investigator	Not at all to somewhat concerned	36/43	83.7%	12/17	70.6%	48/60	80.0%
	Moderately to extremely concerned	7/43	16.3%	5/17	29.4%	12/60	20.0%
Acceptability of methods to journal	Not at all to somewhat concerned	34/43	79.1%	14/17	82.4%	48/60	80.0%
	Moderately to extremely concerned	9/43	20.9%	3/17	17.6%	12/60	20.0%
Acceptability of methods to regulator	Not at all to somewhat concerned	33/43	76.7%	4/17	23.5%	37/60	61.7%
	Moderately to extremely concerned	10/43	23.3%	13/17	76.5%	23/60	38.3%

Acronyms: CTU: Clinical Trial Unit; AE: adverse event

For peer review only

Appendix

Table A11: Solutions to support a change in adverse event (AE) analysis practice

		CTU/Public (N=38)		Industry (N=6)		Overall (N=68)	
**Change**		**n/N**	**%**	**n/N**	**%**	**n/N**	**%**
Software/code development is needed	Strongly disagree/disagree	9/43	20.9%	6/17	35.3%	15/60	25.0%
	Agree/strongly agree	28/43	65.1%	10/17	58.8%	38/60	63.3%
	Don't know	6/43	14.0%	1/17	5.9%	7/60	11.7%
Training specifically for AE analysis is needed	Strongly disagree/disagree	1/43	2.3%	1/17	5.9%	2/60	3.3%
	Agree/strongly agree	42/43	97.7%	16/17	94.1%	58/60	96.7%
Guidance on appropriate AE analysis is needed e.g. case studies, tutorials within open access journals	Agree/strongly agree	43/43	100.0%	17/17	100.0%	60/60	100.0%
Are there any other solutions in addition to those stated above that would support a change in AE analysis practice?	No	34/43	79.1%	7/17	41.2%	41/60	68.3%
	Yes	9/43	20.9%	10/17	58.8%	19/60	31.7%

Acronyms: CTU: Clinical Trial Unit; AE: adverse event

For peer review only

For peer review only - http://bmjopen.bmj.com/site/about/guidelines.xhtml

BMJ Open

Understanding current practice, identifying barriers and exploring priorities for Adverse Event analysis in Randomised Controlled Trials: an online, cross-sectional survey of statisticians from academia and industry

Journal:	*BMJ Open*
Manuscript ID	bmjopen-2020-036875.R1
Article Type:	Original research
Date Submitted by the Author:	29-Apr-2020
Complete List of Authors:	Phillips, Rachel; Imperial College London, Faculty of Medicine, School of Public Health<br>Cornelius, Victoria; Imperial College London, Faculty of Medicine, School of Public Health
**Primary Subject Heading**:	Research methods
Secondary Subject Heading:	Research methods
Keywords:	Clinical trials < THERAPEUTICS, Adverse events < THERAPEUTICS, STATISTICS & RESEARCH METHODS

SCHOLARONE™
Manuscripts

I, the Submitting Author has the right to grant and does grant on behalf of all authors of the Work (as defined in the below author licence), an exclusive licence and/or a non-exclusive licence for contributions from authors who are: i) UK Crown employees; ii) where BMJ has agreed a CC-BY licence shall apply, and/or iii) in accordance with the terms applicable for US Federal Government officers or employees acting as part of their official duties; on a worldwide, perpetual, irrevocable, royalty-free basis to BMJ Publishing Group Ltd ("BMJ") its licensees and where the relevant Journal is co-owned by BMJ to the co-owners of the Journal, to publish the Work in this journal and any other BMJ products and to exploit all rights, as set out in our licence.

The Submitting Author accepts and understands that any supply made under these terms is made by BMJ to the Submitting Author unless you are acting as an employee on behalf of your employer or a postgraduate student of an affiliated institution which is paying any applicable article publishing charge ("APC") for Open Access articles. Where the Submitting Author wishes to make the Work available on an Open Access basis (and intends to pay the relevant APC), the terms of reuse of such Open Access shall be governed by a Creative Commons licence – details of these licences and which Creative Commons licence will apply to this Work are set out in our licence referred to above.

Other than as permitted in any relevant BMJ Author's Self Archiving Policies, I confirm this Work has not been accepted for publication elsewhere, is not being considered for publication elsewhere and does not duplicate material already published. I confirm all authors consent to publication of this Work and authorise the granting of this licence.

Understanding current practice, identifying barriers and exploring priorities for Adverse Event analysis in Randomised Controlled Trials: an online, cross-sectional survey of statisticians from academia and industry

Authors:

Rachel Phillips* - Imperial College London, London, United Kingdom

Victoria Cornelius - Imperial College London, London, United Kingdom

* Corresponding author

Address: Imperial Clinical Trials Unit, Imperial College London, 1st Floor Stadium House, 68 Wood Lane, London, W12 7RH

Email: r.phillips@imperial.ac.uk

Telephone: 020 759 49356

* Funded by a NIHR doctoral research fellowship to undertake this work (Reference: DRF-2017-10-131)

Word Count: 3998

For peer review only - http://bmjopen.bmj.com/site/about/guidelines.xhtml

Abstract

Objectives

To gain a better understanding of current adverse event (AE) analysis practices and the reasons for the lack of use of sophisticated statistical methods for AE data analysis in randomised controlled trials (RCTs), with the aim of identifying priorities and solutions to improve practice.

Design

A cross-sectional, online survey of statisticians working in clinical trials, followed-up with a workshop of senior statisticians working across the United Kingdom.

Participants

We aimed to recruit into the survey a minimum of one statistician from each of the 51 UK Clinical Research Collaboration registered clinical trial units (CTUs) and industry statisticians from both pharmaceuticals and clinical research organisations.

Outcomes

To gain a better understanding of current AE analysis practices, measure awareness of specialist methods for AE analysis and explore priorities, concerns and barriers when analysing AEs.

Results

Thirty-eight (38/51; 75%) CTUs, five (5/7; 71%) industry and twenty-one attendees at the 2019 PSI conference participated in the survey. Of the 64 participants that took part, forty-six participants were classified as public sector participants and eighteen as industry participants. Participants indicated that they predominantly (80%) rely on subjective comparisons when comparing AEs between treatment groups. Forty percent were aware of specialist methods for AE analysis but only 13% had undertaken such analyses. All participants believed guidance on appropriate AE analysis

For peer review only

and 97% thought training specifically for AE analysis is needed. These were both endorsed as solutions by workshop participants.

Conclusions

This research supports our earlier work that identified sub-optimal AE analysis practices in RCTs and confirms the under use of more sophisticated AE analysis approaches. Improvements are needed and further research in this area is required to identify appropriate statistical methods. This research provides a unanimous call for the development of guidance, as well as training on suitable methods for AE analysis to support change.

Keywords

Randomised controlled trials; adverse events; harms; adverse drug reactions; survey; statisticians; clinical trials units; industry; analysis.

For peer review only

For peer review only - http://bmjopen.bmj.com/site/about/guidelines.xhtml

Article summary: Strengths and limitations of this study

- A high response rate was achieved from UKCRC CTU and industry statisticians invited to participate in this survey.
- There was some level of self-selection to participation and as such, there is a possibility that participants had an increased interest in adverse event analysis and are not fully representative of the clinical trial community.
- The survey was followed up with a workshop of senior statisticians from across the United Kingdom, which represents more of a general interest group.
- The survey provides insight and essential starting points to identify areas of focus to help support a change to improve adverse event analysis practices.

For peer review only

INTRODUCTION

Randomised controlled trials (RCTs) are a valuable source of information when establishing the harm profile of medicinal products. They provide a controlled comparison of adverse event rates, thus allowing causality to be evaluated and potential detection of adverse drug reactions. Adverse events are events that may or may not be related to the treatment under investigation, and adverse drug reactions are events classified as related to the treatment under investigation.[†] Reviews of published RCT reports have demonstrated that harms data is not being analysed to its full potential.[1-5] Most notable inadequacies include ignoring information on repeated events and dichotomising continuous clinical and laboratory outcomes; with binary counts often presented using simple tabulations, indicating whether an event did or did not occur. Little formal analysis is performed but a comprehensive methods review undertaken by the authors revealed that there have been many published statistical methods proposed specifically to analyse adverse event data for both the interim and final analysis. These include utilising time-to-event approaches, Bayesian methods that can incorporate prior information and visual analysis.[6, 7] Many of the proposed methods could be adopted into current practice with relative ease. Chuang-Stein and Xia have proposed examples of industry strategies adopting such methods.[8] Previous research has demonstrated that these methods are not used for the analysis presented in the primary results publication. In a recent systematic review of 184 published reports in high impact journals, there are no examples of these proposed methods being used, with authors preferring simple approaches predominantly presenting frequencies and percentages of events.[1, 5] The statistical methods proposed for adverse event

[†] An adverse event is defined as ‘any untoward medical occurrence that may present during treatment with a pharmaceutical product but which does not necessarily have a causal relationship with this treatment’. An adverse drug reaction is defined as ‘a response to a drug which is noxious and unintended …’ where a causal relationship is ‘at least a reasonable possibility’.

For peer review only - http://bmjopen.bmj.com/site/about/guidelines.xhtml

analysis identified in the methodology review also had minimal citations, which further suggests uptake of these methods is low.[1, 6, 7]

In addition, there is a problem with the reporting of adverse events and the selection of events to include in journal articles. Many reviews have established poor quality reporting in journal articles of adverse event data from RCTs.[9-15] Also it is often not possible to include all adverse events in the primary RCT publication and authors need to select events for a pertinent summary. To achieve this there is a prevalent practice of relying on arbitrary rules to select events to report, which can introduce reporting biases leaving out important adverse events. This also creates a barrier to establishing an accurate harm profile.[3, 16]

Understanding the reasons for the low uptake of these statistical methods will help identify solutions to improve the analysis of adverse events in RCTs. We undertook a survey of UK statisticians working in clinical trials to investigate their current practice when analysing adverse events, to measure their awareness of available methods for adverse event analysis, and to explore their priorities, concerns and identify any perceived barriers when analysing adverse events.

METHODS

Study design

A cross-sectional, online survey of UK Clinical Research Collaboration (CRC) clinical trial unit (CTU) and industry statisticians from both pharmaceuticals and clinical research organisations (CROs) was conducted. We aimed to recruit a minimum of one statistician from each of the 51 UKCRC registered

For peer review only - http://bmjopen.bmj.com/site/about/guidelines.xhtml

CTUs and from a sample of pharmaceutical companies and CROs in the UK to gain an industry perspective. The survey was followed-up with a workshop at the UKCRC biannual statisticians' operations group meeting where survey results were presented and areas for improvements and priorities were discussed.

Survey development

The survey was developed using information from current guidance and previous research that examined barriers to the uptake of new methodology.[17-20] Topics covered included questions about current practice and factors influencing adverse event analysis performed; barriers encountered when analysing adverse events; concerns regarding adverse event analysis; awareness and opinions of specialist methods for adverse event analysis; concerns and barriers of implementing specialist methods; and opinions on potential solutions to support a change in adverse event analysis practice.

Questions were predominantly closed form but where appropriate open-ended questions were included to allow for detailed responses and comments. Responses were measured using Likert scales. Survey questions for UKCRC CTU and industry statisticians were identical (appendix item 1). The survey was piloted on clinical trial statisticians (n= 6) at three CTUs prior to launching nationwide to ensure understanding of the questions, whether sufficient response categories had been included, and if certain questions were consistently left unanswered, as well as the usability and functionality of the online platform hosted by SurveyMonkey.[21]

Sampling and Recruitment

For peer review only - http://bmjopen.bmj.com/site/about/guidelines.xhtml

We targeted a population that we knew to be predominantly involved in the analysis of adverse events in clinical trials. Specifically, the UKCRC CTU Statistics Operation network supported the survey and contacted each of the 51 registered CTUs' senior statisticians on behalf of the study team. Email invitations were also sent directly to a convenience sample of seven senior statistical contacts working in UK based pharmaceuticals (Astra-Zeneca, Boehringe-Ingelheim, Glaxo-Smith-Kline (GSK), Novartis and Roche) and CROs (Cytel and IQVIA). The invitations requested that one statistician within the unit or organisation complete the survey. Reminder emails were sent to non-responders. The survey opened in April 2019 and remained open for 8 weeks. We also created an open platform for participants that was promoted at the June 2019 Promoting Statistical Insights (PSI) conference, the Effective Statistician podcast broadcast in July 2019, and Twitter and LinkedIn platforms. This platform remained open for 10 weeks. Participants that successfully completed the survey were automatically entered into a prize draw to win £50 worth of gift vouchers.

The invitation to participate in the study included the participant information sheet (appendix item 2), which was also included at the beginning of the survey before participants formally entered. Participants were encouraged to read the information sheet and discuss the study with others or contact the research team if they wished. If invitees were happy to enter into the trial at that point their consent was taken as implied upon submission of the completed survey.

For peer review only

Participants

Statisticians with experience of planning and preparing the final analysis reports for pharmacological RCTs were invited to participate.

For peer review only - http://bmjopen.bmj.com/site/about/guidelines.xhtml

Analysis

Descriptive analysis was undertaken, primarily including frequencies and proportions for each questionnaire item and where appropriate was accompanied with visual summaries.[22] The frequency and proportion of participants that showed support for an item was calculated by combining the 'always' and 'often' or 'strongly agree' and 'agree' categories. Participants were classified according to affiliation into either CTU/public sector or industry sector and analysis was stratified by sector. Response rates were calculated for groups of participants where known.

Patient and public involvement

This survey forms part of a wider research project that was developed with input from a range of patient representatives. There were no patients directly involved in this survey but the original proposal and patient and public involvement (PPI) strategy were reviewed by service user representatives (with experience as clinical trial participants and PPI advisors) who provided advice specifically with regard to communication and dissemination to patient and public groups.

RESULTS

Participant flow

Invitations were sent to fifty-one CTU/public sector and seven industry contacts. Thirty-eight (75%) units and five (71%) industry contacts participated in the survey giving an overall response rate of 74%. Twenty-four people consented to participate via the open platform, of which 21 participated in

For peer review only - http://bmjopen.bmj.com/site/about/guidelines.xhtml

the survey. Eight of which were included in the CTU/public sector group and thirteen in the industry sector. In total 64 participants took part in the survey with n=46 from the CTU/public sector and n=18 from industry (appendix figure A1).

Participant characteristics

Overall, more than 80% of responders worked on studies of more than 100 participants, and 80% worked on phase II/III trials. A greater proportion of industry participants were working on phase I/dose finding trials compared to CTU/public sector participants (22% vs 2%) (figure 1). The mean number of years of experience was 12.8 (SD 8.3) (median 11.5 years, range (1-35 years)) (table 1).

Table 1: Participant characteristics by sector and overall

		CTU/Public (N=46)		Industry (N=18)		Overall (N=64)	
**Characteristics**		**n/N**	**%**	**n/N**	**%**	**n/N**	**%**
**Typical trial size**	1-10	0/46	0.0%	1/18	5.6%	1/64	1.6%
	11-50	0/46	0.0%	1/18	5.6%	1/64	1.6%
	51-100	6/46	13.0%	4/18	22.2%	10/64	15.6%
	101-500	28/46	60.9%	9/18	50.0%	37/64	57.8%
	>500	12/46	26.1%	3/18	16.7%	15/64	23.4%
**Work setting**	Academic institution	38/46	82.6%	0/18	0.0%	38/64	59.4%
	CRO	1/46	2.2%	7/18	38.9%	8/64	12.5%
	NHS trust	5/46	10.9%	0/18	0.0%	5/64	7.8%
	Pharmaceutical	0/46	0.0%	9/18	50.0%	9/64	14.1%
	Other	2/46	4.3%	2/18	11.1%	4/64	6.3%
**Speciality[1]**	No	23/46	50.0%	7/18	38.9%	30/64	46.9%
	Yes	23/46	50.0%	11/18	61.1%	34/64	53.1%
**Typical trial phase**	Phase I/Dose-finding	1/46	2.2%	4/18	22.2%	5/64	7.8%
	Phase II/III	38/46	82.6%	12/18	66.7%	50/64	78.1%
	Phase IV	7/46	15.2%	2/18	11.1%	9/64	14.1%
**Years of experience**	Mean (SD)	12.0	(7.2)	14.7	(10.7)	12.8	(8.3)
	Median (min, max)	12.0	(1, 30)	15.5	(1, 35)	11.5	(1, 35)

Acronyms: CTU: Clinical Trial Unit; CRO: Clinical Research Organisation; SD: standard deviation; min: minimum; max: maximum

[1]Participants were asked if there was a clinical area they predominantly worked on.

For peer review only - http://bmjopen.bmj.com/site/about/guidelines.xhtml

Current analysis practice

Seventy-five percent of participants reported that they present both '*the number of participants with at least one event'* and '*the number of events'*, 13% reported only presenting '*the number with at least one event'*, 2% stated that they only present '*the number of events'* and 11% reported not presenting either of these (table 2 and appendix table A1 for free text comments).

Ninety percent of participants reported that they use frequencies and percentages to summarise adverse event data, less than 20% reported use of risk differences (16%), odds ratios (16%) or risk ratios (17%), just under a quarter reported use of incidence rate ratios (23%) (table 2). Several participants included comments (n=5) that the summary statistic used for analysis depended on the specific study being analysed.

When comparing adverse event rates between treatment arms 80% of participants reported typically relying on subjective comparisons, 33% compare rates using hypothesis tests, and 22% use 95% confidence intervals (CIs) as a means to examine the null hypothesis of no difference. CTU/public sector participants reported wider use of both hypothesis tests (39% CTUs/public sector versus 17% industry) and 95% CIs (26% CTUs/public sector versus 11% industry) (table 2). Four free text comments cautioned against the use of testing.

For peer review only - http://bmjopen.bmj.com/site/about/guidelines.xhtml

Table 2: Adverse event (AE) information typically presented by sector and overall

	CTU/Public (N=46)		Industry (N=18)		Overall (N=64)	
**Information presented**	**n/N**	**%**	**n/N**	**%**	**n/N**	**%**
Number of participants with at least one event	4/46	8.7%	4/18	22.2%	8/64	12.5%
Number of events	1/46	2.1%	0/18	0.0%	1/64	1.6%
Both of the above	36/46	78.3%	12/18	66.7%	48/64	75.0%
None of the above	5/46	10.9%	2/18	11.1%	7/64	10.9%
Other[1]	16/46	34.8%	6/18	33.3%	22/64	34.4%
**Descriptive and summary statistics†**						
Frequencies	42/46	91.3%	16/18	88.9%	58/64	90.6%
Percentages	43/46	93.5%	14/18	77.8%	57/64	89.1%
Risk difference	5/46	10.9%	5/18	27.8%	10/64	15.6%
Odds ratio	7/46	15.2%	3/18	16.7%	10/64	15.6%
Risk ratio	6/46	13.0%	5/18	27.8%	11/64	17.2%
Incidence rate ratio[2]	8/46	17.4%	7/18	38.9%	15/64	23.4%
Other[3]	6/46	13.0%	4/18	22.2%	10/64	15.6%
**AE comparison†**						
Subjective comparison	36/46	78.3%	15/18	83.3%	51/64	79.7%
Exclusion of null through 95% confidence interval	12/46	26.1%	2/18	11.1%	14/64	21.9%
Hypothesis test/p-value	18/46	39.1%	3/18	16.7%	21/64	32.8%
Other[4]	4/46	8.7%	5/18	27.8%	9/64	14.1%
**Awareness of any published methods specifically to analyse AEs**						
No	25/44	56.8%	4/17	23.5%	29/61	47.5%
Yes	11/44	25.0%	12/17	70.6%	23/61	37.7%
Don't know	8/44	18.2%	1/17	5.9%	9/61	14.8%
	25/44	56.8%	4/17	23.5%	29/61	47.5%
**Undertaken any specialist AE analysis not mentioned in your previous response**						
No	38/43	88.4%	14/17	82.4%	52/60	86.7%
Yes	5/43	11.6%	3/17	17.6%	8/60	13.3%

Acronyms: CTU: Clinical Trial Unit; AE: adverse event

† Participants were able to provide multiple responses to this question.

[1] Other ways of presenting AE information included presenting information on: overall number of events (n=2); number of patients experiencing 0, 1, 2 etc. events and number of AEs per patient (n=2); duration (n=1); relatedness (n=1) and severity (n=7) (full free text comments in appendix table A1).

[2] Incorporates free text comments that described summaries synonymous with incidence rate ratios.

[3] Included a comment that a participant presents the "median number (IQR)" of events.

[4] Other comments related to the calculation of confidence intervals for precision (n=2), one indicated use of a graphical summary (n=1) and four cautioned against the use of testing.

Just under 40% stated that they were <u>aware</u> of appropriate methods published specifically for adverse event analysis in RCTs (table 2). There were five broad groups of methods mentioned, including Bayesian methods to analyse low frequencies (n=1); standard regression modelling

For peer review only - http://bmjopen.bmj.com/site/about/guidelines.xhtml

approaches such as Poisson, negative binomial and survival approaches (n=6); methods to analyse incidence rates (n=5); meta-analysis approaches for rare events (n=2); and graphical approaches (n=2) (full text comments in appendix table A2). Participants also directed us to theoretical and applied examples in the literature (n=6) (full free text comments in appendix table A2).[18, 23-27]

Only thirteen percent reported undertaking specialist adverse event analysis (table 2), of which five participants provided details. Two reported use of time-to-event approaches, one used data visualisations, one use Bayesian methods and one incorporated repeated events (full free text comments are reported in appendix table A3).

Of the participants who reported that they were aware of specialist adverse event analysis methods, we asked opinions on why such methods were not more widely used. Just over a quarter thought limited use was due to technical complexity (27%); over a third thought it could be due to trial characteristics such as unsuitability of sample sizes (36%) and the number of different adverse events experienced in trials (36%); and 46% thought methods were too resource intensive and methods were not suitable for typical adverse event rates observed (appendix table A4).

Over three-quarters (77%) of participants provided further reasons for lack of use of specialist methods. Reasons were characterised into comments relating to: concerns with the suitability of methods in relation to trial characteristics and nature of adverse event data (n=7); opposition and a lack of understanding from clinicians (n=5); a lack of need for such methods (n=3); a desire to keep analysis consistent with historical analysis (n=3); and training and resources (n=1) (appendix table A5).

For peer review only - http://bmjopen.bmj.com/site/about/guidelines.xhtml

For peer review only

Influences, barriers and concerns

The most common influences for the adverse event analysis performed were cited as the chief investigator's preference for simple approaches (78%), the observed adverse event rates (76%) and the size of the trial (73%). Over 60% of participants felt that the statistician preferred simple approaches for adverse event analysis (68%), and the number of different adverse events experienced in a trial were influential (65%). Less than 50% of participants thought that journals (48%) or regulators (48%) preferred simple approaches but there was a notable difference by sector. A greater proportion of industry participants thought regulators preferred simple approaches (67% versus 40%); and a greater proportion of CTU/public sector participants thought journals preferred simple approaches (56% versus 28%) (figure 2 and appendix table A6).

Seventy-nine percent of participants indicated that there are a lack of training opportunities to learn what methods are appropriate for adverse event analysis, two-thirds (66%) believed that there is a lack of awareness of appropriate methods and 58% believed there is a lack of knowledge to implement appropriate methods. Approximately 60% of participants thought that trial characteristics including trial sample size (61%), number of different adverse events experienced (61%) and adverse event rates (65%) were barriers when analysing such data. Only a third (34%) of participants agreed that a lack of statistical software/code to implement appropriate methods was a barrier (figure 2 and appendix table A7).

The majority of participants (84%) held the opinion that there are a lack of examples for appropriate analysis methods in the applied literature and 44% of participants thought that there are a lack of appropriate analysis methods. Over half of participants indicated that statisticians (69%), journals

For peer review only - http://bmjopen.bmj.com/site/about/guidelines.xhtml

(60%) and chief-investigators (52%) do not give adverse event data the same priority as the primary efficacy outcome. Only 13% of participants believe that regulators do not prioritise adverse event data but nearly a quarter (24%) felt unable to comment on regulators priorities (figure 2 and appendix table A8).

Concerns and solutions

When participants were asked to think about available methods for adverse event analysis the most common concern, which was held by 38% of participants was acceptability of methods to regulators. This differed substantially by sector with only 23% of CTU/public sector participants holding this belief compared to 77% of industry participants. Twenty percent of participants were concerned about the acceptability of methods to the chief investigator and journals and 32% were concerned about the robustness of methods (figure 2 and appendix table A9).

All participants believed that guidance on appropriate adverse event analysis is needed, 97% thought training specifically for adverse event analysis is needed, and 63% thought new software or code is needed (figure 2 and appendix table A10). Just under a third (32%) of participants offered solutions to support change in adverse event analysis practices. These included suggestions regarding improved standards or calls for changes from journals, registries and regulators (n=8); development of guidance, education and engaging with the medical community (n=9); and analysis (n=3) (appendix table A11).

Thirty percent of participants raised other items not listed in the survey regarding current adverse event analysis practices, these covered the following themes: minimum summary information that

For peer review only - http://bmjopen.bmj.com/site/about/guidelines.xhtml

participants would expect to be reported for adverse event data such as “numbers and percentages” (n=2); changes to analysis practice that could or have been made such as “use of graphical methods” (n=8); concerns about the quality and collection of adverse event data (n=3); and general comments and criticisms about current adverse event analysis and reporting practices (n=4) (appendix table A12).

In the follow-up workshop of senior statisticians (n=52 from 43 UKCRC registered CTUs) attending the UKCRC biannual statisticians’ operations meeting in November 2019, participants were asked to rate the need to improve analysis practices for adverse event data on a scale of 0-100 (indicating low to high priority). The mean score was 66 (SD 16.2) (median 71 (range 9, 88)) (n=44). In discussions, the following themes were highlighted as priorities to take forward: development of guidelines; identification of appropriate analysis methods; exploring integration of qualitative information; and ensuring consistency of information reported including development of core harm outcomes by drug class.

DISCUSSION

Despite RCTs being a valuable source of data to compare rates of adverse events between treatment groups and provide an opportunity to assess causality, analysis and reporting practices are often inadequate.[1-4, 9-15] This survey of statisticians from the UK public and private sectors has established a more detailed picture of clinical trial statisticians’ adverse event analysis practices and builds on our previous research which evaluated adverse event analysis practices reported in journal articles.[1] It has identified priorities and concerns including influences, barriers and opinions to be addressed in future work to improve adverse event analysis.

For peer review only - http://bmjopen.bmj.com/site/about/guidelines.xhtml

Results were broadly similar across public and industry sectors with the only notable differences being the greater use of hypothesis testing and 95% confidence intervals as a means to compare adverse events rates between treatment groups by CTU participants, a more predominant belief by industry participants that regulators preferred simple approaches to adverse event analysis, and a greater concern about acceptability of methods to regulators by industry participants. Across sectors, there was unanimous support that guidance and training on appropriate adverse event analysis is needed.

Survey responses indicated that 75% of statisticians produce tables with both the number of participants with at least one event and the total number of events. This is substantially higher than reported in reviews of published articles, which found between 1% and 9% reported both.[1-3] The number of total events experienced can give a better summary of impact to patients' quality-of-life but it seems this is often omitted from journal articles with reviews identifying only 6% to 7% of published articles reporting this information.[1, 4] Reported use of 95% confidence intervals were similar to that reported in journal articles (22% compared to 20%) but reported use of hypothesis testing was lower than what was found in journal articles (32% compared to a range of 38% to 47%).[1-3] Reasons for these disparities are not known but could include journals editors requesting such analyses is undertaken to compare groups, or at the request of the chief investigator, which is supported by survey responses indicating a preference for simple approaches from both groups. It could also be that the survey participants were restricted to those working in CTUs and industry, and are perhaps not fully representative of those undertaking and reporting clinical trial results.

Many methods have been specifically proposed for adverse event analysis in RCTs and there was a moderate level of awareness of these methods (40%) but in line with our review of journal articles we found uptake to be minimal (13%).[6, 7] Whilst not directly comparable, our results are also closely

For peer review only - http://bmjopen.bmj.com/site/about/guidelines.xhtml

aligned with the results of a survey of industry statisticians and clinical safety scientists, undertaken by Colopy and colleagues that indicated a reliance on traditional methods for descriptive statistics and frequentist approaches when analysing harm outcomes.[28]

This survey did not specifically ask participants about their use of graphics to display adverse event data but a similar proportion of participants indicated use of such summaries in free text comments as identified in our review of journal articles (9% vs 12%).[1] However, these figures were both substantially lower than the 37% that indicated use of static visual displays for study level adverse event analysis in the survey of industry statisticians.[28] This could reflect the use of more advanced graphical approaches for internal reports.

Education via training and guidance for statisticians and trialists about appropriate adverse event analysis could lead to improved practice and were both strongly endorsed as solutions by participants of both the survey and workshop. Guidelines such as the harms extension to CONSORT; the pharmaceutical industry standard from the Safety Planning, Evaluation and Reporting Team (SPERT); and the joint pharmaceutical/journal editor collaboration guidance on reporting of harm data in journal articles already exist and make several recommendations for analysing adverse events.[17, 18, 29] However, adherence to the CONSORT Harms checklist has been shown to be poor; and whilst the impact of the Lineberry et al. guidance and the Crowe et al. guidance has not been formally evaluated, our review of adverse event analysis practices indicate uptake of suggestions within these guidelines such as “reporting CIs around absolute risk differences” and to “include both the number of events (per person time) and the number of patients experiencing the event” to be minimal.[1, 2, 4, 14, 15] It has also been argued that such guidelines do not go far enough and fail to account for the complex nature of harm outcomes data.[5] Tutorial papers or case studies detailing examples of appropriate analysis could lead to wider adoption of such recommendations and to

For peer review only - http://bmjopen.bmj.com/site/about/guidelines.xhtml

For peer review only

improvements in analysis practices, and development of such resources was highlighted as a priority by workshop participants. Whilst the acquirement of the necessary knowledge and skills to implement new methods is essential, so too is increasing awareness of good practices and alternative methods. Guidance or tutorial papers can be useful to increase knowledge, but wide dissemination and promotion to encourage use is vital if we are to improve practice.

A change in attitude from both statisticians and the wider research community away from doing what they have always done is also needed. Journals and regulators play a leading role in influencing good practice and could influence statisticians and trialists practice through policy change. The New England Journal of Medicine has already updated their policy to demand that evidence about both benefits and harms of treatments include point estimates and margins of error; and require no adjustment for multiplicity where significance tests are performed for harm outcomes "*Because information contained in the safety endpoints may signal problems within specific organ classes, the editors believe that the type I error rates larger than 0.05 are acceptable*."[30] A journal wide initiative to adopt existing guidelines, for example, through the mandatory submission of the CONSORT harms checklist would be one simple, initial step towards change.

Trial design and the nature of adverse event outcomes can also hinder the analyses performed. Unlike efficacy outcomes, which are well defined and limited in number from the outset, harm outcomes are numerous, undefined and contain additional information on severity, timing and duration, and number of occurrences, which all need to be considered. More careful consideration of harm outcomes when designing, analysing and reporting trials will help produce a more balanced view of benefits and risks.

For peer review only - http://bmjopen.bmj.com/site/about/guidelines.xhtml

Improved analysis could be achieved through adoption of existing or development of more appropriate methods for adverse event data. Several participants mentioned adverse event analysis approaches we believe warrant exploring, including time-to-event analyses, data-visualisations and Bayesian methods. Ultimately, with the aim of helping to identify signals for adverse drug reactions enabling a clearer harm profile to be presented. This is supported by feedback obtained at the workshop and the earlier findings of Colopy et al. who concluded that statisticians should help "minimize the submission of uninformative and uninterpretable reports" and thus present more informative information regarding likely drug-event relationships.[28]

Participants of both the survey and workshop raised concerns about the quality and reporting of adverse event data from RCTs. We agree that if adverse event data is not robust the analysis approach is redundant as the results will not be accurate. Therefore, procedures should be put in place at the trial design stage to mitigate problems with adverse event data collection, including for example, development of validated methods for data collection and clear, standardised instructions for those involved in the detection and collection.[3, 31]

Strengths and limitations

Through support of the UKCRC CTU network and utilisation of personal contacts, we were able to achieve a high response rate for the survey. After invitations were sent there was no way to ensure that responses were restricted to one per unit or organisation. However, dissemination via the UKCRC to senior statisticians within units and personal, senior contacts within industry would have ensured some quality control. There was some level of self-selection for those recruited via the open platform and as such, there is a possibility that these participants had an increased interest in

For peer review only - http://bmjopen.bmj.com/site/about/guidelines.xhtml

adverse event analysis and are not fully representative of the clinical trial community. We also did not have any information on non-responders and as such cannot characterise any potentially relevant differences that could affect the generalisability of our results. This survey provides insight and essential starting points to identify areas of focus to help support a change to improve adverse event analysis practice. Many of the opinions raised in the survey were echoed by the workshop attendees who represented more of a general interest group.

Conclusions

This research demonstrates that there is a moderate level of awareness of appropriate statistical methods for adverse event analysis but that these methods are not being used by statisticians and supports our earlier work identifying adverse event analysis practices in RCTs as sub-optimal. Participants made a unanimous call for guidance on appropriate methods for adverse event analysis and training to support change. Feedback from both survey and workshop participants is that further research is needed to identify the most appropriate statistical methods for adverse event data analysis from all those available.

For peer review only

Acknowledgments

The authors would like to thank:

Dr Odile Sauzet for discussing the survey design in the initial stages of development.

Louise Williams (UKCRC) for her support, in particular circulating the survey to UKCRC registered CTUs and helping us achieve such a high response rate, and Professor Carrol Gamble and Professor Catherine Hewitt (UKCRC statistical operational group chairs) for supporting this project.

Alexander Schacht for inviting us on to his podcast to promote the survey and circulating to his contacts through LinkedIn.

Dr Suzie Cro, Nicholas Johnson, Anca Chis Ster, Emily Day, Fiona Reid and Professor Carrol Gamble for providing feedback on survey content and platform. Also to Dr Suzie Cro for her help in facilitating the workshop at the UKCRC biannual statisticians' operations group meeting.

COMPETING INTERESTS

None declared.

ETHICS

This study was granted ethical approval by the Imperial College Joint Research Compliance Office (ICREC reference: 19IC5067).

FUNDING

This research was supported by the NIHR grant number DRF-2017-10-131.

For peer review only - http://bmjopen.bmj.com/site/about/guidelines.xhtml

DATA SHARING STATEMENT

Survey data are available from the Zenodo data repository.

AUTHOR CONTRIBUTIONS

RP and VC conceived the idea, designed and ran the survey. RP performed the data analysis, interpreted the results and wrote the manuscript. VC interpreted the results, provided critical revision of the manuscript and supervised the project.

Figure legends

Figure 1: Participant characteristics by sector and overall

(Acronyms: CRO: Clinical Research Organisation; Pharma: Pharmaceuticals; CTUs: Clinical Trials Units)

Figure 2: Survey results by sector (a) Influences on the analysis of AEs (b) Barriers to improve AE analysis (c) Opinions on current AE analysis (d) Reasons for concern with existing methods for AE analysis (e) Potential solutions for change (improving AE analysis)

(Acronyms: CTU: Clinical Trials Unit; CI: Chief Investigator; AE: adverse event)

For peer review only - http://bmjopen.bmj.com/site/about/guidelines.xhtml

REFERENCES

1. Phillips R, Hazell L, Sauzet O, et al. Analysis and reporting of adverse events in randomised controlled trials: a review. *BMJ Open* 2019; 9: e024537. 2019/03/04. DOI: 10.1136/bmjopen-2018-024537.
2. Pitrou I, Boutron I, Ahmad N, et al. Reporting of safety results in published reports of randomized controlled trials. *Archives of Internal Medicine* 2009; 169: 1756-1761. DOI: 10.1001/archinternmed.2009.306.
3. Cornelius VR, Sauzet O, Williams JE, et al. Adverse event reporting in randomised controlled trials of neuropathic pain: Considerations for future practice. *PAIN* 2013; 154: 213-220. DOI: 10.1016/j.pain.2012.08.012.
4. Hum SW, Golder S and Shaikh N. Inadequate harms reporting in randomized control trials of antibiotics for pediatric acute otitis media: a systematic review. *Drug Safety* 2018 May 08. DOI: 10.1007/s40264-018-0680-0.
5. Patson N, Mukaka M, Otwombe KN, et al. Systematic review of statistical methods for safety data in malaria chemoprevention in pregnancy trials. *Malaria Journal* 2020; 19: 119. DOI: 10.1186/s12936-020-03190-z.
6. Phillips R, Sauzet O and Cornelius V. Statistical methods for the analysis of adverse event data in randomised controlled trials: a review of available methods. *(Unpublished)*.
7. Phillips R, Cornelius V and Sauzet O. An evaluation and application of statistical methods designed to analyse adverse event data in RCTs. *Trials Conference: 5th International Clinical Trials Methodology Conference, ICTMC 2019 United Kingdom* 2019; 20.
8. Chuang-Stein C and Xia HA. The practice of pre-marketing safety assessment in drug development. *Journal of Biopharmaceutical Statistics* 2013; 23: 3-25. Review. DOI: 10.1080/10543406.2013.736805.
9. Ioannidis JPA and Contopoulos-Ioannidis DG. Reporting of safety data from randomised trials. *The Lancet* 1998; 352: 1752-1753. DOI: 10.1016/S0140-6736(05)79825-1.
10. Edwards JE, McQuay HJ, Moore RA, et al. Reporting of Adverse Effects in Clinical Trials Should Be Improved. *Journal of Pain and Symptom Management* 1999; 18: 427-437. DOI: 10.1016/S0885-3924(99)00093-7.
11. Ioannidis JA and Lau J. Completeness of safety reporting in randomized trials: An evaluation of 7 medical areas. *JAMA* 2001; 285: 437-443. DOI: 10.1001/jama.285.4.437.
12. Maggi CB, Griebeler IH and Dal Pizzol Tda S. Information on adverse events in randomised clinical trials assessing drug interventions published in four medical journals with high impact factors. *Int J Risk Saf Med* 2014; 26: 9-22. 2014/05/07. DOI: 10.3233/JRS-140609.
13. Smith SM, Wang AT, Katz NP, et al. Adverse event assessment, analysis, and reporting in recent published analgesic clinical trials: ACTTION systematic review and recommendations. *PAIN* 2013; 154: 997-1008. DOI: 10.1016/j.pain.2013.03.003.
14. Peron J, Maillet D, Gan HK, et al. Adherence to CONSORT adverse event reporting guidelines in randomized clinical trials evaluating systemic cancer therapy: a systematic review. *J Clin Oncol* 2013; 31: 3957-3963. 2013/09/26. DOI: 10.1200/JCO.2013.49.3981.
15. Favier R and Crépin S. The reporting of harms in publications on randomized controlled trials funded by the "Programme Hospitalier de Recherche Clinique," a French academic funding scheme. *Clinical Trials* 2018; 0: 1740774518760565. DOI: 10.1177/1740774518760565.
16. Mayo-Wilson E, Fusco N, Hong H, et al. Opportunities for selective reporting of harms in randomized clinical trials: Selection criteria for non-systematic adverse events. *Trials* 2019; 20: 553. DOI: 10.1186/s13063-019-3581-3.
17. Ioannidis JA, Evans SW, Gøtzsche PC, et al. Better reporting of harms in randomized trials: An extension of the consort statement. *Annals of Internal Medicine* 2004; 141: 781-788. DOI: 10.7326/0003-4819-141-10-200411160-00009.

For peer review only - http://bmjopen.bmj.com/site/about/guidelines.xhtml

18. Lineberry N, Berlin JA, Mansi B, et al. Recommendations to improve adverse event reporting in clinical trial publications: A joint pharmaceutical industry/journal editor perspective. *BMJ (Online)* 2016; 355: i5078.
19. Love SB, Brown S, Weir CJ, et al. Embracing model-based designs for dose-finding trials. *British journal of cancer* 2017; 117: 332-339. 06/29. DOI: 10.1038/bjc.2017.186.
20. Dimairo M, Julious SA, Todd S, et al. Cross-sector surveys assessing perceptions of key stakeholders towards barriers, concerns and facilitators to the appropriate use of adaptive designs in confirmatory trials. *Trials* 2015; 16: 585-585. DOI: 10.1186/s13063-015-1119-x.
21. Kelley K, Clark B, Brown V, et al. Good practice in the conduct and reporting of survey research. *International Journal for Quality in Health Care* 2003; 15: 261-266. DOI: 10.1093/intqhc/mzg031.
22. Morris TP, Jarvis CI, Cragg W, et al. Proposals on Kaplan–Meier plots in medical research and a survey of stakeholder views: KMunicate. *BMJ Open* 2019; 9: e030215. DOI: 10.1136/bmjopen-2019-030215.
23. Proctor T and Schumacher M. Analysing adverse events by time-to-event models: the CLEOPATRA study. *Pharmaceutical Statistics* 2016; 15: 306-314. DOI: 10.1002/pst.1758.
24. Southworth H. Detecting outliers in multivariate laboratory data. *Journal of Biopharmaceutical Statistics* 2008; 18: 1178-1183.
25. Allignol A, Beyersmann J and Schmoor C. Statistical issues in the analysis of adverse events in time-to-event data. *Pharmaceutical Statistics* 2016; 15: 297-305.
26. Special Issue:Analysis of Adverse Event Data. *Pharmaceutical Statistics* 2016; 15: 287-379.
27. Unkel S, Amiri M, Benda N, et al. On estimands and the analysis of adverse events in the presence of varying follow-up times within the benefit assessment of therapies. *Pharmaceutical Statistics* 2019; 18: 166-183. DOI: 10.1002/pst.1915.
28. Colopy MW, Gordon R, Ahmad F, et al. Statistical Practices of Safety Monitoring: An Industry Survey. *Therapeutic Innovation & Regulatory Science* 2019; 53: 293-300. DOI: 10.1177/2168479018779973.
29. Crowe BJ, Xia HA, Berlin JA, et al. Recommendations for safety planning, data collection, evaluation and reporting during drug, biologic and vaccine development: a report of the safety planning, evaluation, and reporting team. *Clinical Trials* 2009; 6: 430-440. DOI: 10.1177/1740774509344101.
30. Harrington D, D'Agostino RB, Gatsonis C, et al. New Guidelines for Statistical Reporting in the Journal. *New England Journal of Medicine* 2019; 381: 285-286. DOI: 10.1056/NEJMe1906559.
31. Stephens MD, Talbot JC and Routledge PA. *The Detection of New Adverse Reactions*. 4 ed. London: Macmillan Reference 1998.

For peer review only

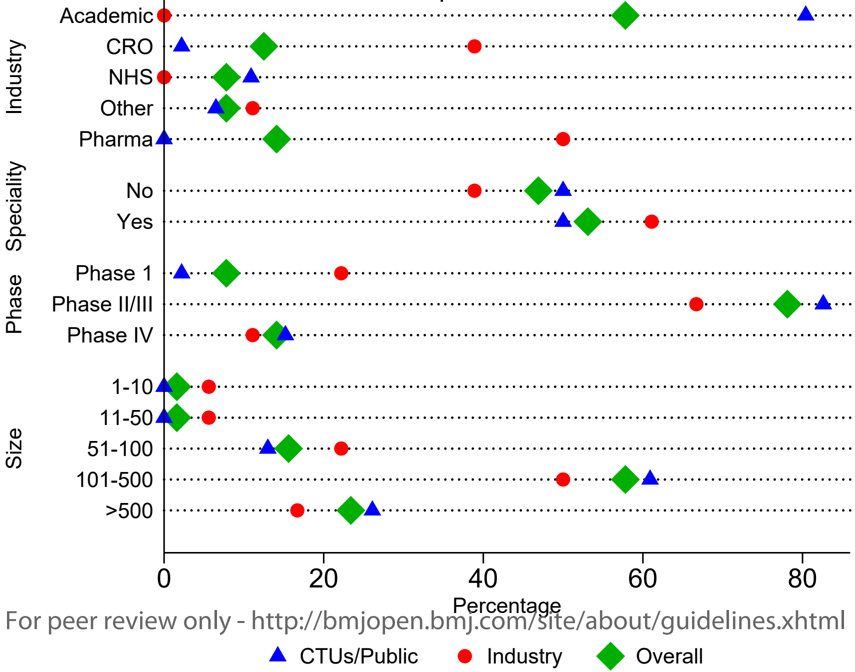

For peer review only - http://bmjopen.bmj.com/site/about/guidelines.xhtml

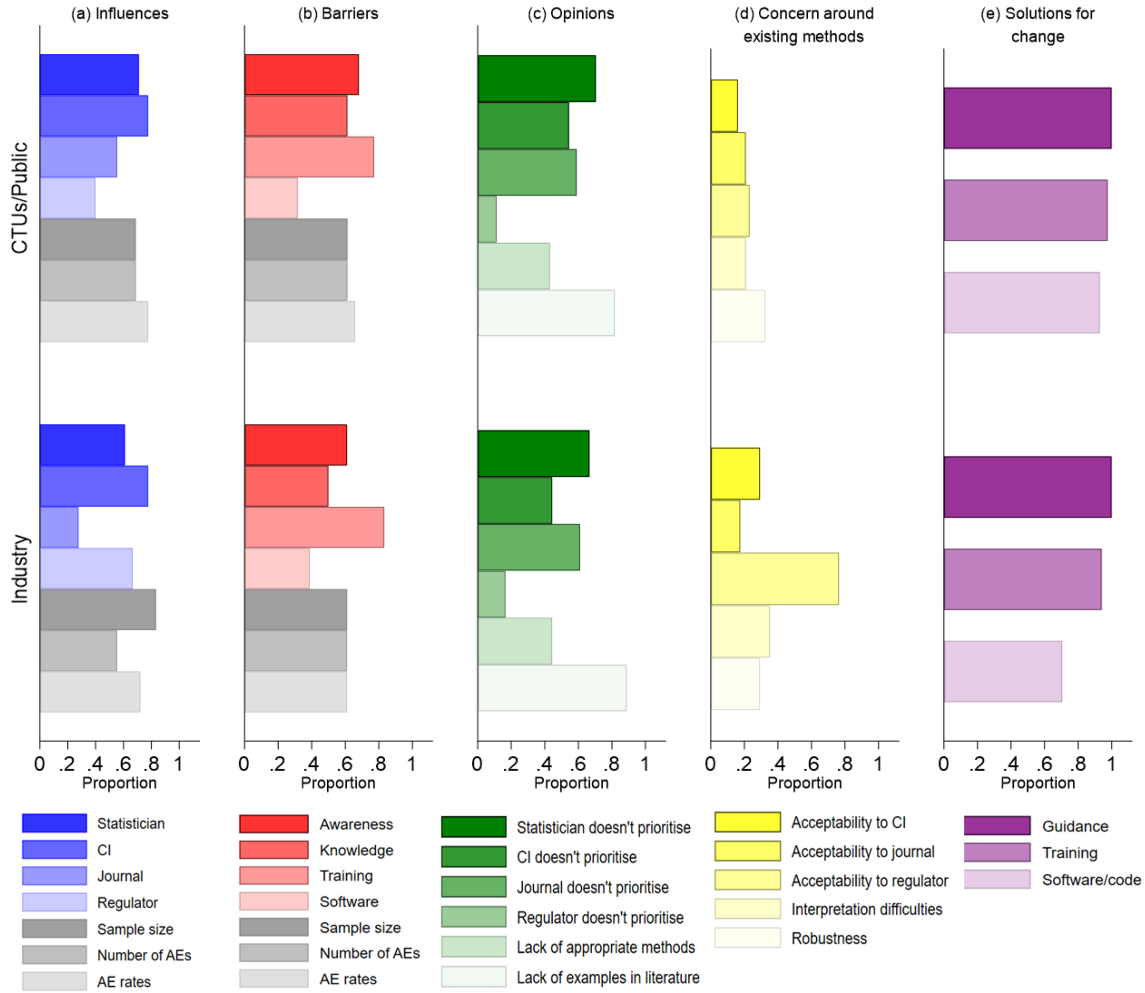
(a) Influences
(b) Barriers
(c) Opinions
(d) Concern around existing methods
(e) Solutions for change
CTUs/Public
Industry
0 .2 .4 .6 .8 1
Proportion
Statistician
CI
Journal
Regulator
Sample size
Number of AEs
AE rates
Awareness
Knowledge
Training
Software
Sample size
Number of AEs
AE rates
Statistician doesn't prioritise
CI doesn't prioritise
Journal doesn't prioritise
Regulator doesn't prioritise
Lack of appropriate methods
Lack of examples in literature
Acceptability to CI
Acceptability to journal
Acceptability to regulator
Interpretation difficulties
Robustness
Guidance
Training
Software/code

Appendix

Item 1: Survey questions

Study Title: Statisticians survey on statistical methods for adverse event data analysis in randomised controlled trials

This survey pertains to the final analysis of AEs reported or screened for in clinical trials. Not predefined specific single safety outcomes of interest or interim analyses.

Number	Question	Response options				
1	How long have you worked as a clinical trial statistician? (Please specify the number of years)					
2	Do you work for:	Academic institution	NHS trust	Pharmaceutical company	Clinical Research Organisation	Other (please specify)
3	Is there a clinical area you predominantly work on? If yes, please specify	No	Yes			
4	What is the typical size of the trials you work on?	1-10	11-50	51-100	101-500	>500
5	What is the typical phase of the trials you work on?	Phase I/Dose-finding	Phase II/III	Phase IV		

Before you proceed we thought it would be helpful for you to know about our recent findings.

We undertook a systematic review of RCT journal reports and found that trials typically report AE data using frequencies (94%) and percentages (87%). They often ignore repeated events (84%) and 47% undertake hypothesis tests despite a lack of power. There is also a common practice to categorise continuous clinical and laboratory outcomes and present as frequencies and percentages (59%). A small proportion (12%) incorporated graphics into the AE analysis.

Number	Question					
	Thinking about analysis methods for AEs:					
6	How often would you say the following influences the analysis performed?					
i	Statistician prefers simple approaches e.g. tables of frequencies and percentages	Always	Often	Not very often	Never	Don't know

For peer review only - http://bmjopen.bmj.com/site/about/guidelines.xhtml

Appendix

	ii	Chief investigator prefers simple approaches e.g. tables of frequencies and percentages	Always	Often	Not very often	Never	Don't know
	iii	Journal prefers simple approaches e.g. tables of frequencies and percentages	Always	Often	Not very often	Never	Don't know
	iv	Regulator prefers simple approaches e.g. tables of frequencies and percentages	Always	Often	Not very often	Never	Don't know
	v	Trial sample size	Always	Often	Not very often	Never	Don't know
	vi	The number of different AEs experienced across the trial	Always	Often	Not very often	Never	Don't know
	vii	AE rates	Always	Often	Not very often	Never	Don't know
7		Thinking about AE analysis you typically perform. In your experience the following is a barrier when analysing AEs:					
	i	Lack of awareness of appropriate methods	Strongly agree	Agree	Disagree	Strongly disagree	Don't know
	ii	Lack of knowledge to implement appropriate methods	Strongly agree	Agree	Disagree	Strongly disagree	Don't know
	iii	Lack of training opportunities to learn what methods are appropriate	Strongly agree	Agree	Disagree	Strongly disagree	Don't know
	iv	Lack of statistical software/code to implement appropriate methods	Strongly agree	Agree	Disagree	Strongly disagree	Don't know
	iv	Trial sample size	Strongly agree	Agree	Disagree	Strongly disagree	Don't know
	v	The number of different AEs experienced across the trial	Strongly agree	Agree	Disagree	Strongly disagree	Don't know
	vi	AE rates	Strongly agree	Agree	Disagree	Strongly disagree	Don't know
8		Thinking about AE analysis. In your opinion:					
	i	Statisticians don't give AE data the same priority as the primary efficacy outcome	Strongly agree	Agree	Disagree	Strongly disagree	Don't know

For peer review only - http://bmjopen.bmj.com/site/about/guidelines.xhtml

Appendix

	ii	Chief investigators don't give AE data the same priority as the primary efficacy outcome	Strongly agree	Agree	Disagree	Strongly disagree	Don't know
	iii	Journals don't give AE data the same priority as the primary efficacy outcome	Strongly agree	Agree	Disagree	Strongly disagree	Don't know
	iv	Regulators don't give AE data the same priority as the primary efficacy outcome	Strongly agree	Agree	Disagree	Strongly disagree	Don't know
	v	There are a lack of appropriate analysis methods	Strongly agree	Agree	Disagree	Strongly disagree	Don't know
	vi	There are a lack of examples of the use of appropriate analysis methods in the applied literature	Strongly agree	Agree	Disagree	Strongly disagree	Don't know
9		Are you aware of any published methods specifically to analyse AEs?	Yes	No	Don't know		
		If yes, please specify					
10		If answer is 'yes' to question 9<br>In your opinion why are those methods not being more widely used:					
	i	Available methods are technically too complex	Strongly agree	Agree	Disagree	Strongly disagree	Don't know
	ii	Available methods are too resource intensive	Strongly agree	Agree	Disagree	Strongly disagree	Don't know
	iii	Available methods are not suitable for typical trial sample sizes	Strongly agree	Agree	Disagree	Strongly disagree	Don't know
	iv	Available methods are not suitable for the number of different AEs typically experienced across a trial	Strongly agree	Agree	Disagree	Strongly disagree	Don't know
	v	Available methods are not suitable for typical AE rates observed	Strongly agree	Agree	Disagree	Strongly disagree	Don't know
11		Are there any reasons other than those mention above why those methods are not being more widely used?	Yes	No			
		If yes, please specify					

For peer review only - http://bmjopen.bmj.com/site/about/guidelines.xhtml

Appendix

		Thinking about available methods for AE analysis					
12		How concerned are you about the following:					
	i	Difficulties in interpreting the results/output	Not at all	Slightly concerned	Somewhat concerned	Moderately concerned	Extremely concerned
	ii	Robustness of methods	Not at all	Slightly concerned	Somewhat concerned	Moderately concerned	Extremely concerned
	iii	Acceptability of methods to chief investigator	Not at all	Slightly concerned	Somewhat concerned	Moderately concerned	Extremely concerned
	iv	Acceptability of methods to journal	Not at all	Slightly concerned	Somewhat concerned	Moderately concerned	Extremely concerned
	v	Acceptability of methods to regulator	Not at all	Slightly concerned	Somewhat concerned	Moderately concerned	Extremely concerned
13		Do you have any other thoughts about current practice for AE analysis? If yes, please specify	Yes	No			
14		To what extent do you agree that the following would support a change in AE analysis practice					
	i	Software/code development is needed	Strongly agree	Agree	Disagree	Strongly disagree	Don't know
	ii	Training specifically for AE analysis is needed	Strongly agree	Agree	Disagree	Strongly disagree	Don't know
	iii	Guidance on appropriate AE analysis is needed e.g. case studies, tutorials within open access journals	Strongly agree	Agree	Disagree	Strongly disagree	Don't know
15		Are there any other solutions in addition to those above that would support a change in AE analysis practice? If yes, please specify	Yes	No			
16		When analysing AEs do you present (please select all that apply):					

For peer review only - http://bmjopen.bmj.com/site/about/guidelines.xhtml

Appendix

	i	Number of participants with at least one event	Yes	No
	ii	Number of events	Yes	No
	iii	Other	Yes	No
		If yes, please specify		
17		When analysing AEs which summary statistic would you typically use (please select all that apply)		
	i	Frequency	Yes	No
	ii	Percentage	Yes	No
	iii	Risk difference	Yes	No
	iv	Odds ratio	Yes	No
	v	Risk ratio	Yes	No
	vi	Incidence rate ratio	Yes	No
	vii	Other	Yes	No
		If yes, please specify		
18		In your experience how are AE rates typically compared between treatment groups (please select all that apply)		
	i	Subjective comparison	Yes	No
	ii	Exclusion of null through 95% confidence interval	Yes	No
	iii	Hypothesis test/p-value	Yes	No
	iv	Other	Yes	No
		If yes, please specify		
19		Have you undertaken any specialist AE analysis not mentioned in your previous responses? Please explain your answer. If ‘yes’, please include details of the method(s) used for the analysis performed	Yes	No

For peer review only - http://bmjopen.bmj.com/site/about/guidelines.xhtml

Appendix

Item 2: Text from participant information sheet for CTU participants

Study Title: Statisticians survey on statistical methods for adverse event data analysis in randomised controlled trials

What is the purpose of this study?

This survey will allow an exploration of awareness of statistical methods available to flag AEs as potential adverse drug reactions (ADRs) and identify any potential barriers to their use, as well as gain feedback on ideas for new statistical methods.

Why have I been chosen?

You are eligible to participate in the survey if you satisfy the following inclusion criteria:

i) Your current role is as a senior statistician or equivalent at a UKCRC CTU;
ii) You have experience of planning and preparing final analysis reports for pharmacological RCTs.

We ask you to provide your personal views.

Do I have to take part?

Participation in the study is voluntary. It is up to you to decide whether to take part. If you decide to take part, you are still free to withdraw at any time without having to give a reason. However, retraction or removal of your survey answers is not possible once the 'Submit' button has been selected.

What are the possible disadvantages and risks of taking part?

There are no disadvantages that we are aware of from taking part in this study.

What if something goes wrong?

We are not aware of any risks involved in taking part in this study.

Will my taking part in this study be kept confidential?

All personal records relating to this study will be kept confidential. We will use SurveyMonkey to capture your responses. No personal data will be collected in the survey, as such your

For peer review only - http://bmjopen.bmj.com/site/about/guidelines.xhtml

Appendix

responses to this survey will be anonymous. Responses will be kept in a secure password-protected and encrypted file and stored on Box cloud content management platform. Data in Box is stored securely and automatically backed up. The Box platform is fully General Data Protection Regulation (GDPR) compliant. Upon completion of the study the research data will be uploaded to an approved data-sharing repository. This will be maintained for at least ten years from the time the research study is complete.

What will happen to the results of the research study?

The results of this study will be analysed and published in an open access peer reviewed scientific journal. The work will also be submitted for oral presentation at a range of academic conferences targeting statisticians and the wider clinical trial community. If you would like help in locating and viewing the published results please contact us using the details below. Study data will be stored for ten years post end of study in keeping with Imperial College London research policy.

No identifying data will be published.

Will I receive payment for participating in the study?

You will not be paid for taking part in this study but upon successful completion of the survey, you will be entered into a prize draw for a chance to win £50 worth of Amazon vouchers.

Who is organising and funding the research?

This study is being organised and sponsored by Imperial College London. This study is funded by the National Institute for Health Research (NIHR) (grant reference number DRF-2017-10-131). Please note that the views expressed are those of the author(s) and not necessarily those of the NIHR or the Department of Health and Social Care.

Who has reviewed this study?

This study has been reviewed by the Head of Imperial Clinical Trials Unit and granted ethical approval by the Imperial College Joint Research Compliance Office (JRCO).

What action is required?

For peer review only - http://bmjopen.bmj.com/site/about/guidelines.xhtml

Appendix

Please follow the link in the invitation email to access the survey. We approximate that the survey will take no longer than 15 minutes to complete. You will have an eight-week window to complete the survey. Reminder emails will be sent at week 4 and week 6.

Please note that completing the survey and clicking 'Submit' automatically implies your consent to participate. Participation is voluntary and you are free to withdraw at any point whilst completing the survey. However please note retraction or removal of individual survey answers is not possible once the 'Submit' button has been selected.

Contact information:

Should you have any questions concerning this study, please contact the research team using the details provided below:

Rachel Phillips

Imperial Clinical Trials Unit, Imperial College London, 1st Floor Stadium House, 68 Wood Lane, London, W12 7RH

Email: r.phillips@imperial.ac.uk

Tel: 020 759 49356

We thank you for your consideration to participate in this project.

For peer review only

Appendix

Figure A1: Flow diagram of participation

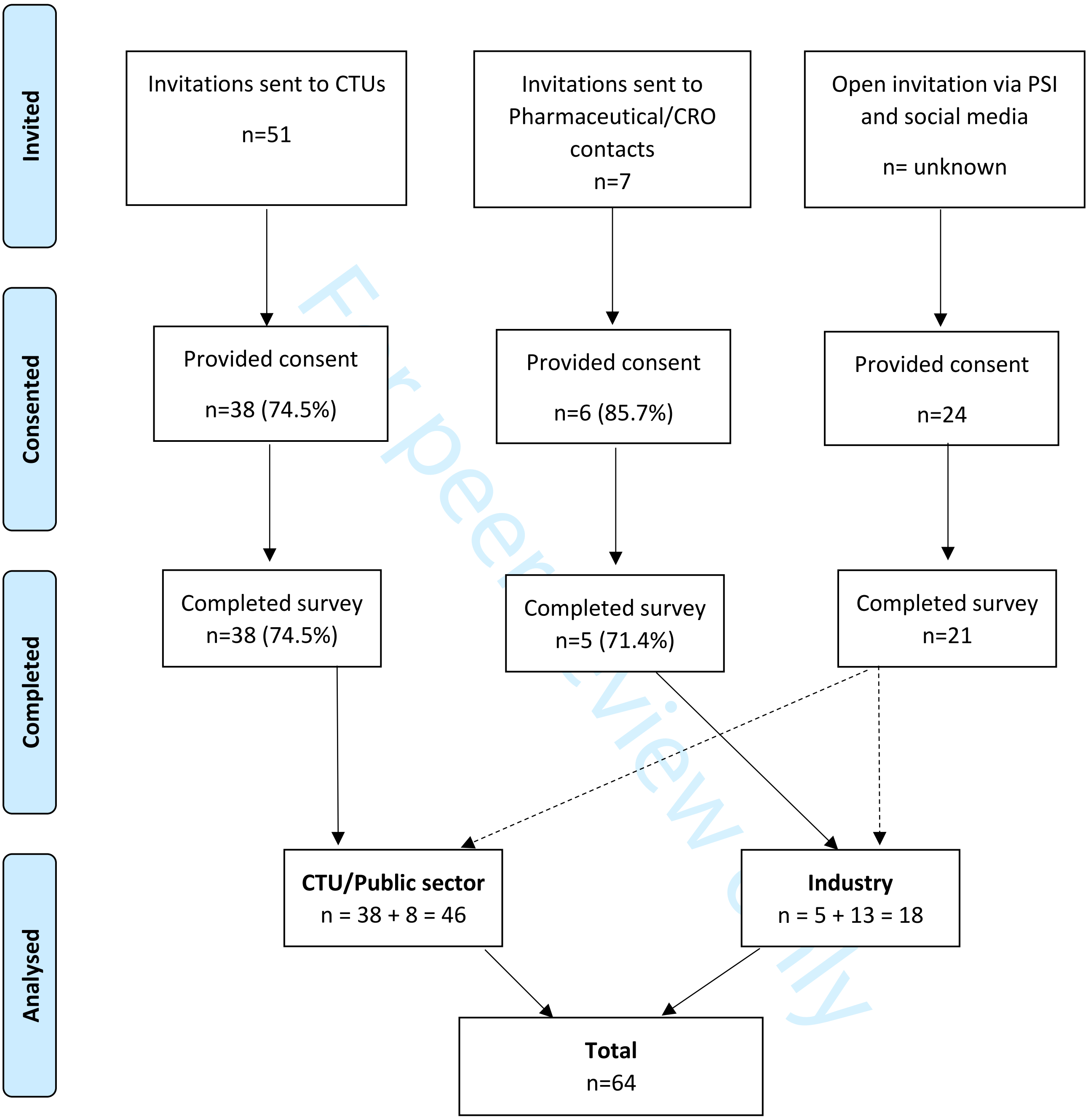

For peer review only - http://bmjopen.bmj.com/site/about/guidelines.xhtml

Appendix

Table A1: Free text comments regarding other information presented on adverse events (AE)

**Other information presented**
We present as a proportion as ITT and also as proportion exposed (requirement for EudraCT). We present specific toxicities and the proportions at each grade.
Number of patients with at least one G3+ events Number of patients with at least one treatment emergent Events of special interest
maximum grade over treatment by subject
Number of participants by worst grade of event (CTCAE), time to specified toxicity event
Number of events by highest CTCAE grade
Frequency of worst CTCAE grade of each AE for each patient during the treatment and follow-up periods
More frequently reported Events by severity SAEs
Relatedness
Number of events presented only for overall summary of aes, teaes, related aes and aes leading to treatment discontinuation. No summary of number of aes by soc and pt
Numbers of patients experiencing 0, 1, 2, ... events
Dependant on the trial. Most commonly the "Number of participants with at least one event" (sometimes by different treatment periods if appropriate). For trials with lengthy "maintenance" type treatments we are moving away from this and may present things like number of AEs per patient or time experiencing certain events.
median number of events in both those experiencing at least one event and out of those randomised.
And percentage by group of course.
Dependant on the trial. Most commonly the "Number of participants with at least one event" (sometimes by different treatment periods if appropriate). For trials with lengthy "maintenance" type treatments we are moving away from this and may present things like number of AEs per patient or time experiencing certain events.
Proportions and %s, making clear what the denominators are
Sometimes both, depending on the AE
In a few occasions, the client asked for confidence intervals, or the prevalence of AEs tested across arms via a Fisher exact test. On only 1 trial in 17 years of time, time to onset analyses were required, with estimation of incidence rates abd associated CI, in person-years.
Rate over the periid of exposure.
Usually both of above and incidence rate. For some events we also include rate per 100 PY
exposure time in years + incidence rates (though this varies from study to study)
incidences per group, incidence rate ratios with uncertainty (depending on the situation)
competing risk analysis

For peer review only - http://bmjopen.bmj.com/site/about/guidelines.xhtml

Appendix

Table A2: Free text comments regarding methods participants are aware of specifically for adverse event (AE) analysis

**Bayesian approaches (n=1):**
"Bayesian methods to analyse low frequency event data."
**Modelling approaches (n=6):**
"I don't think there is anything special about AEs/SAEs that require special methods. Statistical methods for the analysis of events (yes/no) or repeated events accounted for differential follow-up or/and overdispersion already exist in statistical literature (e.g., poisson or negative binomial regression model). of course, it depends on the underlying distribution"
"Classical Poisson/Negative Binomial/ZIP Regression for incidence rates"
"Extreme Value methods"
"…,survival analysis for comparison of treatment and for time to specific event"
"Survival methods"
"GEE"
**Incidence rate (n=5):**
"crude incidence rates, exposure-adjusted incidence rates, mean cumulative function (MCF)"
"Rate analyses,…"
"Cumulative incidence plots, life table plots, and prevalence plots. Many methods not specific to analysis of AEs"
"Incidence rates and confidence intervals (in person-years). Time to onset."
"Rate ratio,…"
**Meta-analysis (n=2):**
"…examples of meta analyses to appropriately analyse AE data"
" Meta analysis of Rare events"
**Graphics (n=2):**
"Cumulative incidence plots, life table plots, and prevalence plots. Many methods not specific to analysis of AEs"
"Graphics for biological parameters (ellipse ci)"
**Theoretical and applied examples (n=6):**
" CLEOPATRA Study Repeated Measures (i.e. not just counting first event)"
"Various methods published by Harry Southworth. These are predominantly useful for pharma trials rather than Phase 4 trials unit trials."
"Volume15, Issue4 Special Issue: Analysis of Adverse Event Data July/August 2016 Pages 297-305"
"http://dx.doi.org/10.1136/bmj.i5078"
"https://onlinelibrary.wiley.com/toc/15391612/2016/15/4"
"possible use of estimands to analyse AEs (for example https://arxiv.org/abs/1805.01834)"
**Other comments:**
"Not meaningfully within an early phase setting, because of sample size. Monitoring based approaches are becoming used and machine learning based methods are available."
"AE tables and summary"
"The statistical literature is awash with methods"
"zz"

For peer review only

Appendix

Table A3: Free text comments regarding participants' use of specialist methods for adverse event (AE) analysis

**Time-to-event analysis (n=2):**
"In characterising safety signals I have used Time to Event, Event rates, prevalence."
"Time-to-event analyses; exposure-adjusted AE rates"
**Data visualisations (n=1):**
"Data visualisation (which is more or less equivalent to frequencies and percentages)"
**Bayesian methods**
"Bayesian methods for sparse adverse events data meta-analysis"
**Incorporating repeated event (n=1):**
"For within-patient repeated events we have produced comparisons with a 2-d frequency table (arm vs # events)"
**Other comments:**
"Not sure I understood what is meant by specialist AE analysis. I used various statistical methods depending on the situation."
"Safety analysis in phase III cancer clinical trial"

Table A4: Reasons specialist adverse event (AE) methods are not used (of participants aware of such methods)

**Reasons for unsuitability. Available methods are:**		**CTU/Public (N=11)**		**Industry (N=12)**		**Overall (N=23)**	
		**n/N**	**%**	**n/N**	**%**	**n/N**	**%**
Technically too complex	Strongly disagree/disagree	8/10	80.0%	6/12	50.0%	14/22	63.6%
	Agree/strongly agree	1/10	10.0%	5/12	41.7%	6/22	27.3%
	Don't know	1/10	10.0%	1/12	8.3%	2/22	9.1%
Too resource intensive	Strongly disagree/disagree	5/10	50.0%	7/12	58.3%	12/22	54.5%
	Agree/strongly agree	5/10	50.0%	5/12	41.7%	10/22	45.5%
Not suitable for typical trial sample sizes	Strongly disagree/disagree	6/10	60.0%	4/12	33.3%	10/22	45.5%
	Agree/strongly agree	3/10	30.0%	5/12	41.7%	8/22	36.4%
	Don't know	1/10	10.0%	3/12	25.0%	4/22	18.2%
Not suitable for the number of different AEs typically experienced across a trial	Strongly disagree/disagree	7/10	70.0%	5/12	41.7%	12/22	54.5%
	Agree/strongly agree	2/10	20.0%	6/12	50.0%	8/22	36.4%
	Don't know	1/10	10.0%	1/12	8.3%	2/22	9.1%
Not suitable for typical AE rates observed	Strongly disagree/disagree	7/10	70.0%	5/12	41.7%	12/22	54.5%
	Agree/strongly agree	3/10	30.0%	7/12	58.3%	10/22	45.5%
Other reasons why those methods are not used	No	0/10	0.0%	3/12	25.0%	3/22	13.6%
	Yes	9/10	90.0%	8/12	66.7%	17/22	77.3%
	Don't know	1/10	10.0%	1/12	8.3%	2/22	9.1%

Acronyms: CTU: Clinical Trial Unit; AE: adverse event

For peer review only - http://bmjopen.bmj.com/site/about/guidelines.xhtml

Appendix

Table A5: Classification of participants' comments on the reasons for a lack of use of specialist methods for adverse event (AE) analysis

**Classification of reasons given for the lack of use of specialist AE analysis methods**	**Participant comment**
**1.Concern with the suitability of methods in relation to trial design characteristics and nature of AE data**	*"…These analyses methods may also not be appropriate if there are doubts about the robustness of AE data…"* (CTU/public sector)
	"The strongest driver is sample size and multiplicity with multiple endpoints, limiting the power of any such analysis." (CTU/public sector)
	"AEs not the primary objective of trial, Pharmaceutical companies focused not on most powerful analyses, issues around multiplicity, recurrent events, low incidence of events" (Industry)
	"…Most AE signals will not result in a statistically significant difference (due to low rates and trial size) and therefore a fear of testing exists, as statisticians we do not want to give the impression that the signal is not real as p>0.05!! Few trials are designed to specifically look at safety, the above methods are used on safety studies." (Industry)
	"…safety analyses typically lack a scientific hypotheses to direct where to look for signals." (CTU/public sector)
	"…2) Multiple testing issues: The multiplicity of AEs that may arise in a RCT makes it also not really appropriate to use statistical tests because of inflated false positive error rates resulting from multiple testings. …3) Even if 1 or 2 AEs of special interest are selected for statistical testing, detecting a statistically significant difference across treatment arms requires to power the trial and calculate the sample size accordingly." (Industry)
	"Appropriateness of methods depends on many factors including underlying distribution, prevalence of repeated events, whether participants were followed up for the same duration, etc. For example, if repeated events are rare and participants were followed up for the same duration then simple number and percentages of participants who experienced at least one event is sufficient. On the contrary, this will obscure the true picture if repeated events are prevalent and participants were follows up for varying periods. So I would say there is a range of statistical methods that are appropriate depending on the situation." (CTU/public sector)
**2. Opposition and a lack of understanding from clinicians**	*"Lack of emphasis placed by clinicians on the need for appropriate statistical methods to analyse adverse events data."* (CTU/public sector)
	"The standard approach of looking at g3+ AEs only is so accepted, there is little motivation to explore other methods. In addition, persuading clinicians to embrace other methods, can be difficult." (CTU/public sector)

For peer review only - http://bmjopen.bmj.com/site/about/guidelines.xhtml

Appendix

	"Most medical leads on clinical trials do not understand statistical analyses and only prefer a list of AEs with their percentages to be presented" (Industry)
	"A tendency to oversimplify reporting of safety signals, to make them easier to understand to non-stats people (e.g. % are easier than incidence rates)" (Industry)
	"The template for reporting AEs is too basic. In the pharmaceutical industry the statisticians have little to no input into the trial paper" (CTU/public sector)
**3. Not deemed to be needed by statisticians**	*"Not required/ wanted."* (CTU/public sector)
	"Don't want to report additional information in CTR" (CTU/public sector)
	"They are perhaps not used as they are no required or appropriate for that type of trial. There is no point in applying a complex method when it is not needed (eg when AEs are collected for a well established drug; when the trial is not attempting to define a safety profile)." (CTU/public sector)
**4. A desire to keep analysis consistent with historical analysis**	*"Easiness to present always the same tables"* (CTU/public sector)
	"1) High level of standardization in reporting of results of RCTs. AE tables are pretty standard and there are requirements to meet ICH3 CSR recommendations…" (Industry)
	"Consistency of analysis across trials in a development programme is often paramount. So, if AEs from a previous study have been analysed using a frequency/percentage approach, so would later trials." (Industry)
**5. Training and resources**	*"Training. Availability of code."* (Industry)

For peer review only - http://bmjopen.bmj.com/site/about/guidelines.xhtml

Appendix

Table A6: Influences the analysis performed

		CTU/Public (N=45)		Industry (N=18)		Overall (N=63)	
**Influence**		**n/N**	**%**	**n/N**	**%**	**n/N**	**%**
Statistician prefers simple approaches e.g. tables of frequencies and percentages	Never/Not very often	13/45	28.9%	7/18	38.9%	20/63	31.7%
	Often/Always	32/45	71.1%	11/18	61.1%	43/63	68.3%
Chief investigator prefers simple approaches e.g. tables of frequencies and percentages	Never/Not very often	9/45	20.0%	2/18	11.1%	11/63	17.5%
	Often/Always	35/45	77.8%	14/18	77.8%	49/63	77.8%
	Don't know	1/45	2.2%	2/18	11.1%	3/63	4.8%
Journal prefers simple approaches e.g. tables of frequencies and percentages	Never/Not very often	12/45	26.7%	7/18	38.9%	19/63	30.2%
	Often/Always	25/45	55.6%	5/18	27.8%	30/63	47.6%
	Don't know	8/45	17.8%	6/18	33.3%	14/63	22.2%
Regulator prefers simple approaches e.g. tables of frequencies and percentages	Never/Not very often	9/45	20.0%	4/18	22.2%	13/63	20.6%
	Often/Always	18/45	40.0%	12/18	66.7%	30/63	47.6%
	Don't know	18/45	40.0%	2/18	11.1%	20/63	31.7%
Trial sample size	Never/Not very often	12/45	26.7%	2/18	11.1%	14/63	22.2%
	Often/Always	31/45	68.9%	15/18	83.3%	46/63	73.0%
	Don't know	2/45	4.4%	1/18	5.6%	3/63	4.8%
The number of different AEs experienced across the trial	Never/Not very often	13/45	28.9%	7/18	38.9%	20/63	31.7%
	Often/Always	31/45	68.9%	10/18	55.6%	41/63	65.1%
	Don't know	1/45	2.2%	1/18	5.6%	2/63	3.2%
AE rates	Never/Not very often	9/45	20.0%	4/18	22.2%	13/63	20.6%
	Often/Always	35/45	77.8%	13/18	72.2%	48/63	76.2%
	Don't know	1/45	2.2%	1/18	5.6%	2/63	3.2%

Acronyms: CTU: Clinical Trial Unit; AE: adverse event

For peer review only

For peer review only - http://bmjopen.bmj.com/site/about/guidelines.xhtml

Appendix

Table A7: Barriers when analysing adverse events (AEs)

		CTU/Public (N=44)		Industry (N=18)		Overall (N=62)	
**Barriers**		n/N	%	n/N	%	n/N	%
Lack of awareness of appropriate methods	Strongly disagree/disagree	11/44	25.0%	7/18	38.9%	18/62	29.0%
	Agree/ Strongly agree	30/44	68.2%	11/18	61.1%	41/62	66.1%
	Don't know	3/44	6.8%	0/18	0.0%	3/62	4.8%
Lack of knowledge to implement appropriate methods	Strongly disagree/disagree	15/44	34.1%	8/18	44.4%	23/62	37.1%
	Agree/ Strongly agree	27/44	61.4%	9/18	50.0%	36/62	58.1%
	Don't know	2/44	4.5%	1/18	5.6%	3/62	4.8%
Lack of training opportunities to learn what methods are appropriate	Strongly disagree/disagree	7/44	15.9%	3/18	16.7%	10/62	16.1%
	Agree/ Strongly agree	34/44	77.3%	15/18	83.3%	49/62	79.0%
	Don't know	3/44	6.8%	0/18	0.0%	3/62	4.8%
Lack of statistical software/code to implement appropriate methods	Strongly disagree/disagree	21/44	47.7%	11/18	61.1%	32/62	51.6%
	Agree/ Strongly agree	14/44	31.8%	7/18	38.9%	21/62	33.9%
	Don't know	9/44	20.5%	0/18	0.0%	9/62	14.5%
Trial sample size	Strongly disagree/disagree	13/44	29.5%	7/18	38.9%	20/62	32.3%
	Agree/ Strongly agree	27/44	61.4%	11/18	61.1%	38/62	61.3%
	Don't know	4/44	9.1%	0/18	0.0%	4/62	6.5%
The number of different AEs experienced across the trial	Strongly disagree/disagree	15/44	34.1%	7/18	38.9%	22/62	35.5%
	Agree/ Strongly agree	27/44	61.4%	11/18	61.1%	38/62	61.3%
	Don't know	2/44	4.5%	0/18	0.0%	2/62	3.2%
AE rates	Strongly disagree/disagree	14/44	31.8%	7/18	38.9%	21/62	33.9%
	Agree/ Strongly agree	29/44	65.9%	11/18	61.1%	40/62	64.5%
	Don't know	1/44	2.3%	0/18	0.0%	1/62	1.6%

Acronyms: CTU: Clinical Trial Unit; AE: adverse event

For peer review only

Appendix

Table A8: Opinions on adverse event (AE) analysis

		CTU/Public (N=44)		Industry (N=18)		Overall (N=62)	
**Opinions**		n/N	%	n/N	%	n/N	%
Statisticians don't give AE data the same priority as the primary efficacy outcome	Strongly disagree/disagree	13/44	29.5%	6/18	33.3%	19/62	30.6%
	Agree/strongly agree	31/44	70.5%	12/18	66.7%	43/62	69.4%
Chief investigators don't give AE data the same priority as the primary efficacy outcome	Strongly disagree/disagree	20/44	45.5%	7/18	38.9%	27/62	43.5%
	Agree/strongly agree	24/44	54.5%	8/18	44.4%	32/62	51.6%
	Don't know	0/44	0.0%	3/18	16.7%	3/62	4.8%
Journals don't give AE data the same priority as the primary efficacy outcome	Strongly disagree/disagree	12/44	27.3%	4/18	22.2%	16/62	25.8%
	Agree/strongly agree	26/44	59.1%	11/18	61.1%	37/62	59.7%
	Don't know	6/44	13.6%	3/18	16.7%	9/62	14.5%
Regulators don't give AE data the same priority as the primary efficacy outcome	Strongly disagree/disagree	25/44	56.8%	14/18	77.8%	39/62	62.9%
	Agree/strongly agree	5/44	11.4%	3/18	16.7%	8/62	12.9%
	Don't know	14/44	31.8%	1/18	5.6%	15/62	24.2%
There are a lack of appropriate analysis methods	Strongly disagree/disagree	15/44	34.1%	8/18	44.4%	23/62	37.1%
	Agree/strongly agree	19/44	43.2%	8/18	44.4%	27/62	43.5%
	Don't know	10/44	22.7%	2/18	11.1%	12/62	19.4%
There are a lack of examples of the use of appropriate analysis methods in the applied literature	Strongly disagree/disagree	5/44	11.4%	1/18	5.6%	6/62	9.7%
	Agree/strongly agree	36/44	81.8%	16/18	88.9%	52/62	83.9%
	Don't know	3/44	6.8%	1/18	5.6%	4/62	6.5%

Acronyms: CTU: Clinical Trial Unit; AE: adverse event

For peer review only

For peer review only - http://bmjopen.bmj.com/site/about/guidelines.xhtml

Appendix

Table A9: Concerns about available methods for adverse event (AE) analysis

		CTU/Public (N=43)		Industry (N=17)		Overall (N=60)	
**Concerns**		**n/N**	**%**	**n/N**	**%**	**n/N**	**%**
Difficulties in interpreting the results/output	Not at all to somewhat concerned	34/43	79.1%	11/17	64.7%	45/60	75.0%
	Moderately to extremely concerned	9/43	20.9%	6/17	35.3%	15/60	25.0%
Robustness of methods	Not at all to somewhat concerned	29/43	67.4%	12/17	70.6%	41/60	68.3%
	Moderately to extremely concerned	14/43	32.6%	5/17	29.4%	19/60	31.7%
Acceptability of methods to chief investigator	Not at all to somewhat concerned	36/43	83.7%	12/17	70.6%	48/60	80.0%
	Moderately to extremely concerned	7/43	16.3%	5/17	29.4%	12/60	20.0%
Acceptability of methods to journal	Not at all to somewhat concerned	34/43	79.1%	14/17	82.4%	48/60	80.0%
	Moderately to extremely concerned	9/43	20.9%	3/17	17.6%	12/60	20.0%
Acceptability of methods to regulator	Not at all to somewhat concerned	33/43	76.7%	4/17	23.5%	37/60	61.7%
	Moderately to extremely concerned	10/43	23.3%	13/17	76.5%	23/60	38.3%

Acronyms: CTU: Clinical Trial Unit; AE: adverse event

For peer review only

For peer review only - http://bmjopen.bmj.com/site/about/guidelines.xhtml

Appendix

Table A10: Solutions to support a change in adverse event (AE) analysis practice

		CTU/Public (N=43)		Industry (N=17)		Overall (N=60)	
**Change**		**n/N**	**%**	**n/N**	**%**	**n/N**	**%**
Software/code development is needed	Strongly disagree/disagree	9/43	20.9%	6/17	35.3%	15/60	25.0%
	Agree/strongly agree	28/43	65.1%	10/17	58.8%	38/60	63.3%
	Don't know	6/43	14.0%	1/17	5.9%	7/60	11.7%
Training specifically for AE analysis is needed	Strongly disagree/disagree	1/43	2.3%	1/17	5.9%	2/60	3.3%
	Agree/strongly agree	42/43	97.7%	16/17	94.1%	58/60	96.7%
Guidance on appropriate AE analysis is needed e.g. case studies, tutorials within open access journals	Strongly disagree/disagree	0/43	0.0%	0/17	0.0%	0/60	0.0%
	Agree/strongly agree	43/43	100.0%	17/17	100.0%	60/60	100.0%
Are there any other solutions in addition to those stated above that would support a change in AE analysis practice?	No	34/43	79.1%	7/17	41.2%	41/60	68.3%
	Yes	9/43	20.9%	10/17	58.8%	19/60	31.7%

Acronyms: CTU: Clinical Trial Unit; AE: adverse event

For peer review only

Appendix

Table A11: Classification of participants' comments on solutions to support change in adverse event (AE) analysis practices

Classification of solutions to support a change in AE analysis practice	Participant comment
**1. Improved standards or calls for changes from journals, registries and regulators**	*"Influencing journals to pay more attention to this"* (CTU)
	"…we presented incidences because they represented a fairer picture due to differential follow-up and repeated incidences per person. The reviewer and the editor said they prefer proportions and don't understand what we presented. I explained in lay terms and pushed back their request because it was flawed. This shows that Statisticians can defend a certain position and educate others even if they have their own preferences.<br><br>*Regulatory repositories/registries such as EUDRACT has a fixed format of presenting results so you have to go with what is required even though you know it's flawed in certain situation. Flexibility of such registries is very important to allow people to present both proportions and incidences where appropriate."* (CTU)
	"Asked by the authorities" (Industry)
	"Strong regulatory direction is always good for changing practices within the industry!" (Industry)
	"engaging the … regulators" (Industry)
	"The biggest driver of a change in behaviour is usually a regulator requesting it." (Industry)
	"Regulators to be more demanding in analytical approaches, don't require more than summaries. That's far removed from discussions on efficacy" (Industry)
	"Would have to be able to upload the results to EUDRACT for CTIMPS." (CTU)
**2. Development of guidance, education and engaging with the medical community**	*"Best practice guidance although that would depend on trial type and phase, sample size, whether only SAEs/related AEs are being captured/important, ... particularly important to reflect on complex interventions vs CTIMP, etc"* (CTU)
	"There needs to be consensus that a change is needed. What are the issues in current AE reporting? There needs to be better guidance re collection of AE data. Can we collect it in a more robust way? We need to differentiate between examining pre-specified hypotheses and trying to identify issues we don't know about (eg in early phase trials). We need agreement re standards for different phases and types of trials (eg Phase 1 vs Phase 4, explanatory vs

Appendix

	pragmatic, regulatory submissions vs investigator led exploratory trials on marketed products)" (CTU)
	"Published case studies" *(Industry)*
	"engaging the medical community …. and Better education on the pros of using proper stats methodology. If the benefits of using effective statistical analysis methods over frequencies and percentages can be demonstrated, there might be more interest" (Industry)
	"demonstration of the benefits of these methods over existing ones, and when they are appropriate" (CTU)
	"Open discussions with clinical community (e.g. open forums, etc) on alternative methods to avoid them being scared off" (Industry)
	More focus on safety analyses in the E9 addendum" (Industry)
	"Application of CONSORT harms" (CTU)
	"Evolution of standard reporting requirements in clinical trials (ICH E3, and maybe CONSORT Statement ?)" (Industry)
**3. Analysis**	*"IPD meta analysis of AEs" (CTU)*
	"In addition to 'methods' there perhaps need to be discussion about populations/datasets on which to base AE analyses." (CTU)
	"Inferential analysis based on small numbers of adverse events, but of great influence on the patient health." (Industry)

For peer review only - http://bmjopen.bmj.com/site/about/guidelines.xhtml

Appendix

Table A12: Classification of participants' general comments raised regarding adverse event (AE) analysis practices

**Classification of suggestions raised for AE analysis**	**Participant comment**
**1. Minimum summary information participants would expect to be reported for AEs**	*"Different analysis approach are useful for interpretation when reporting AEs/SAEs. As a starting point, I would like to know the numbers and proportions experiencing at least one SAE by group, between group differences with uncertainty. In addition, I would like to know the incidences per group and incidence rate ratio with uncertainty. The later is not always necessary depending on the situation.."* (CTU)
	"I think in general reporting numbers and percentages is appropriate. The argument being that, if we were clinicians or patients we would want to know what is the chances of me having this event and how bad will it get, which is essentially what the frequency tables give you." (CTU)
**2. Changes that could or have been made to analysis practice**	*"No best practice guidance although revised CONSORT does help remind of importance of AE reporting"* (CTU)
	"There was a great talk at SCT 2017 on using graphical methods to summarise AEs and I have been trying to implement graphical methods to summarise the many dimensions of AE reporting as a way forward" (CTU)
	"Use of graphical methods in reporting to compare treatments ought to be standard, as per BMJ article. They are easy enough to apply…<br>*…The format of the source data, typically free text, is a pain to code into MedDRA. Methods to make this easier would be very valuable: some sort of AI machine learning maybe?...*<br>*…Meta-analysis should be very important to apply to safety data, given how under-powered individual trials may be for safety comparisons. Finding tools to automate, maybe using results entered on EudraCT might be an idea."* (CTU)
	"We have increased our use of graphics. I find benefit risk plots a very powerful way of summarising data. Allows key efficacy and safety to be displayed on one page and is a really useful summary of a drug's profile." (Industry)
	"Current practice will need to turn to methods of detecting signals as real-time data come from trials." (Industry)
	"Signal detection method" (CTU)

For peer review only - http://bmjopen.bmj.com/site/about/guidelines.xhtml

Appendix

	"I'm interested in knowing more about risk factors of occurrence of serious or really frequent AEs of chemotherapies, beyond receiving protocol x." (Industry)
	"… not many medical leads understand statistical analysis of AEs or count or rate data and only insist on percentages and frequencies. Better methods exist but are not utilised due to lack of knowledge of PIs or medical advisors" (Industry)
**3. Concerns about the quality and collection of AE data**	*"This definitely gets overlooked. I always worry about how systematically the data have been collected too as well as the validity of lumping very different events together in the same analysis."* (CTU)
	"I think a big factor in what analysis we choose is how the data is collected. If the data is not detailed enough some only simple methods may be appropriate - this has often been my feeling when analysing our data. this may change in current/future trials as we are changing how we collect some AE data" (CTU)
	"My concerns start with the quality of AE data collected. Is it complete? Is it robust? There is recall bias, variability between centres, investigators etc. There may also be variability with respect to coding. We all have experience of stating up front what should NOT be recorded as AE, to see such things recorded multiple times. One of my major concerns is the listing of AEs each with associated p-values (obviously the CI would insist on this and not the statistician). Completely meaningless as it doesn't take into account sample size, rate, number of events within a participants, severity of event etc etc. Also of concern is the use of more complex methodologies on such data as it implies that the data are robust. I think that the simple approach is often acceptable so long as the data are presented in different ways (see Q16). The main issue is about defining what you are trying to detect from the collection of AE data. If we can do this better then perhaps additional required methodology will come." (CTU)
**4. General comments and criticisms about current AE analysis and reporting practices**	*"Somewhat arbitrary grouping of AEs. Not always clear whether numbers are subjects or events are presented in published papers."* (CTU)
	"In my 8.5 years of experience I have not seen many studies where they have spoken much about AE data analysis." (Industry)
	"People do the most powerful test for efficacy - no barrel goes unscraped - and the least powerful for safety" (CTU)
	"It can be improved!" (Industry)

For peer review only - http://bmjopen.bmj.com/site/about/guidelines.xhtml

Checklist for Reporting Results of Internet E-Surveys (CHERRIES)

	Checklist for Reporting Results of Internet E-Surveys (CHERRIES)		
Item Category	***Checklist Item***	***Explanation***	***Section/Page reported***
**Design**	Describe survey design	Describe target population, sample frame. Is the sample a convenience sample? (In "open" surveys this is most likely.)	Methods - sampling and recruitment. Page 7/8
**IRB (Institutional Review Board) approval and informed consent process**	IRB approval	Mention whether the study has been approved by an IRB.	Ethics. Page 22
	Informed consent	Describe the informed consent process. Where were the participants told the length of time of the survey, which data were stored and where and for how long, who the investigator was, and the purpose of the study?	Methods - sampling and recruitment. Page 8
	Data protection	If any personal information was collected or stored, describe what mechanisms were used to protect unauthorized access.	Appendix item 2 - participant information sheet
**Development and pre-testing**	Development and testing	State how the survey was developed, including whether the usability and technical functionality of the electronic questionnaire had been tested before fielding the questionnaire.	Methods - survey development. Page 7
**Recruitment process and description of the sample having access to the questionnaire**	Open survey versus closed survey	An "open survey" is a survey open for each visitor of a site, while a closed survey is only open to a sample which the investigator knows (password-protected survey).	Methods - sampling and recruitment. Page 7/8
	Contact mode	Indicate whether or not the initial contact with the potential participants was made on the Internet. (Investigators may also send out questionnaires by mail and allow for Web-based data entry.)	Methods - sampling and recruitment. Page 7/8
	Advertising the survey	How/where was the survey announced or advertised? Some examples are offline media (newspapers), or online (mailing lists – If yes, which ones?) or banner ads (Where were these banner ads posted and what did they look like?). It is important to know the wording of the announcement as it will heavily influence who chooses to participate. Ideally the survey announcement should be published as an appendix.	Methods - sampling and recruitment. Page 7/8

For peer review only

**Survey administration**	Web/E-mail	State the type of e-survey (eg, one posted on a Web site, or one sent out through e-mail). If it is an e-mail survey, were the responses entered manually into a database, or was there an automatic method for capturing responses?	Methods - sampling and recruitment. Page 7/8
	Context	Describe the Web site (for mailing list/newsgroup) in which the survey was posted. What is the Web site about, who is visiting it, what are visitors normally looking for? Discuss to what degree the content of the Web site could pre-select the sample or influence the results. For example, a survey about vaccination on a anti-immunization Web site will have different results from a Web survey conducted on a government Web site	Discussion - Strengths and limitations. Page 20/21
	Mandatory/voluntary	Was it a mandatory survey to be filled in by every visitor who wanted to enter the Web site, or was it a voluntary survey?	Not applicable
	Incentives	Were any incentives offered (eg, monetary, prizes, or non-monetary incentives such as an offer to provide the survey results)?	Methods - sampling and recruitment. Page 7/8
	Time/Date	In what timeframe were the data collected?	Methods - sampling and recruitment. Page 7/8
	Randomization of items or questionnaires	To prevent biases items can be randomized or alternated.	Not applicable
	Adaptive questioning	Use adaptive questioning (certain items, or only conditionally displayed based on responses to other items) to reduce number and complexity of the questions.	Not applicable
	Number of Items	What was the number of questionnaire items per page? The number of items is an important factor for the completion rate.	Appendix item 1 - survey questions
	Number of screens (pages)	Over how many pages was the questionnaire distributed? The number of items is an important factor for the completion rate.	Not reported

For peer review only

	Completeness check	It is technically possible to do consistency or completeness checks before the questionnaire is submitted. Was this done, and if "yes", how (usually JAVAScript)? An alternative is to check for completeness after the questionnaire has been submitted (and highlight mandatory items). If this has been done, it should be reported. All items should provide a non-response option such as "not applicable" or "rather not say", and selection of one response option should be enforced.	Appendix item 1 - survey questions
	Review step	State whether respondents were able to review and change their answers (eg, through a Back button or a Review step which displays a summary of the responses and asks the respondents if they are correct).	Not reported
**Response rates**	Unique site visitor	If you provide view rates or participation rates, you need to define how you determined a unique visitor. There are different techniques available, based on IP addresses or cookies or both.	Not applicable
	View rate (Ratio of unique survey visitors/unique site visitors)	Requires counting unique visitors to the first page of the survey, divided by the number of unique site visitors (not page views!). It is not unusual to have view rates of less than 0.1 % if the survey is voluntary.	Not applicable
	Participation rate (Ratio of unique visitors who agreed to participate/unique first survey page visitors)	Count the unique number of people who filled in the first survey page (or agreed to participate, for example by checking a checkbox), divided by visitors who visit the first page of the survey (or the informed consents page, if present). This can also be called "recruitment" rate.	
	Completion rate (Ratio of users who finished the survey/users who agreed to participate)	The number of people submitting the last questionnaire page, divided by the number of people who agreed to participate (or submitted the first survey page). This is only relevant if there is a separate "informed consent" page or if the survey goes over several pages. This is a measure for attrition. Note that "completion" can involve leaving questionnaire items blank. This is not a measure for how completely questionnaires were filled in. (If you need a measure for this, use the word "completeness rate".)	Results - participant flow and Appendix Figure A1. Page 9/10

For peer review only

**Preventing multiple entries from the same individual**	Cookies used	Indicate whether cookies were used to assign a unique user identifier to each client computer. If so, mention the page on which the cookie was set and read, and how long the cookie was valid. Were duplicate entries avoided by preventing users access to the survey twice; or were duplicate database entries having the same user ID eliminated before analysis? In the latter case, which entries were kept for analysis (eg, the first entry or the most recent)?	Not applicable
	IP check	Indicate whether the IP address of the client computer was used to identify potential duplicate entries from the same user. If so, mention the period of time for which no two entries from the same IP address were allowed (eg, 24 hours). Were duplicate entries	Not applicable
	Log file analysis	Indicate whether other techniques to analyze the log file for identification of multiple entries were used. If so, please describe.	Not applicable
	Registration	In "closed" (non-open) surveys, users need to login first and it is easier to prevent duplicate entries from the same user. Describe how this was done. For example, was the survey never displayed a second time once the user had filled it in, or was the username stored together with the survey results and later eliminated? If the latter, which entries were kept for analysis (eg, the first entry or the most recent)?	Discussion - Strengths and limitations. Page 20/21
**Analysis**	Handling of incomplete questionnaires	Were only completed questionnaires analyzed? Were questionnaires which terminated early (where, for example, users did not go through all questionnaire pages) also analyzed?	No. Results section reflects this.
	Questionnaires submitted with an atypical timestamp	Some investigators may measure the time people needed to fill in a questionnaire and exclude questionnaires that were submitted too soon. Specify the timeframe that was used as a cut-off point, and describe how this point was determined.	Not applicable
	Statistical correction	Indicate whether any methods such as weighting of items or propensity scores have been used to adjust for the non-representative sample; if so, please describe the methods.	Not applicable

For peer review only
